# Adding Diversity to a Diruthenium Biscyclopentadienyl Scaffold via Alkyne Incorporation: Synthesis and Biological Studies

**DOI:** 10.1021/acs.inorgchem.3c01644

**Published:** 2023-07-21

**Authors:** Giulio Bresciani, Serena Boni, Tiziana Funaioli, Stefano Zacchini, Guido Pampaloni, Natalia Busto, Tarita Biver, Fabio Marchetti

**Affiliations:** † University of Pisa, Dipartimento di Chimica e Chimica Industriale, Via G. Moruzzi 13, I-56124 Pisa, Italy; ‡ University of Bologna, Dipartimento di Chimica Industriale “Toso Montanari”, Viale del Risorgimento 4, I-40136 Bologna, Italy; § University of Burgos, Departamento de Química, Plaza Misael Bañuelos s/n, 09001 Burgos, Spain

## Abstract

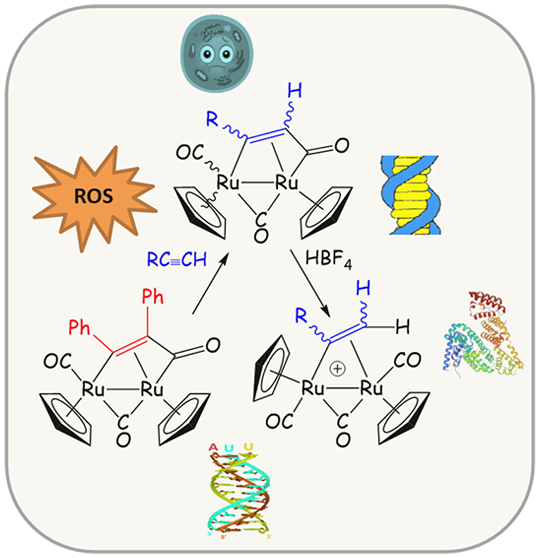

We report the synthesis and the assessment of the anticancer potential of two series of diruthenium biscyclopentadienyl carbonyl complexes. Novel dimetallacyclopentenone compounds (**2**–**4**) were obtained (45–92% yields) from the thermal reaction (PhCCPh exchange) of [Ru_2_Cp_2_(CO)(μ-CO){μ-η^1^:η^3^-C(Ph)=C(Ph)C(=O)}], **1**, with alkynes HCCR [R = C_5_H_4_FeCp (Fc), 3-C_6_H_4_(Asp), 2-naphthyl; Cp = η^5^-C_5_H_5_, Asp = OC(O)-2-C_6_H_4_C(O)Me]. Protonation of **1**–**3** by HBF_4_ afforded the corresponding μ-alkenyl derivatives **5**–**7**, in 40–86% yields. All products were characterized by IR and NMR spectroscopy; moreover, cyclic voltammetry (**1**, **2**, **5**, **7**) and single-crystal X-ray diffraction (**5**, **7**) analyses were performed on representative compounds. Complexes **5**–**7** revealed a cytotoxic activity comparable to that of cisplatin in A549 (lung adenocarcinoma), SW480 (colon adenocarcinoma), and ovarian (A2780) cancer cell lines, and **2**, **5**, **6**, and **7** overcame cisplatin resistance in A2780cis cells. Complexes **2**, **5**, and **7** (but not the aspirin derivative **6**) induced an increase in intracellular ROS levels. Otherwise, **6** strongly stabilizes and elongates natural DNA (from calf thymus, CT-DNA), suggesting a possible intercalation binding mode, whereas **5** is less effective in binding CT-DNA, and **7** is ineffective. This trend is reversed concerning RNA, and in particular, **7** is able to bind poly(rA)poly(rU) showing selectivity for this nucleic acid. Complexes **5**–**7** can interact with the albumin protein with a thermodynamic signature dominated by hydrophobic interactions. Overall, we show that organometallic species based on the Ru_2_Cp_2_(CO)_*x*_ scaffold (*x* = 2, 3) are active against cancer cells, with different incorporated fragments influencing the interactions with nucleic acids and the production of ROS.

## Abstract 2

.

## Introduction

**Figure 1:**
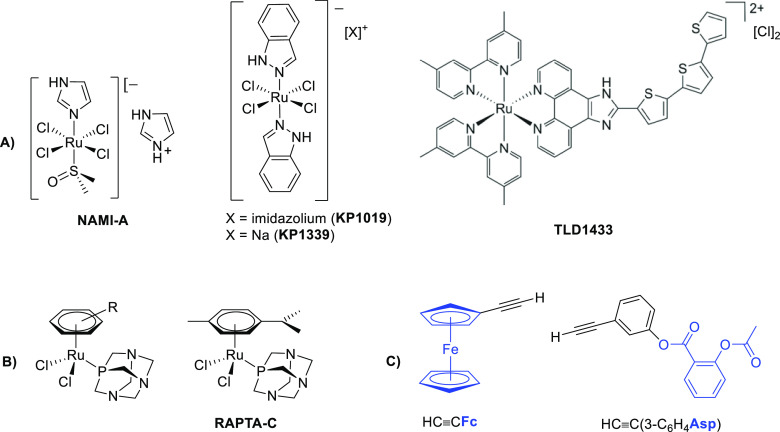
Lead ruthenium compounds with documented anticancer activity. (A) Complexes investigated in clinical trials: **NAMI-A**, **KP1019**, **KP1339**, and **TLD1433**. (B) Generic structure of RAPTA complexes and **RAPTA-C**. (C) Alkynes used in this work containing bioactive groups (ferrocenyl and the aspirin skeleton, in blue).

In the search for new and effective anticancer drugs overcoming some limitations associated with platinum drugs employed against several types of tumors, ruthenium-based candidates have aroused a great interest in the last two decades with a few of them tested in the clinic (Figure 1A).^1−3^ Organometallic complexes based on the [Ru^II^(η^6^-arene)] core have been widely investigated,^4−6^ and within this category, RAPTA compounds display a promising potential and are currently pointing to clinical trials (Figure 1B).^7−9^

Since a bimetallic scaffold may provide significant advantages with respect to related monometallic species, a diversity of dinuclear ruthenium complexes have also been considered in the anticancer field.^10−18^ In general, the bimetallic assembly consists of two monoruthenium units connected via a suitable bidentate ligand acting as a linker,^19,20^ while diruthenium (or polyruthenium) structures containing metal–metal bond(s) have been almost unexplored.^21^ Our attention turned to the dinuclear commercial compound [Ru_2_Cp_2_(CO)_4_], which has been employed as a convenient starting material for classical organodiruthenium chemistry.^22−24^ Biological studies on [Ru_2_Cp_2_(CO)_4_] and its dinuclear derivatives are still missing in the literature, and we considered this research worthy of development for several reasons. The possible dissociation of the CO ligands may be enabled by the interaction with suitable biosubstrates and might contribute to the biological activity, according to the fact that carbon monoxide exerts important pharmacological effects when administered in low doses as delivered to the biotarget through metal–carbonyl compounds (CORMs).^25−27^ In addition, the bimetallic scaffold offers cooperative effects arising from the two adjacent ruthenium centers, thus allowing the construction of functionalized hydrocarbyl ligands on one bridging site, otherwise hardly accessible on related monoruthenium compounds.^22,24,28^ This synthetic approach is potentially useful in view of drug development, since it can be exploited to incorporate bioactive groups playing some biological role and/or finely modulate the physicochemical properties of the compounds. In principle, if the hydrocarbyl fragment coordinated to the {Ru_2_Cp_2_(CO)_*x*_} scaffold possesses a net positive charge, this may partially compensate for the lipophilicity of the structure, resulting in an enhancement of the water solubility of the complex.^28^

In the present manuscript, we describe the synthesis of new diruthenium complexes by means of reactions involving different alkynes, including ferrocenyl [Fc = (η^5^-C_5_H_4_)FeCp] and the skeleton of aspirin (Figure 1C), and an evaluation of the anticancer potential of the products. Note that the ferrocene scaffold may contribute an additional antitumoral effect related to the Fe^II^ to Fe^III^ oxidation occurring intracellularly, resulting in an inbalance of the cell redox homeostasis.^29−31^ On the other hand, acetylsalicylic acid (aspirin, **Asp**H) is one of the most popular medicines in the world and possesses analgesic, antipyretic, and anti-inflammatory properties, which have been associated with the inactivation of COX-1 and COX-2 enzymes. Furthermore, it has been recently discussed that aspirin possesses anticancer properties.^32^ The inclusion of aspirin within platinum(IV),^33^ diiron complexes,^34^ and other metallic species^35^ has been demonstrated to play synergistic effects in cancer cells.

## Results and Discussion

### Synthesis and Structural Characterization of Diruthenium Complexes

**Scheme 1:**
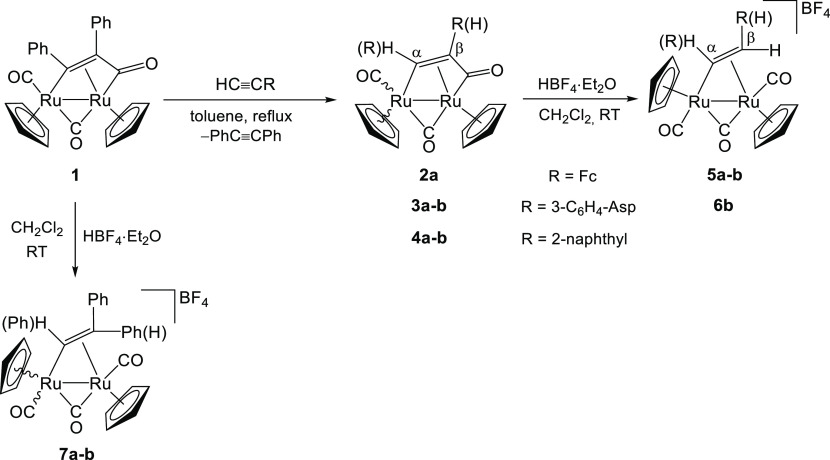
Synthesis of New Diruthenacyclopentenone (**2**–**4**) and μ-Alkenyl Complexes (**5**–**7**) **2a**–**7a** refer to the geometric isomer with H on an α carbon, while **2b**–**7b** refer to the alternative geometric isomer with substituents given in parentheses (H on β carbon).

The dimetallacyclopentenone complex **1** was prepared according to a recently optimized procedure from commercial [Ru_2_Cp_2_(CO)_4_].^36^ It was reported that **1** undergoes thermal exchange of the {PhCCPh} fragment with other alkynes, according to a general reaction.^22,23^ Here, this strategy was exploited to incorporate unprecedented amounts of alkynes within the diruthenium scaffold. Thus, the reactions of **1** with a 4-fold excess of alkynyl-ferrocene, 3-ethynylphenyl 2-acetoxybenzoate,^37,38^ and 2-ethynylnaphthalene were conducted in toluene at reflux temperature and afforded the novel diruthenacyclopentenones **2**–**4** in moderate to high yields (Scheme 1).

The IR spectra of **2**–**4** (in CH_2_Cl_2_ solution) share a common pattern with three absorptions, attributed to the terminal and bridging carbonyl ligands, and to the acyl group (e.g., for **2**, at 1972, 1800, and 1748 cm^–1^, respectively).^39^ The NMR spectra of **2** (acetone-*d*_6_ solution; Figures S1–S2) consist of single sets of resonances, ascribable to the geometric isomer **2a** (alkyne incorporation occurs by placing R = Fc on the β carbon of the dimetallacycle). In fact, salient NMR features are represented by the resonances related to the alkenyl CH moiety, occurring at typically low fields [δ(^1^H) = 10.93 ppm, δ(^13^C) = 150.2 ppm], in agreement with its position close to ruthenium centers and approximately equidistant from them.^40−44^ The ^1^H resonances for the Cp ligands occur at 5.60 and 5.04 ppm, and these values are in alignment with the *cis* mutual orientation of the Cp rings, with respect to the Ru–Ru axis.^45^ In the ^13^C NMR spectra, the carbonyl groups resonate at 236.6 ppm (bridging CO ligand), 220.9 ppm (acyl), and 201.6 ppm (terminal CO ligand).

The NMR spectra of **3**–**4** (Figures S3–S6) reveal the presence of mixtures of two regioisomers, *apparently* originating from the two possible regiochemical modes of insertion of the alkyne fragment {HCCR} within the bridging hydrocarbyl ligand (Scheme 1). The occurrence of one single isomer in **2** (**2a**) may be a consequence of a favorable combination of steric and electronic effects arising from the ferrocenyl group. Complex **3** exists in solution as a mixture of **3a** and **3b** (3:1 molar ratio), with the alkenyl proton resonating at 11.11 ppm in **3a** (CH in the α position) and at 3.37 ppm in **3b** (CH in the β position). Similarly, a mixture of comparable amounts of **4a** and **4b** was obtained, and two forms of **4a** were additionally detected, which we attributed to *cis*–*trans* isomers with a prevalence of the *cis* isomer.

**Scheme 2:**
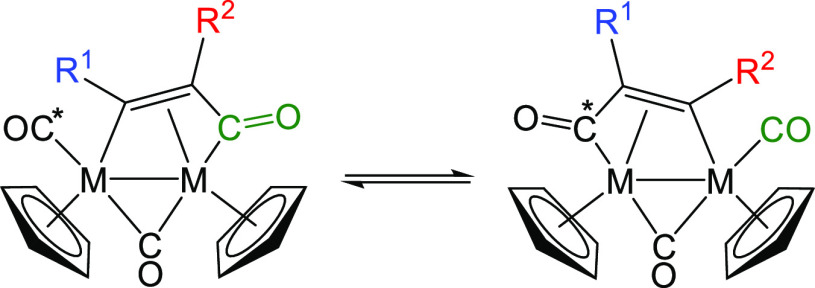
Fluxional Process Proposed for Dimetallacyclopentenone Complexes in Solution The fragment {R^1^CCR^2^} slides back and forth between the acyl CO (in green) and the terminal CO (with the asterisk). M = Fe, Ru; R^1^, R^2^ = Ph, Me, CO_2_Me.^22,23^

It was previously demonstrated^22,23^ on related diruthenacyclopentenone compounds that the two regioisomers originated from two different alkyne insertion modes interconverting into each other with a rate that is slow on the NMR time scale. The mechanism of this interconversion consists of the reversible ejection from the dimetallacycle of the acyl group, which is replaced on the other side of the molecule by the terminal CO ligand (Scheme 2). To give insight into this point, we performed variable temperature ^1^H NMR experiments on **2** and **3** (in toluene-*d*_8_ solutions). In agreement with former findings on **1**,^23^ a progressive broadening of the signals in the spectra of **2a** and **3a**–**b** was observed upon increasing the temperature (Figures S10–S11). This phenomenon especially affects the resonances related to the alkenyl CH and the cyclopentadienyl rings and, in **2a**, is more pronounced for the C_5_ rings belonging to the ferrocenyl. In **3a**/**3b**, coalescence of Cp ligands occurs when the temperature exceeds 343 K, rendering the two isomeric forms indistinguishable.

Dimetallacyclopentenone complexes are prone to react with Brønsted acids to give alkenyl derivatives via C–C(O) bond cleavage.^46,47^ Thus, with a view to biological applications, we allowed *neutral* complexes **1**–**3** to react with HBF_4_ in dichloromethane, affording *ionic* products **5**–**7** in variable yields (Scheme 1). The protonation reaction of **4** was unclean, leading to a complex mixture of carbonyl products (according to IR spectroscopy), which could not be separated/identified. Note that the association of a net positive charge with an organometallic structure is expected to increase the hydrophilicity and the water solubility of the resulting complexes, which is a desirable prerequisite for an anticancer drug candidate.^48−51^

**Figure 2:**
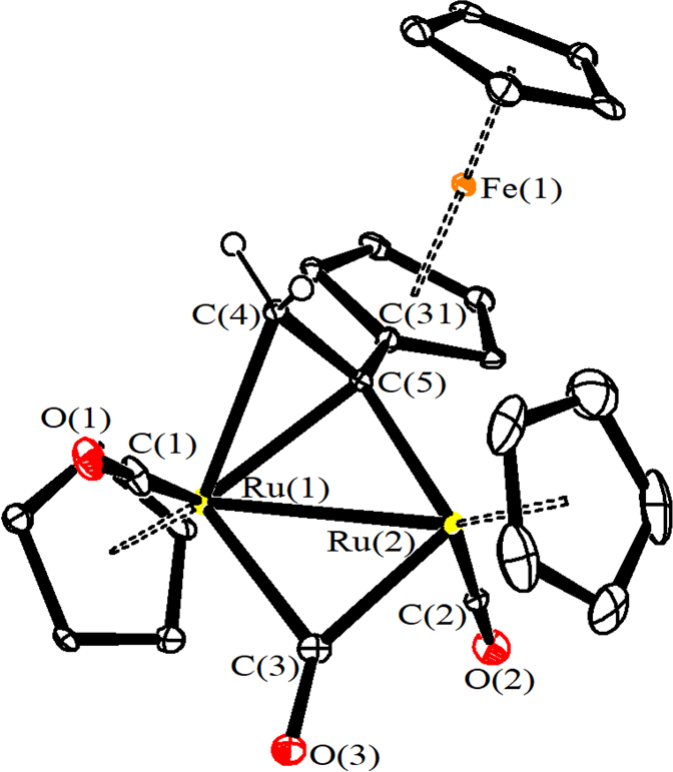
View of the molecular structure of the cation of **5b**. Displacement ellipsoids are at the 30% probability level. H atoms have been omitted for clarity, expect those bonded to C(4).

**Figure 3:**
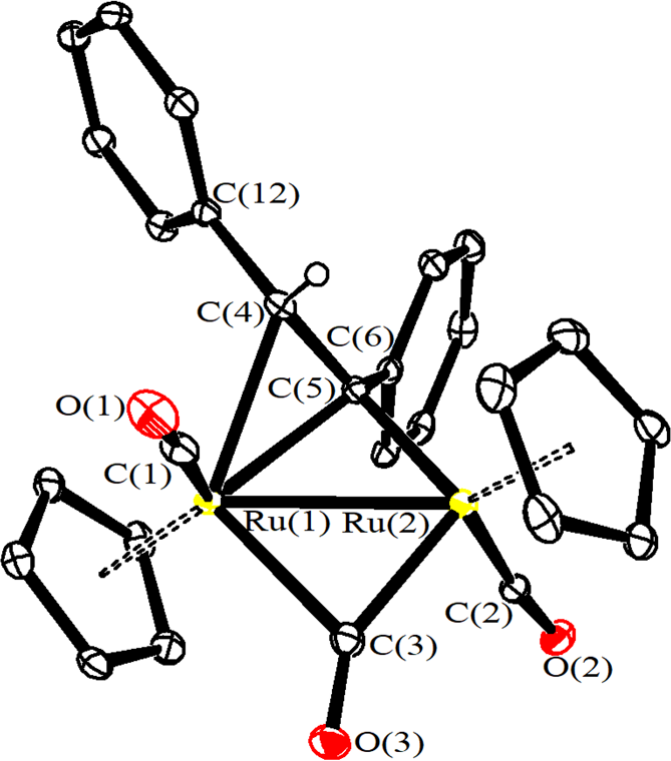
View of the molecular structure of the cation of **7b**. Displacement ellipsoids are at the 30% probability level. H atoms have been omitted for clarity, except that bonded to C(4).

The structures of **5b** and **7b** were ascertained by single-crystal X-ray diffraction studies (Figures 2 and 3 and Table 1). In both cases, the cations are composed of a *trans*-[Ru_2_Cp_2_(CO)_2_(μ-CO)] core bonded to a μ-η^1^:η^2^-alkenyl ligand, and the bonding parameters related to the core match those reported for analogous diruthenium complexes.^52−55^ Regarding the bridging hydrocarbyl ligand, the ferrocenyl substituent in **5b** is placed on the α carbon, which is bridged to the metal centers. The C(4)–C(5) alkenyl bond [1.413(13) and 1.418(4) Å for **5b** and **7b**, respectively) is significantly elongated compared to a normal C=C bond, in view of its coordination to ruthenium. The edge-bridging CO ligand displays a marked asymmetry, with the Ru(2)–C(3) contact [1.990(9) and 1.957(3) Å for **5b** and **7b**, respectively] considerably shorter than Ru(1)–C(3) [2.156(9) and 2.254(3) Å for **5b** and **7b**, respectively]. This is due to the fact that Ru(1) is bonded to both C(4) and C(5), whereas Ru(2) is bonded only to C(4). A slight asymmetry is observed also for the bridging alkenyl [Ru(1)–C(5) 2.218(8) and 2.182(2) Å; Ru(2)–C(5) 2.073(8) and 2.085(3) Å, respectively, for **5b** and **7b**].

**Table 1:** Selected Bond Lengths (Å) and Angles (deg) for **5b** and **7b**

	**5b**	**7b**
Ru(1)–Ru(2)	2.7580(10)	2.7813(3)
Ru(1)–C(1)	1.886(10)	1.893(3)
Ru(2)–C(2)	1.879(10)	1.885(3)
Ru(1)–C(3)	2.156(9)	2.254(3)
Ru(2)–C(3)	1.990(9)	1.957(3)
Ru(1)–C(4)	2.236(9)	2.299(2)
Ru(1)–C(5)	2.218(8)	2.182(2)
Ru(2)–C(5)	2.073(8)	2.085(3)
C(1)–O(1)	1.133(12)	1.139(3)
C(2)–O(2)	1.140(12)	1.139(3)
C(3)–O(3)	1.152(11)	1.160(3)
C(4)–C(5)	1.413(13)	1.418(4)
Ru(1)–C(1)–O(1)	177.9(10)	176.5(3)
Ru(2)–C(2)–O(2)	176.4(9)	171.4(2)
Ru(1)–C(3)–Ru(2)	83.3(3)	82.35(10)
Ru(1)–C(5)–Ru(2)	79.9(3)	81.33(9)
Ru(1)–C(4)–C(5)	70.8(5)	67.12(14)

The IR spectra of **5**–**7** (CH_2_Cl_2_ solutions) show three bands ascribable to the two terminal and one semibridging carbonyl ligands, e.g., at 2035, 2016, and 1871 cm^–1^ in the case of **7**. The CO semibridging coordination, evidenced in solution by IR spectroscopy,^56^ is coherent with the X-ray data collected for two representative compounds in the solid state (see above). Moreover, the two carboxylate groups in **6** manifest themselves with two strong absorptions occurring at 1762 and 1743 cm^–1^. The NMR spectra of **5**–**7**, recorded in acetone-*d*_6_ solutions at room temperature, displayed only broad signals that could not be attributed, suggesting the occurrence of some fluxional process. Thus, ^1^H NMR analyses were repeated at low temperatures (183–223 K) revealing, in two cases over three, pairs of geometric isomers differing in the position of one alkenyl substituent, i.e., **5a** and **5b**; **6b**; and **7a** and **7b** (Figures S7–S9). The detected isomerism appears as the consequence of the structural dynamism affecting the dimetallacyclopentenone precursors **1**–**3** (Scheme 2). On the other hand, the faster fluxionality observed in **5**–**7** is attributable to the oscillation of the alkenyl bridge between the two metal centers, in agreement with what is widely documented for related diiron and diruthenium complexes.^47,48,55,57−60^

The Cp ligands presumably adopt the *trans* configuration in **5a**,**b**, **6b**, and **7b** (δ = 6.0–6.2 and 5.6–5.8 ppm), in accordance with what was observed in the solid state for **5b** and **7b** (Figures 1 and 2). Conversely, **7a** exists in solution as *cis* and *trans* isomers, with a prevalence of the former (δ = 6.16 and 6.03 ppm, *cis*; δ = 6.01 and 5.83 ppm, *trans*). In general, in analogous diruthenium μ-alkenyl complexes, the *trans* isomer is usually favored over the *cis* isomer, and one Cp resonance is found at higher field in the *trans* isomer than in the *cis* one.^47,48,61^

In **5a** and *trans*-**7a**, the diagnostic resonance for the alkenyl proton bound to carbon α occurs at 11.36 and 10.83 ppm, respectively. Compound **6b** was the only isomer detected as derived from the mixture of **3a** and **3b**: the salient ^1^H NMR feature is represented by two doublets accounting for the =CH_2_ unit, at 5.29 and 3.99 ppm (*J* = 2.6 Hz). The formation of **7a** implies the occurrence of phenyl 1,2-migration during the protonation reaction of **1**; *carbon to carbon* 1,2-migration of the phenyl unit is not unprecedented, and, for instance, it was previously observed on the ruthenium complex [Ru(Cp)(PPh_3_)(=C=C(H)CPh_2_C(R)=C=CH_2_)], undergoing cyclization of the allenyl pendant with the vinylidene group in chloroform at room temperature.^62^

### Behavior of Diruthenium Complexes in Aqueous Solutions

The behavior of the complexes was assessed in aqueous media with a view to biological studies (see Table S1). All complexes displayed a low but appreciable solubility in H_2_O, except **3** and **4**, which were then excluded from the following studies due to the absence of water solubility. The octanol–water partition coefficients (log *P*_ow_) were obtained by a UV–vis spectroscopy method; log *P*_ow_ values of neutral complexes **1**–**2** are ca. 1.3, while log *P*_ow_ values of cationic complexes (**5**–**7**) fall within the range −0.37 to +0.52, indicating an amphiphilic character. The stability of **1**–**2** and **5**–**7** was preliminarily evaluated by UV–vis spectroscopy in the aqueous buffer (NaCac 2.5 mM, at pH = 7.0 and 1% v/v DMSO) at 37 °C, showing negligible decomposition during 3 h. Some additional absorbance tests (signal proportionality at different concentrations and temperature stability range) for the characterization of selected complexes are shown in Figures S12–S14.

The stability of **1**–**2** and **5**–**7** was then estimated by UV–vis spectroscopy in a cell culture medium (DMEM), highlighting the occurrence of a slow degradation process, with approximately 40–60% of each starting compound detected unaltered after 24 h. The representative samples with complexes **1** and **5** were maintained under stirring for a further 3 days and then extracted with dichloromethane. Subsequent IR and ^1^H NMR analyses on the organic phase obtained from **5** revealed the formation of a mixture of neutral complexes comprising the Ru_2_Cp_2_(CO)_3_ core, presumably generated via modification of the alkenyl moiety (shift of the three infrared carbonyl bands to lower wavenumbers; see Experimental for details). This hypothesis is coherent with the versatile chemistry previously documented for diruthenium μ-alkenyl complexes.^63−65^ The produced derivatives might contribute to the cytotoxicity. Instead, the analysis of the organic phase derived from **1** led to identifying **1** as the only residual organometallic species, suggesting that the degradation of **1** occurs with extensive cleavage of the Ru_2_Cp_2_(CO)_3_ structure (CO elimination) without the formation of Ru-CO derivatives. We previously reported that the intracellular, full disassembly of diiron structures based on the Fe_2_Cp_2_(CO)_2_ framework, with the contextual release of carbon monoxide, is key to the cytotoxicity of the complexes.^66,67^ Relevant to this point, it was also demonstrated that stereoisomerism (e.g., *cis*/*trans* geometry of the Cp ligands) has a negligible effect on the anticancer activity of complexes based on the Fe_2_Cp_2_(CO)_2_ skeleton.^68^ We hypothesize that similar considerations are valid for the diruthenium complexes described in the present work; even the recognized geometric isomerism (e.g., **5a** vs **5b**) may not play a significant role, especially in the light of the viability of an interconversion route (see Scheme 2).

### Electrochemistry

**Figure 4:**
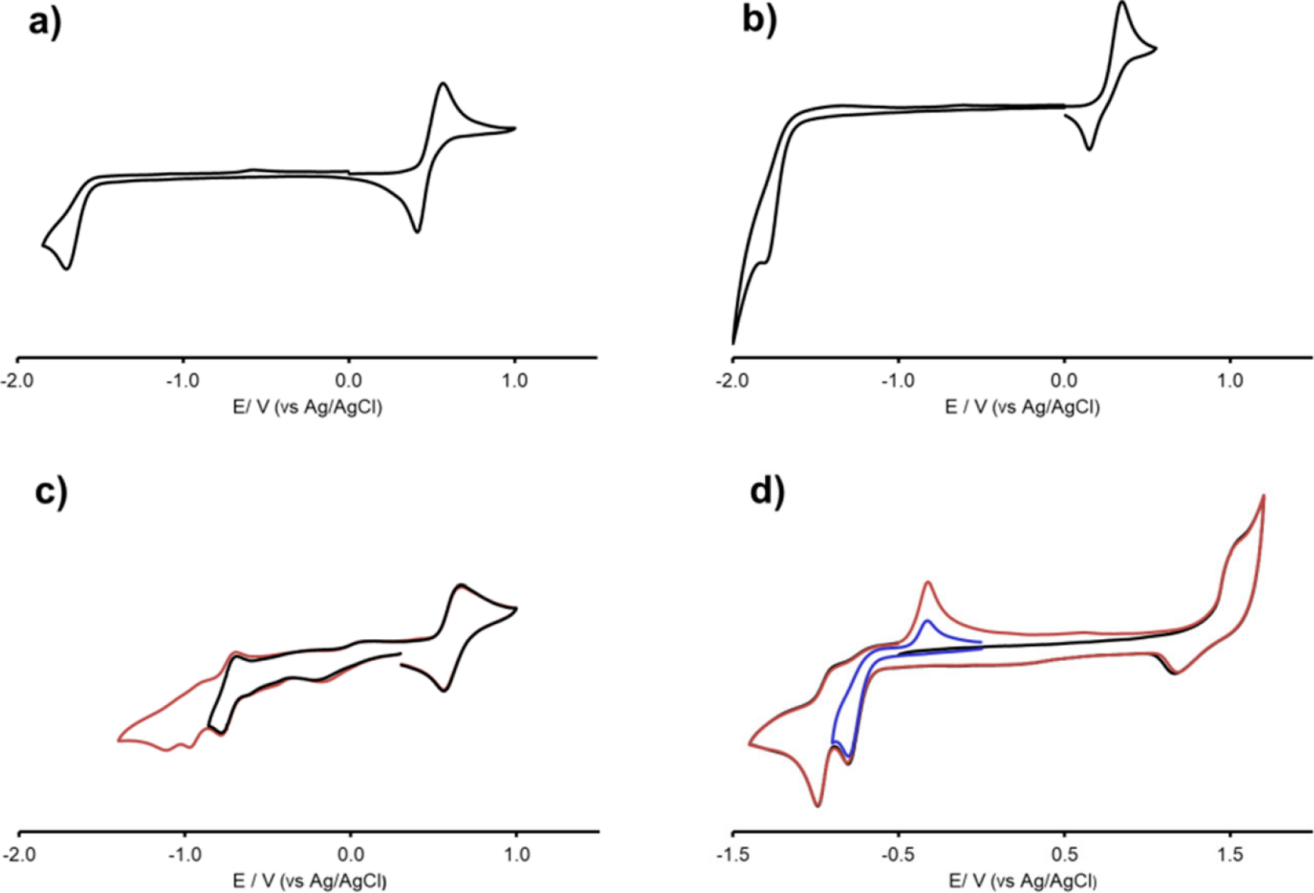
Cyclic voltammograms recorded at a Pt electrode in a CH_2_Cl_2_ solution of (a) **1**; (b) **2**; (c) **5** (red line, CV between +0.95 and −1.35 V; black line, CV between +0.95 and −0.82 V); (d) **7** (first, red line, and second, black line, cycles of two-cycle voltammetry; blue line, CV between 0.0 and −0.9 V). [N*^n^*Bu_4_]PF_6_ (0.2 M) was used as supporting electrolyte. Scan rate: 0.1 V s^–1^.

Electrochemical studies were performed on a selection of diruthenium complexes, i.e., the neutral diruthenacyclopentenones **1** and **2** and the cationic μ-alkenyls **5** and **7**, which were analyzed by cyclic voltammetry in CH_2_Cl_2_/[N^*n*^Bu_4_]PF_6_ solution. The related voltammograms are reported in Figure 4, while Table 2 compiles the formal electrode potentials of the observed redox changes.

**Table 2:** Formal Electrode Potentials^a^ and Peak-to-Peak Separations (mV) for the Redox Changes Exhibited by Diruthenium Complexes in CH_2_Cl_2_/[N^*n*^Bu_4_]PF_6_ 0.2 M

	**Reduction**				**Oxidation**	
	E°′_4_	E°′_3_	E°′_2_	Δ*E*_2_^b^	E°′_1_	Δ*E*_1_^b^
**1**			–1.71^c^ (2.16)		+0.49 (+0.04)	140
**2**			–1.81^c^ (**−**2.26)		+0.24 (−0.21)	241
**5**	–1.11^c^ (−1.56)	–0.97^c^ (−1.42)	–0.73 (−1.18)	92	+0.61 (+0.16)	114
**7**		–1.00^c^ (−1.45)	–0.81^c^ (−1.26)		+1.52^c^ (+1.07)	

a V, vs Ag/AgCl, and, in parentheses, vs FeCp_2_.

b Measured at 0.1 V s^–1^.

c Peak potential value for irreversible processes.

All the complexes exhibit one oxidation, and those of **1**, **2** and **5** can be described as electrochemically quasireversible; as expected, the process occurs at lower potentials in neutral compounds (+0.49 and +0.24 V for **1** and **2**, respectively) compared to values measured on the cationic ones (+0.61 and +1.52 V for **5** and **7**, respectively). The presence of the ferrocenyl moiety in **2** and **5** decreases the oxidation potential in comparison with, respectively, **1** and **7**, which lack the ferrocenyl unit. The shift is very large for the cationic **5** compared to **7**, although it is probably insufficient for enabling the oxidation of **5** in the physiological environment. On the other hand, the ferrocene-based oxidation of **2** occurs at a potential 250 mV lower than that of **1**. Although the bio-oxidative activation of both **1** and **2** appears possible, the cytotoxicity studies suggest that such oxidation is not relevant to the antiproliferative activity of the complexes. Concerning the cathodic region of the CVs, only **5** shows one reduction at −0.73 V with some degree of reversibility, while chemically irreversible processes have been detected for **1**, **2**, and **7**, in the potential range between −0.81 and −1.81 V. Anyway, for all compounds, the activation*in vivo* via reduction appears unlikely.

### Cytotoxicity and Intracellular ROS Generation Studies

The antiproliferative activity of diruthenium complexes (**1**, **2**, **5**, and **7**) was measured on four cancer cell lines (A549, SW480, A2780, A2780cis) and, to outline a possible selectivity, the nontumoral HEK-293 cell line (Table 3). Cisplatin was used as a drug reference.

**Table 3:** IC_50_ Values (μM) Determined for Diruthenium Complexes and Cisplatin on Human Lung Carcinoma (A549), Human Colon Adenocarcinoma (SW480), Human Ovarian Carcinoma (A2780), and Human Ovarian Carcinoma Cisplatin Resistant (A2780cisR) Cancer Cell Lines and a Human Embryonic Kidney (HEK 293) Cell Line after 24 h Incubation^a^

	A549	SW480	A2780	A2780cis	HEK-293
**1**	>100	>100	63 ± 10	>100	>100
**2**	>100	>100	4.1 ± 0.9	4.2 ± 1.1	43 ± 9
**5**	41 ± 5	38 ± 2	8 ± 4	11.0 ± 0.2	13 ± 4
**6**	19 ± 3	22 ± 2	7.9 ± 1.3	9.0 ± 1.3	11.9 ± 1.0
**7**	34 ± 2	34 ± 2	8.5 ± 0.6	10.6 ± 0.8	15 ± 3
cisplatin	43 ± 3	35 ± 2	8.3 ± 1.4	30 ± 3	25.0 ± 1.6

a Values are given as the mean ± SD.

The alkenyl complexes **5**–**7** display promising cytotoxicity against the cancer cell lines, with the related IC_50_ values being close to those obtained with cisplatin, with an absence of appreciable selectivity. A notable exception is given by the ruthenium–aspirin conjugate **6** with reference to the A549 cell line, this complex showing an IC_50_ value which is approximately half that of cisplatin.

On the other hand, the diruthenacyclopentenone complex **1** is substantially inactive, despite its marked lipophilic nature, probably due to the low stability (see Table S1). The absence of activity was also detected for neutral ferrocenyl complex **2** on A549 and SW480 cancer cell lines. Notwithstanding, **2** displays a potent antiproliferative activity against the ovarian cancer cells (A2780 and A2780cis), which is almost 10 times higher than the activity toward the nontumoral HEK-293 cells.

Overall, the activities of **2**, **5**, **6**, and **7** against the cisplatin resistant cell line A2780cis are 3- to 7-fold greater than that exhibited by cisplatin, indicating that these complexes overcome cisplatin resistance in such ovarian cancer cells. Since cisplatin resistance mechanisms in A2780cis cells are related to DNA repair issues and drug efflux,^69^ we may assume that **2**, **5**, **6**, and **7** act through another mechanism of action, that is, either DNA is not their biological target, or, if it is, the mode of interaction and, consequently, the induced DNA damage differs from the type of DNA damage elicited by cisplatin.

**Figure 5:**
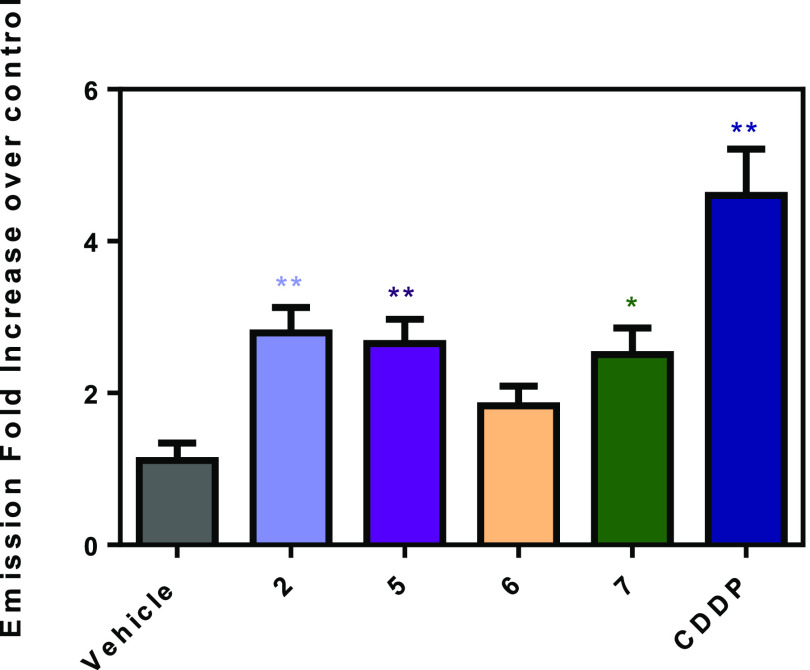
Intracellular ROS levels in A2780 cells incubated with diruthenium complexes at the respective IC_50_ concentrations for 4 h. Cisplatin is included as a reference. Statistical significance, ** *p*-value *<*0.01 and * *p*-value *<*0.05 (ANOVA-Dunnett).

To give insight into the mechanism of action of the compounds, first we studied the ability of **2**, **5**, **6**, and **7** to produce reactive oxygen species (ROS) in A2780 cells by means of the fluorescent probe H_2_DCFDA (2′,7′-dichlorodihydrofluorescein diacetate), which detects hydrogen peroxide (H_2_O_2_) among other radical oxidative species. A comparable ROS generation was observed in A2780 cells treated with **2**, **5**, and **7** at the respective IC_50_ values (Figure 5). This result suggests that the mechanism of ROS production is similar for **2**, **5**, and **7**, and the contribution arising from the oxidation of the ferrocenyl unit^70,71^ (contained in **2** and **5** but not in **7**) is not decisive. By contrast, the observed increase in ROS levels induced by **6**, compared with untreated cells, is not statistically significant; this result might be explained by some interference provided by the aspirin moiety.

### Interaction with Biomolecules

#### Calf-Thymus DNA Binding

The potential interaction of diruthenium complexes with natural DNA (calf-thymus DNA, CT-DNA double-helix type B) was first studied by means of spectrophotometric and spectrofluorimetric microtitrations. In absorption, differential titrations were carried out by adding the same volume of CT-DNA (ca. 2 × 10^–4^ M) to both the cell containing the sample and the reference one, in order to subtract the contribution of the nucleic acid. The binding isotherm was obtained by plotting the signal variation, normalized to the concentration of the metal complex present in the measuring cell, against the CT-DNA content. Figures S15–S17 show the results referred to the **5**/CT-DNA, **6**/CT-DNA, and **7**/CT-DNA systems, respectively, at 25.0 °C. The spectral variation detected upon CT-DNA addition is significant but limited, with neither dramatic changes in the absorbance profile nor isosbestic points. Under these circumstances, a binding mode with a high penetration degree of the metal complex into the polynucleotide helix seems unlikely. The largest spectral change was found for the **6**/CT-DNA system, whereas a weaker effect, hinting at some external binding only, was detected in the case of **7**. To transform these qualitative findings into an evaluation of the binding constant (*K*), HypSpec2014 software was employed to fit the spectral change over the whole range of collected wavelengths. It is known that, in the case of polynucleotides (P), the site dimension (**n**) needs to be considered, **n** being the number of adjacent base pairs which constitute the binding site for the tested molecule (drug/dye, D). In this light, the CT-DNA concentration was uploaded in the software as *C*_P_(sites) = *C*_P_(base pairs)/**n**, using different **n** values searching for the best refinement of the data set. This approach is in line with the original site size definition by Scatchard but neglects the subsequent statistical revisions for sites overlapping discussed by Mc Ghee and Von Hippel.^72^ Nevertheless, in our systems, the approximation is acceptable as the experimental changes are fully reproduced by the software. In all cases, **n** was found to be close to 1. The titrations were repeated at different temperatures, and the resulting *K* values are collected in Table 4. The spectral response upon binding is, at any temperature, fully analogous to that previously reported at 25.0 °C. No binding constant evaluation was possible in the case of **7**/CT-DNA (too low affinity). van’t Hoff plots of the collected *K* values (Figure S18) enable a rough estimation of the thermodynamic parameters for the **5**/CT-DNA and **6**/CT-DNA systems (Table 4), suggesting that the binding is entropically driven. The latter finding agrees with the hypothesis of the absence of deep penetration into the helix.^73^ Note that aspirin and its metabolite salicylate ion were previously reported to bind to the DNA groove.^74^

**Table 4:** Binding Constant Values (*K*) Obtained at Different Temperatures According to the HypSPec2014 Software for the Interaction between CT-DNA and Diruthenium Complexes **5** and **6**^a^

Temperature (°C)	*K* (**5**)	*K* (**6**)
15.0	(1.0 ± 0.2) × 10^5^	(7.8 ± 0.3) × 10 ^4^
25.0	(2.3 ± 0.6) × 10^5^	(1.3 ± 0.6) × 10^5^
37.0	(7.3 ± 0.8) × 10^5^	(1.5 ± 0.3) × 10^5^
50.0	-	(2.6 ± 1.1) × 10^5^
Δ*H* (kJ/mol)	66 ± 1	25 ± 1
Δ*S* (J/K·mol)	326 ± 4	180 ± 1
–*T*Δ*S* (J/mol)	–97 ± 4	–54 ± 1

a The thermodynamic parameters are extracted from the temperature dependence of *K*. NaCac 2.5 mM, pH = 7.0, 1% v/v DMSO.

The interaction with DNA was also investigated by means of metal complex/EB (ethidium bromide) exchange titrations. EB is a fluorescent probe known to emit light at typical wavelengths (λ_exc_ = 520 nm and λ_em_ = 595 nm), only when it is intercalated within the double helix. Tests were performed by inspecting any fluorescence decrease for the EB/CT-DNA mixture upon the addition of increasing amounts of the metal complexes; a blank test was also performed to check the effect of dilution on the signal. Figure S19 shows the results and is in alignment with the considerations above: complex **7** barely interacts, while **5** and **6** induce a large signal decrease, in agreement with strong interaction and EB estrangement.

**Figure 6:**
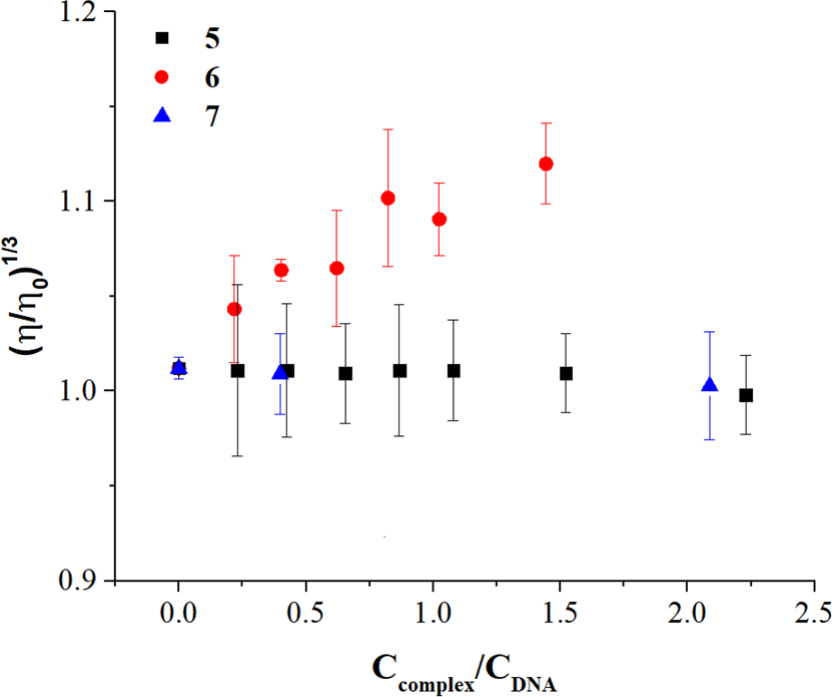
Relative viscosity (η/η°) of CT-DNA as a function of the metal complex content in the mixture; *C*_DNA_ = 9.48 × 10^–5^ M, NaCac 2.5 mM, pH = 7.0, *T* = 25.0 °C. The plot refers to helix elongation defined as (η/η°)^1/3^ = (*t*_mixture_ – *t*_buffer_)/(*t*_DNA_ – *t*_buffer_) with *t* = time of flow in the capillary (s).

Melting tests were also performed to check if the binding could cause some stabilizing or destabilizing effect on the double helix. To this aim, absorbance changes with temperature were recorded over the 25.0–95.0 °C range at 260 nm, affording a sigmoidal plot whose inflection is the melting temperature (*T*_m_) of metal complex/CT-DNA mixture (Figure S20). It turns out that *T*_m_ = 59.5 ± 0.7 °C for **5**/CT-DNA and *T*_m_ = 66.9 ± 0.7 °C for **6**/CT-DNA (*T*_m_ for CT-DNA alone = 57.5 ± 0.4 °C). Keeping in mind the breakdown temperatures for the complexes alone (51 ± 1 °C for **5**, 66 ± 1 °C for **6**, see SI), these numbers will likely be biased by the possible convolution of both the breakdown and melting effects. However, the free metal complex may be calculated to be less than 15%, and one phase only is observed in the plots. On the whole, it seems that **5** does not significantly change the DNA melting temperature, whereas the *T*_m_ of DNA seems to be somewhat increased in the case of the **6**/CT-DNA system. This hints at partial intercalation of the aspirin aromatic fragment between DNA base pairs. Viscosity tests support this hypothesis (Figure 6): the relative viscosity (η/η°) of DNA (*C*_DNA_ = 9.48 × 10^–5^ M) remains constant upon addition (*C*_complex_/*C*_DNA_ from 0 to 2.0) of either **5** or **7** (groove binding only) but increases with **6**, in agreement with some helix elongation.^75^ The enhanced ability of the diruthenium–aspirin conjugate to interact with DNA might explain the better antiproliferative activity exhibited by this complex in A549 and SW480 cells (Table 3).

#### RNA Polynucleotide Binding

The metal complexes were spectrophotometrically titrated by adding increasing amounts of either an RNA double helix (poly(rA)·poly(rU)) or an RNA triple helix (poly(rU)*poly(rA)·poly(rU)). As for CT-DNA, the same RNA amount was added to both measuring and reference cells. Examples of these titrations are provided in the Supporting Information (Figures S21–S26). The same procedure described before was applied to evaluate the metal complex/RNA binding constants; again, **n** = 1 is found to appropriately depict the binding site, and HypSpec2014 was used to calculate the binding constants compiled in Table S2. A major aspect to be highlighted is that **7**, not noticeably interacting with CT-DNA, appears to be suitable to bind poly(rA)·poly(rU). On the other hand, the very high *K* value found in the case of the **7**/poly(rU)*poly(rA)·poly(rU) triplex system suggests the presence of some external cooperative binding only. This selectivity for double-stranded RNA indicates the major role played by the geometrical features of the helix in tuning the presence and absence of affinity in the case of the metal complex bearing the bisphenyl fragment (**7**). Differently, both **5** and **6** display very similar features for binding to RNAs and CT-DNA. The comparable affinity for the triplex suggests that the binding occurs through the minor groove of the duplex, which is not affected by the insertion of the third poly(rU) strand. The binding constants of the **6**/poly(rA)·poly(rU) and **7**/poly(rA)·poly(rU) systems do not noticeably change by lowering the temperature from 25.0 to 15.0 °C, pointing to Δ*H* ≈ 0. For the **5**/poly(rA)·poly(rU) system, *K* becomes negligible at 15.0 °C, indicating Δ*H* > 0, in line with a strong binding. EB/poly(rA)·poly(rU) displacement tests (Figure S27) confirm this view: complex **7** shows some interaction with RNA, similarly to **6**, while **5** strongly affects the light emission properties of the probe. Melting tests (Figure S28) evidence some destabilization of poly(rA)·poly(rU) and poly(rU)*poly(rA)·poly(rU), according to the absence of helix penetration (no superimposition with metal complex breakdown is likely to occur here).

#### Bovine Serum Albumin (BSA) Binding

**Figure 7:**
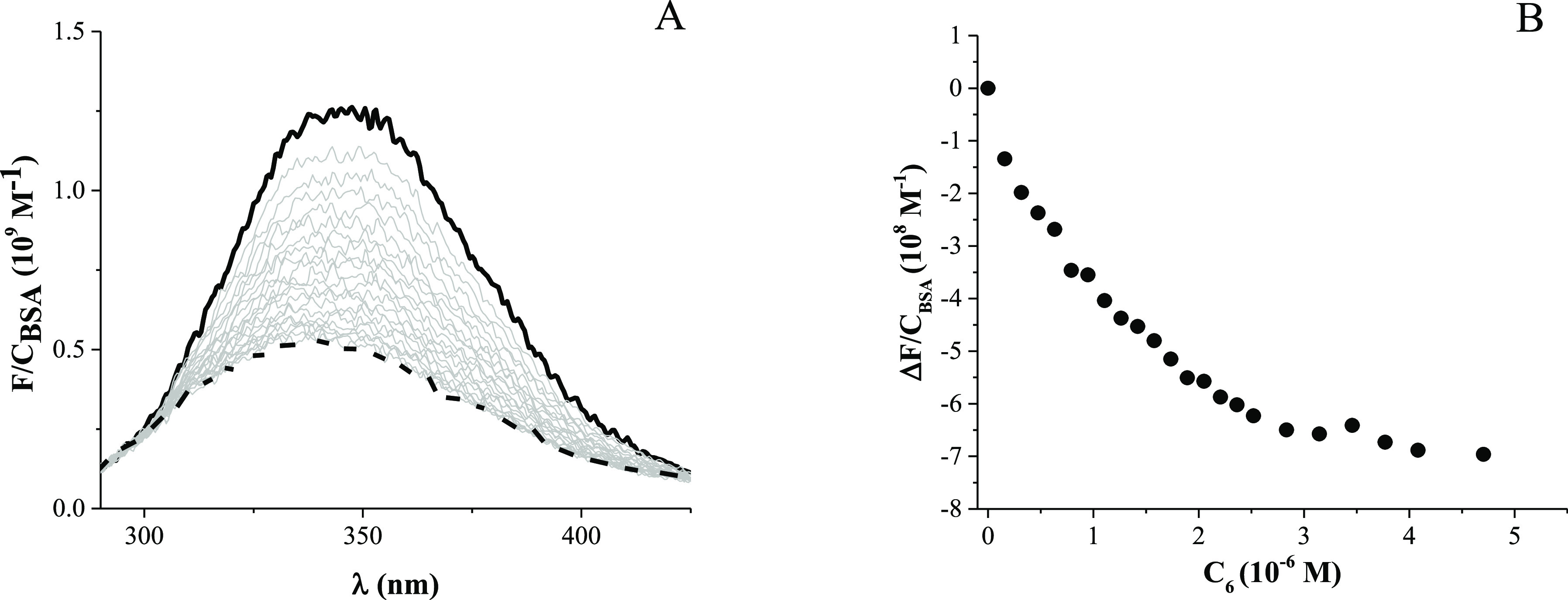
Fluorescence spectra (A) and binding isotherm (B) at λ_em_ = 340 nm for the **6**/BSA system: *C*_BSA_ = 5.11 × 10^–7^ M, *C*_6_ = 0 M (solid line) to 4.70 × 10^–6^ M (dashed line); λ_exc_ = 280 nm, NaCac 2.5 mM, pH = 7.0, *T* = 25.0 °C.

The possible interaction of the metal complexes with BSA was investigated by fluorescence measurements (at 25.0 and 37.0 °C) by adding known volumes of the species directly into the cell containing the light-emitting BSA solution (approximately 5 × 10^–7^ M). The DMSO content in the titrant may alter the BSA fluorescence properties; thus, we added only small aliquots of solutions of the metal complexes (approximately 10^–4^ M, 10% v/v DMSO) to the BSA solution in the cell so that in the whole titration the DMSO concentration did not exceed 2% v/v. Also, blank tests (addition of solvent only) were performed and demonstrated that, in the absence of a metal complex, the BSA signal changed less than 5%. On the other hand, Figures 7 and S29–S30 show that **5**, **6**, and **7** induce a dramatic quenching of fluorescence.

Data were interpolated by means of the modified Stern–Volmer equation (see SI and Table S3) also to control that signal decrease is not exclusively due to collisional effects. The Stern–Volmer constants do not change noticeably with temperature and are much higher than 1000, confirming that a binding between each metal complex and the protein is indeed at play.^76^ Then, the experimental data were interpolated through the Hypspec2014 software; a 1:1 reaction stoichiometry was found to be appropriate to depict the experimental observation. Table S3 also shows the binding constant values (*K*_BSA_) obtained with this procedure. The affinity is, in all cases, remarkable: *K*_BSA_ is on the magnitude order of 10^7^ for **5** and **7** and 10^6^ for **6**. Recent studies by some of us confirm the hypothesis that there is an optimal window for drug transportation by albumin protein, and in particular, a too strong binding reduces the antitumor activity by preventing the compound from reaching the target.^77,78^ In this frame, aspirin derivative **6** displays the best *K*_BSA_ value. For all three metal complexes, *K*_BSA_ increases with temperature, indicating an uptake driven by hydrophobic forces. The absence of evidence for a possible strong, covalent binding between the complexes and proteins suggests that the activity of the compounds is mainly exerted inside the cells, rather than involving extracellular targets as previously highlighted for lead ruthenium drug candidates.^79^ This hypothesis validates the possibility that the binding of the investigated diruthenium complexes with nucleotides might play some role in their antiproliferative activity.

## Concluding Remarks

Organometallic ruthenium complexes hold much promise in view of anticancer chemotherapeutic treatment, and to date, studies have been focused on mononuclear ruthenium–arene complexes. We report herein a synthetic strategy to access new diruthenium complexes based on the Ru_2_Cp_2_(CO)_3_ scaffold with a variable bridging hydrocarbyl ligand carrying different functions, the structural characterization, and an evaluation of the anticancer potential. In general, the investigated diruthenium complexes display a promising antiproliferative activity against different cancer cell lines, which is comparable and in some cases even stronger than that of cisplatin. Moreover, most of the diruthenium complexes can circumvent cisplatin resistance in ovarian cancer cells (A2780cis). Experiments reveal that the mechanism of action of the compounds could be multimodal, including the enhancement of ROS generation and the binding with DNA or RNA (depending on the cases) and possibly ascribable, at least in part, to derivatives formed via modification of the hydrocarbyl ligand. Albumin protein is a potential vehicle for the transportation and delivery of the studied complexes through the establishment of hydrophobic interactions. Remarkably, the choice of the hydrocarbyl ligand substituents modulates the performance of the complexes, in terms of effectiveness toward specific cancer cell lines, ROS production, and binding with polynucleotides. For instance, the introduction of the aspirin fragment suppresses ROS generation but favors DNA binding through a half-intercalation mode. Considering that the synthesis reaction is general and may take advantage of the wide availability of commercial alkynes (and their possible derivatives) to incorporate various functional groups, the proposed family of compounds serves as a promising basis with a view to drug development. Indeed, a future, extensive structure/activity relationship exploration may lead to identifying targeted drug candidates with optimal characteristics, in terms of both physicochemical properties and biological activity.

## Experimental Section

### Synthesis and Structural Characterization of Diruthenium Complexes

#### General Details

Reactants and solvents were purchased from Alfa Aesar, Merck, Strem, or TCI Chemicals and were of the highest purity available. Diruthenacyclopentenone complex **1**(36) and 3-ethynylphenyl 2-acetoxybenzoate^38,39^ were prepared according to the literature. Reactions were conducted under a N_2_ atmosphere using standard Schlenk techniques. Products were stored in air once isolated. Dichloromethane and tetrahydrofuran were dried with the solvent purification system mBraun MB SPS5, while acetonitrile was distilled from CaH_2_. IR spectra of solutions were recorded by using a CaF_2_ liquid transmission cell (2300–1500 cm^–1^) on a PerkinElmer Spectrum 100 FT-IR spectrometer. IR spectra were processed with Spectragryph software.^80^^1^H and ^13^C NMR spectra were recorded on a Jeol JNM-ECZ500R instrument equipped with a Royal HFX Broadband probe at 298 K, unless otherwise specified. Chemical shifts (expressed in parts per million) are referenced to the residual solvent peaks.^81^ NMR spectra were assigned with the assistance of ^1^H–^13^C (*gs*-HSQC and *gs*-HMBC) correlation experiments.^82^ NMR signals due to secondary isomeric forms (where it is possible to detect them) are italicized. Elemental analyses were performed on a Vario MICRO cube instrument (Elementar).

### Synthesis of Diruthenacyclopentenone Complexes

#### General Procedure

Complex [Ru_2_Cp_2_(CO)(μ-CO){μ-η^1^:η^3^-C(O)C(Ph)C(Ph)}], **1** (60 mg, 0.10 mmol), the selected alkyne (3–5 equiv), and toluene (30 mL) were placed in a 100 mL round-bottom flask. The mixture was stirred at reflux temperature for 1–4 h, and the consumption of **1** was checked by IR spectroscopy. Volatiles were evaporated under reduced pressure, the solid residue was dissolved in Et_2_O/CH_2_Cl_2_ (5:1 v/v), and this solution was charged on an alumina column. Neat diethyl ether allowed to elute impurities, while the band corresponding to the desired product was collected using THF. After solvent removal, an oily residue was obtained. Dissolution in dichloromethane (7 mL) and addition of petroleum ether (60 mL) to the solution afforded a powder, which was isolated, washed with Et_2_O (3 × 10 mL), and finally dried under vacuum.

#### [Ru_2_Cp_2_(CO)(μ-CO){μ-η^1^:η^3^-CH=C(Fc)C(=O)}], **2a**

**Figure 8:**
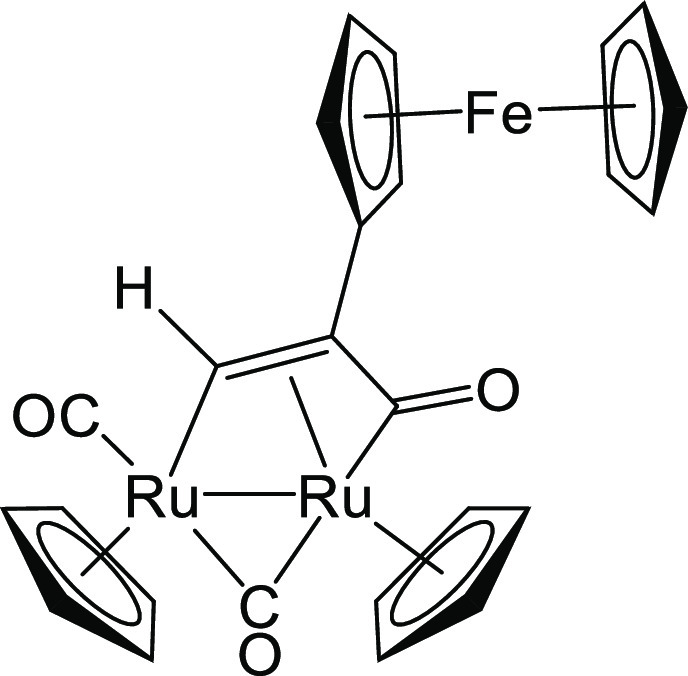
Structure of **2a**.

From **1** (60 mg, 0.10 mmol) and 1-ethynylferrocene (85 mg, 0.40 mmol) (Figure 8). Reaction time: 1 h. Brown solid, yield 58 mg (92%). Anal. Calcd for C_25_H_20_FeO_3_Ru_2_: C, 47.93; H, 3.22. Found: C, 47.70; H, 3.25. IR (CH_2_Cl_2_): ν̃/cm^–1^ = 1972vs (CO), 1800s (μ-CO), 1748w-br (C=O). ^1^H NMR (acetone-*d*_6_): δ 10.93 (s, 1 H, CH=); 5.60, 5.04 (s, 10 H, Cp); 5.74, 4.25, 4.15, 4.11 (m, 4 H, C_5_H_4_); 4.16 (s, 5 H, Cp^Fc^). ^13^C{^1^H} NMR (acetone-*d*_6_): δ 236.6 (μ-CO); 220.9 (C=O); 201.6 (CO); 150.2 (CH=); 90.4, 88.9 (Cp); 85.0 (*ipso*-C_5_H_4_); 70.0 (Cp^Fc^); 69.6, 69.4, 68.2, 67.0 (C_5_H_4_); 44.4 (CC_5_H_4_). ^1^H NMR (toluene-*d*_8_, 298 K): δ/ppm = 10.61 (s, 1 H, CH=); 4.76, 4.57 (s, 10 H, Cp); 4.65, 4.26, 4.11, 3.99 (m, 4 H, C_5_H_4_); 4.24 (s, 5 H, Cp^Fc^).

#### [Ru_2_Cp_2_(CO)(μ-CO){μ-η^1^:η^3^-CH=C(3-C_6_H_4_-Asp)C(=O)}], **3a**, and [Ru_2_Cp_2_(CO)(μ-CO){μ-η^1^:η^3^-C(3-C_6_H_4_-Asp)=CHC(=O)}], **3b**

**Figure 9:**
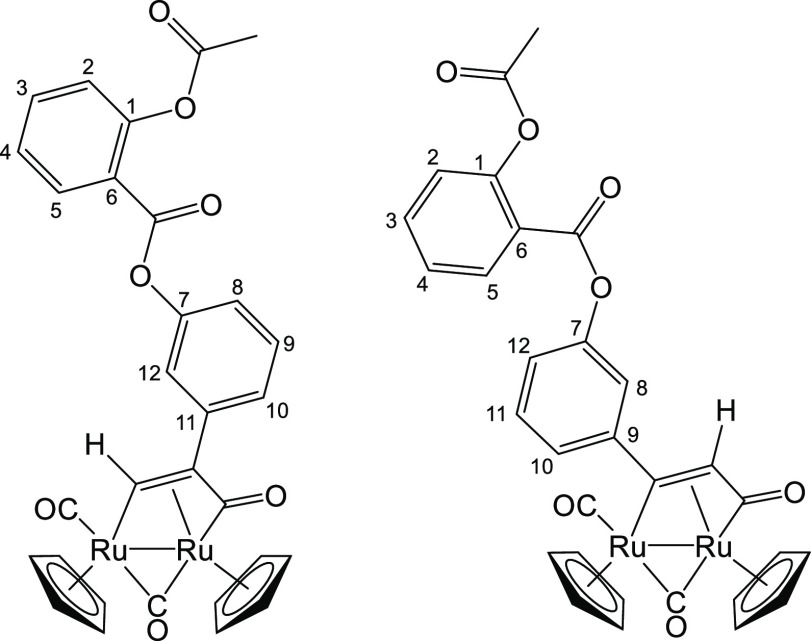
Structures of **3a** (left) and **3b** (right).

From **1** (60 mg, 0.10 mmol) and 3-ethynylphenyl 2-acetoxybenzoate (113 mg, 0.404 mmol) (Figure 9). Reaction time: 4 h. Brown solid, 39 mg (55%). Anal. Calcd for C_30_H_22_O_7_Ru_2_: C, 51.72; H, 3.18. Found: C, 51.54; H, 3.13. IR (CH_2_Cl_2_): ν̃/cm^–1^ = 1980vs (CO), 1805s (μ-CO), 1769w, 1744m-s (C*=*O).

**3a**. ^1^H NMR (CDCl_3_): δ 11.11 (s, 1 H, CH=); 8.25, 7.67, 7.51, 7.44–7.32, 7.19, 7.02 (m, 8 H, arom); 5.46, 5.06 (s, 10 H, Cp); 2.31 (s, 3 H, Me). ^13^C{^1^H} NMR (CDCl_3_): δ/ppm = 235.8 (μ-CO); 221.2 (C=O); 198.2 (CO); 170.0 (MeC=O); 163.1 (C_6_H_4_C=O); 140.9 (C^11^); 151.5, 150.9 (C^1^ + C^7^); 149.9 (CH=); 134.9, 132.4, 129.8, 126.4, 125.1, 124.2, 121.0, 120.5 (C^2–5^ + C^8–10^ + C^12^); 122.6 (C^6^); 89.6, 88.7 (Cp); 43.1 (CH=C); 21.2 (Me).

**3b**. ^1^H NMR (CDCl_3_): δ 5.30, 5.21 (s, 10 H, Cp); 3.37 (s, 1 H, CH=); 2.29 (s, 3 H, Me). ^13^C{^1^H} NMR (CDCl_3_): δ/ppm = 235.4 (μ-CO); 220.8 (C=O); 198.6 (CO); 179.9 (CH=C); 169.9 (MeC=O); 163.4 (C_6_H_4_C=O); 156.8 (C^11^); 151.3, 151.1 (C^1^ + C^7^); 135.0, 132.3, 129.3, 124.8, 124.0, 119.0 (C^2–5^ + C^8–10^ + C^12^); 122.6 (C^6^); 91.2, 88.8 (Cp); 26.9 (CH=); 21.4 (Me). Aromatic signals for **3b** are partially hidden by the signals of **3a**. Ratio **3a**/**3b** = 3.

#### [Ru_2_Cp_2_(CO)(μ-CO){μ-η^1^:η^3^-CH=C(2-naphthyl)C(=O)}], **4a**, and [Ru_2_Cp_2_(CO)(μ-CO){μ-η^1^:η^3^-C(2-naphthyl)=CHC(=O)}], **4b**

**Figure 10:**
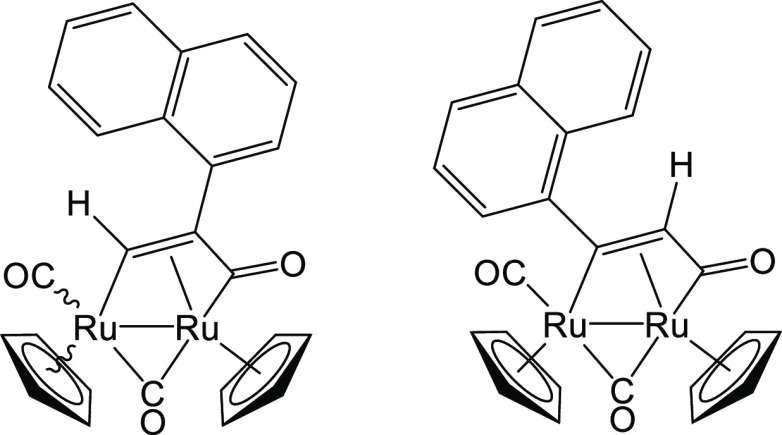
Structures of **4a** (left) and **4b** (right).

From **1** (60 mg, 0.10 mmol) and 1-ethynylnaphthalene (0.06 mL, 0.42 mmol) (Figure 10). Reaction time: 3 h. Dark-brown solid, yield 26 mg (45%). Anal. Calcd for C_25_H_18_O_3_Ru_2_: C, 52.81; H, 3.19. Found: C, 53.02; H, 3.16. IR (CH_2_Cl_2_): ν̃/cm^–1^ = 1977vs (CO), 1803s (μ-CO), 1751w (C=O).

**4a** (*cis* + *trans*). ^1^H NMR (CDCl_3_): δ (ppm) = *11.29*, 10.74 (s, 1 H, CH=); 8.34–8.22, 7.91–7.88, 7.79–7.75, 7.72, 7.54–7.51, 7.40 (m, 7 H, arom); *5.32*, 5.27, *5.02*, 5.01 (s, 10 H, Cp). ^13^C{^1^H} NMR (CDCl_3_): δ/ppm = 151.8 (CH=); 133.6–125.0 (arom); 89.5, 88.6 (Cp); 53.5 (CH=C).

**4b**. ^1^H NMR (CDCl_3_): δ 8.34–8.30, 7.91–7.88, 7.55–7.52, 7.41–7.38 (m, 7 H, Ph); 5.38, 4.96 (s, 10 H, Cp); 3.48 (s, 1 H, CH=). ^13^C{^1^H} NMR (CDCl_3_): δ 235.3 (μ-CO); 221.1 (C=O); 199.2 (CO); 182.6 (CH=C); 159.4 (*ipso***-**C_10_H_7_); 133.6–125.0 (C_10_H_7_); 34.4 (CH=). Naphthyl signals are almost superimposed in **4a** and **4b**. Ratio **4b**/*cis*-**4a**/*trans*-**4a** = 4:2:1.

### Synthesis of Cationic μ-Alkenyl Complexes

#### General Procedure

Complexes **1**–**3** (0.050 mmol) were dissolved in CH_2_Cl_2_ (20 mL) and treated with 1.2 equiv of HBF_4_·Et_2_O (ca. 0.01 mL) under a N_2_ atmosphere. The solution was stirred for 15 min, then H_2_O (3 mL) was added, and the mixture was stirred for further 15 min. The organic phase was separated from the aqueous phase, and then, the latter was extracted with dichloromethane (3 × 15 mL). The organics were collected and then concentrated to 5 mL by evaporation at reduced pressure. Afterward, Et_2_O (50 mL) was added, causing the precipitation of the products **5**–**7** as powdery solids that were isolated and dried under vacuum.

#### [Ru_2_Cp_2_(CO)_2_(μ-CO){μ-η^1^:η^2^-CH=CH(Fc)}]BF_4_, **5a**, and [Ru_2_Cp_2_(CO)_2_(μ-CO){μ-η^1^:η^2^-C(Fc)CH_2_}]BF_4_, **5b**

**Figure 11:**
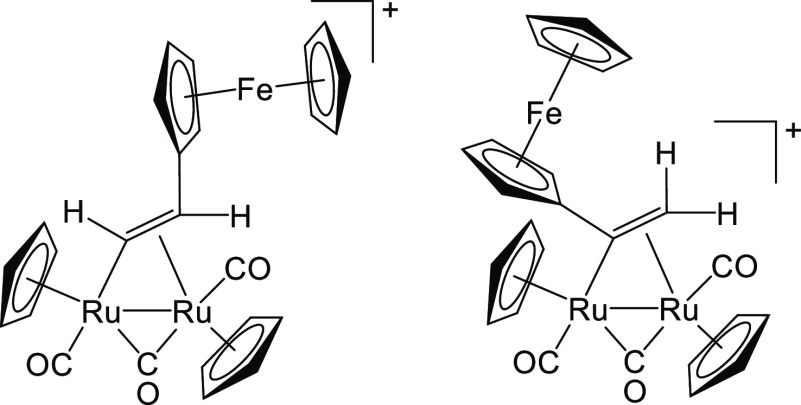
Structures of the cations of **5a** (left) and **5b** (right).

From **2b** (31 mg, 0.050 mmol) (Figure 11). Dark-brown solid, yield 84 mg (40%). Anal. Calcd for C_25_H_21_BF_4_FeO_3_Ru_2_: C, 42.04; H, 2.96. Found: C, 42.12; H, 3.05. IR (CH_2_Cl_2_): ν̃/cm^–1^ = 2035vs (CO), 2015vs (CO), 1885m (μ-CO).

**5a**. ^1^H NMR (acetone-*d*_6_, 183 K): δ/ppm = 11.36 (d, 1 H,^3^*J*_HH_ = 10.8 Hz, RuCH=); 6.2, 5.5* (s, 10 H, Cp); 6.28, 6.19, 4.81–4.60 (m, 4 H, C_5_H_4_); 4.38 (s, 5 H, Cp^Fc^); 3.31 (m, 1 H, =CH).

**5b**. ^1^H NMR (acetone-*d*_6_, 183 K): δ 6.39, 5.79–5.70, 5.54 (m, 4 H, C_5_H_4_); 6.04, 5.58 (s, 10 H, Cp); 4.44 (m, 2 H, =CH_2_); 4.28 (s, 5 H, Cp^Fc^). *Cp signals of two isomers are overlapped. Ratio **5b**/**5a** = 1.7. Crystals of **5b** suitable for X-ray analysis were collected by slow diffusion of hexane into a dichloromethane solution at −30 °C.

#### [Ru_2_Cp_2_(CO)_2_(μ-CO){μ-η^1^:η^2^-C(3-C_6_H_4_-Asp)=CH_2_}]BF_4_, **6b**

**Figure 12:**
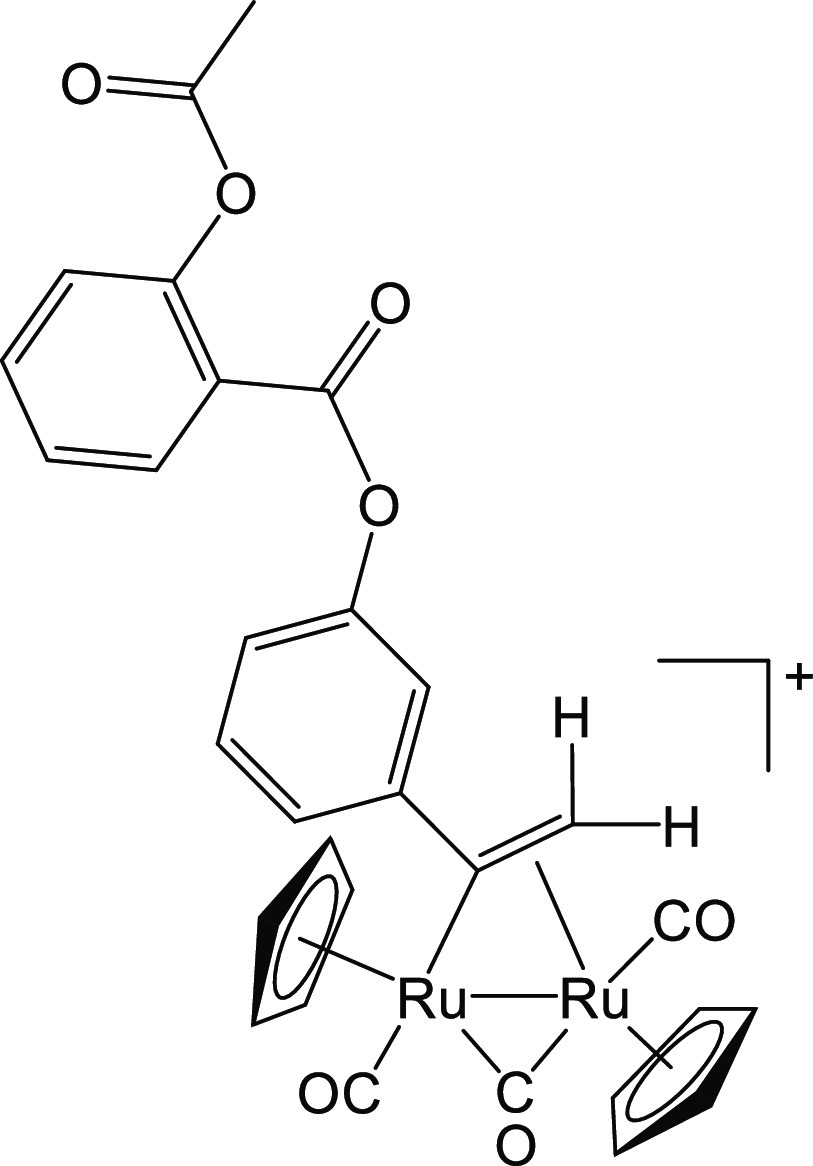
Structure of cation of **6b**.

From **3a**–**b** (35 mg, 0.050 mmol) (Figure 12). Light-brown solid, 25 mg (64%). Anal. Calcd for C_30_H_23_BF_4_O_7_Ru_2_: C, 45.93; H, 2.96. Found: C, 45.77; H, 3.09. IR (CH_2_Cl_2_): ν̃/cm^–1^ = 2036s (CO), 2018vs (CO), 1874m (μ-CO), 1762s, 1743vs (C*=*O). ^1^H NMR (acetone-*d*_6_, 223 K): δ 8.25, 7.85–7.81, 7.65, 7.56–7.48, 7.36, 7.29, 7.12–7.05 (m, 8 H, arom); 5.98, 5.86 (s, 10 H, Cp); 5.29, 3.99 (d,^3^*J*_HH_ = 2.6 Hz, 2 H, =CH_2_); 2.20 (s, 3 H, Me).

#### [Ru_2_Cp_2_(CO)_2_(μ-CO){μ-η^1^:η^2^-C(H)CPh_2_}]BF_4_, **7a**, and [Ru_2_Cp_2_(CO)_2_(μ-CO){μ-η^1^:η^2^-C(Ph)CH(Ph)}]BF_4_, **7b**

**Figure 13:**
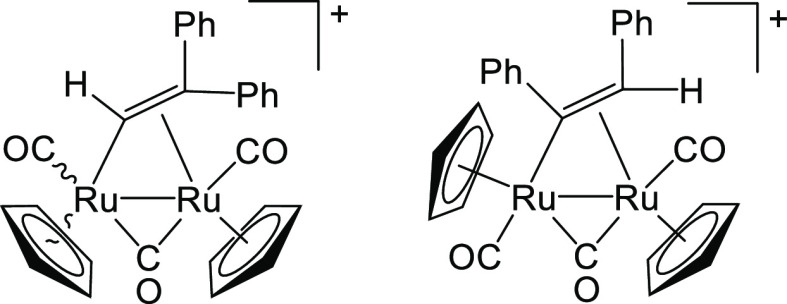
Structures of the cations of **7a** (left) and **7b** (right).

From **1** (280 mg, 0.471 mmol) (Figure 13). Yellow solid, yield 276 mg (86%). Anal. Calcd for C_27_H_21_BF_4_O_3_Ru_2_: C, 47.52; H, 3.10. Found: C, 45.69; H, 3.02. IR (CH_2_Cl_2_): ν̃/cm^–1^ = 2035vs (CO), 2016vs (CO), 1871m (μ-CO).

**7a**. ^1^H NMR (acetone-*d*_6_, 193 K): δ 10.83, *9.88* (m, 1 H, CH=); 7.45–7.15 (m, 10 H, Ph); 6.16, *6.01*, 6.03, *5.83* (s, 10 H, Cp).

**7b**. ^1^H NMR (acetone-*d*_6_, 193 K): δ (ppm) = 7.47–7.44, 7.33, 7.28–7.25, 7.18 (m, 10 H, Ph); 6.04, 5.82 (s, 10 H, Cp); 5.55 (s, 1 H, CH=).

Ratio **7b**/*cis*-**7a**/*trans*-**7a** = 1:0.8:0.15.

Crystals of **7b** suitable for X-ray analysis were collected by the slow diffusion of pentane into a dichloromethane solution at room temperature.

### X-ray Crystallography

Crystal data and collection details for **5b** and **7b** are reported in Table 5. Data were recorded on a Bruker APEX II diffractometer equipped with a PHOTON2 detector using Mo–Kα radiation. The structures were solved by direct methods and refined by full-matrix least-squares based on all data using *F*^2^.^83^ Hydrogen atoms were fixed at calculated positions and refined by using a riding model.

**Table 5:** Crystal Data and Measurement Details for **5b** and **7b**

	**5b**	**7b**
Formula	C_25_H_21_BF_4_FeO_3_Ru_2_	C_27_H_21_BF_4_O_3_Ru_2_
FW	714.22	682.39
*T*, K	100(2)	100(2)
λ, Å	0.71073	0.71073
Crystal system	Orthorhombic	Monoclinic
Space group	*P*2_1_2_1_2_1_	*P*2_1_/*n*
*a*, Å	7.9280(4)	8.6881(3)
*b,* Å	9.9718(5)	14.9259(5)
*c*, Å	59.452(3)	19.1832(7)
β, °	90	101.9490(10)
Cell volume, Å^3^	4700.0(4)	2433.73(15)
Z	8	4
*D*_c_, g·cm^–3^	2.019	1.862
μ, mm^–1^	1.937	1.301
*F*(000)	2800	1344
Crystal size, mm	0.18 × 0.14 × 0.11	0.18 × 0.16 × 0.13
θ limits, °	2.071–25.097	1.743–25.993
Reflections collected	60381	33 776
Independent reflections	8379 [*R*_int_ = 0.375]	4778 [*R*_int_ = 0.320]
Data/restraints/parameters	8379/557/663	4778/10/371
Goodness on fit on *F*^2^^a^	1.363	1.094
*R*_1_ (*I* > 2σ(*I*))^b^	0.0395	0.0250
*wR*_2_ (all data)^c^	0.0857	0.0636
Largest diff. peak and hole, e Å^–3^	1.296/–1.000	1.385/–0.515

a Goodness on fit on *F*^2^ = [Σ*w*(*F*_O_^2^ – *F*_C_^2^)^2^/(*N*_ref_ – *N*_param_)]^1/2^, where *w* = 1/[σ^2^(*F*_O_^2^) + (*aP*)^2^ + *bP*], where *P* = (*F*_O_^2^ + 2*F*_C_^2^)/3; *N*_ref_ = number of reflections used in the refinement; *N*_param_ = number of refined parameters.

b *R*_1_ = Σ||*F*_O_| – |*F*_C_||/Σ|*F*_O_|.

c *wR*_2_ = [Σ*w*(*F*_O_^2^ – *F*_C_^2^)^2^/Σ*w*(*F*_O_^2^)^2^]^1/2^, where *w* = 1/[σ^2^(*F*_O_^2^) + (*aP*)^2^ + *bP*], where *P* = (*F*_O_^2^ + 2*F*_C_^2^)/3.

### Behavior in Aqueous Solutions (Table S1)

#### Determination of Partition Coefficients (Log *P*_ow_)

Partition coefficients (log *P*_ow_), defined as *P*_ow_ = *c*_org_/*c*_aq_, where *c*_org_ and *c*_aq_ are the molar concentrations of the selected compound in the *n*-octanol and aqueous phases, respectively, were determined by the shake-flask method and UV–vis measurements, according to a previously described procedure.^84^ All the operations were carried out at 21 ± 1 °C. The wavelength of the maximum absorption of each compound in the 270–350 nm range was used for UV–vis quantitation.

#### Stability in Buffer (NaCac 2.5 mM, pH = 7.0, 1% v/v DMSO)

The UV–vis absorbance spectrum % variation of the metal complexes was measured over a time lapse of 3 h: in all cases, signal variation was <2% at 25.0 °C and ≤10% at 37.0 °C.

#### Stability in Cell Culture Medium (DMEM) Solution

Solutions (ca. 10^–5^ M) of diruthenium complexes in a mixture of DMSO and DMEM (ca. 1:4 v/v) were analyzed by UV–vis spectroscopy immediately after the preparation of the samples (*t*_0_) and after being stored for 24 h at room temperature. The % of residual complex in solution was calculated based on the absorbance variation at a maximum wavelength. The mixtures derived from **1** and **5** were maintained under stirring for further 72 h, and then, they were extracted twice with dichloromethane. The aqueous phases appeared pale-colored, while the yellow-brown organic phases were collected, concentrated, and analyzed by IR spectroscopy (CH_2_Cl_2_) solution. The IR spectrum of the sample derived from **1** revealed the presence of **1** as a unique carbonyl species. The IR pattern of the sample derived from **5** matches that of **5**, with the set of three carbonyl bands shifted to lower wavenumbers [IR (CH_2_Cl_2_): ν̃/cm^–1^ = 1985vs (CO), 1956s-sh (CO), 1792m (μ-CO)]; ^1^H NMR analysis (CDCl_3_ solution) pointed out the presence of a complicated mixture of Cp-containing complexes [δ/ppm = 10.74 (d, *J* = 13 Hz), 10.25 (s), 9.87 (d, *J* = 13 Hz), 9.54 (s), 8.96 (*J* = 13.5 Hz), 8.67 (s), 7.53 (m), 8.00 (m), 6.88 (m), 6.54 (m), 6.29 (m), 5.27, 5.25, 5.23, 5.20 (s, Cp), 5.05 (s), 4.95 (s), 4.84 (m), 4.25 (d), 4.22 (s), 0.87 (t)].

### Electrochemistry

Cyclic voltammetry measurements were performed with a PalmSens4 instrument interfaced to a computer employing PSTrace5 electrochemical software. Anhydrous CH_2_Cl_2_ (Merck) was stored under Ar over 3 Å molecular sieves. [N^*n*^Bu_4_]PF_6_ (Fluka, electrochemical grade) and FeCp_2_ (Fluka) were used without further purification. CV measurements were carried out under Ar using 0.2 M [N^*n*^Bu_4_]PF_6_ in CH_2_Cl_2_ as the supporting electrolyte. The working and counter electrodes consisted of a Pt disk and a Pt gauze, respectively. A leakless miniature Ag/AgCl/KCl electrode (eDAQ) was employed as a reference. The three-electrode home-built cell was predried by heating under vacuum and filled with argon. The Schlenk-type construction of the cell maintained anhydrous and anaerobic conditions. The solution of supporting electrolyte, prepared under argon, was introduced into the cell, and the CV of the solvent was recorded. The analyte was then introduced and voltammograms were recorded; last, a small amount of ferrocene was added, and the CV was repeated. Under the present experimental conditions, the one-electron oxidation of ferrocene occurred at *E*° = +0.45 V vs Ag/AgCl, KCl sat.

### Cellular Experiments

Human lung adenocarcinoma (A549), colon adenocarcinoma (SW480), ovarian cancer (A2780) cisplatin resistance ovarian cancer (A2780cis), and embryonic kidney cells (HEK-293) were obtained from the European Collection of Cell Cultures (EACC). A549, SW480, A2780, and A2780cis cells were cultured in DMEM (Dulbecco’s Modified Eagle Medium) and HEK293 cells in MEM (Minimum Essential Medium Eagle) supplemented with 2 mM of glutamine and 1% of nonessential amino acids (NEAA). Both media were supplemented with 10% fetal bovine serum (FBS) and 1% amphotericin-penicillin-streptomycin solution. An MTT (3-(4,5-dimethyltiazol-2-yl)-2,5-diphenyltetrazoliumbromide) assay was performed as previously described.^85^ Briefly, cells were seeded in 96-well plates at a density of 5 × 10^3^ (A549), 1 × 10^4^ (SW480), or 2 × 10^4^ A2780, A2780cis, and HEK293) cells per well. After 24 h, cells were treated with different concentrations of the Ru complexes. A vehicle control with DMSO at the maximal employed concentration (0.5%) was also included as well as cisplatin (CDDP) as a positive control. After 24 h of incubation, treatment was removed, and the MTT solution (500 μg/mL) was added. After 3 h of incubation, the formazan crystals were dissolved, and absorbance was read at 590 nm in a microplate reader (Cytation 5 Cell Imaging Multi-Mode Reader, Biotek Instruments, USA). Two independent experiments were performed with four replicates per dose. The IC_50_ values were calculated using GraphPadPrism Software Inc. (ver. 6.01) (USA). For intracellular ROS levels quantification,^86^ A2780 cells were seeded in a clear bottom black side 96 well plate (Costar) at a density of 5 × 10^4^ cells per well and incubated for 24 h. Then, 100 μL of 25 μM 2′,7′-dichlorofluorescein diacetate (H_2_DCFDA) was added to each well. After 30 min of incubation with the probe, cells were treated with the appropriate concentration of the complexes equal to the IC_50_ value (previously calculated by the MTT assay); 10 μM TBH and CDDP were included as positive controls. After 4 h of treatment, cells were washed twice with DPBS, and emission was measured at λem = 530 nm with λexc = 490 nm in a microplate reader (Cytation 5 Cell Imaging Multi-Mode Reader, Biotek Instruments, USA). Two independent experiments with four replicates per treatment were performed.

Statistical analysis of data from cellular assays was performed by GraphPad Prism 6 software. All data were expressed as the mean with standard deviation. The level of significance between different treatments relative to control was estimated by ANOVA with Dunnet’s Test. A *p*-value <0.05 was considered statistically significant.

### Interaction with Biomolecules

#### Materials

Stock solutions of the diruthenium complexes (approximately 2 × 10^–3^ M) were obtained by dissolving known quantities of the solid in DMSO (puriss. p.a., Merck). All solutions were stored at 4 °C and changed frequently. CT-DNA (calf-thymus DNA) was supplied by Merck as a lyophilized sodium salt and solubilized in ultrapure water. The stock solutions were subjected to sonication procedures to polynucleotides of about 500 base pairs long.^87^ The concentration (in molarity of base pairs) of the stock solutions (approximately 2.5 × 10^–3^ M) was evaluated by UV–vis absorption (NaCac 2.5 mM, pH = 7.0, λ = 260 nm, ε = 13 200 M^–1^ cm^–1^.^88^ NaCac is sodium cacodylate (dimethylarsinic acid sodium salt), from Merck (BioXtra, ≥98%). The synthetic RNA used was formed by the union of polyriboadenylic acid (poly(rA)) and polyribouracil (poly(rU)) single strands, from Merck, according to a known procedure.^89^ Briefly, each polynucleotide was dissolved in a 2.5 mM NaCac pH = 7.0 buffer, and the concentration (in bases) was measured by UV–vis spectroscopy considering ε(257 nm) = 10 110 cm^–1^ M^–1^ for poly(rA) and ε(257 nm) = 8900 cm^–1^ M^–1^ for poly(rU). For the formation of the poly(rA)·poly(rU) double helix, the solutions were mixed in a 1:1 ratio and left to rest overnight at room temperature in the dark. The concentration of the mother solution obtained, expressed in base pairs, is approximately 8.4 × 10^–4^ M. For the formation of the triple helix, the process is similar: to the already synthesized poly(rA)·poly(rU) solution, a third of a poly(rU) strand was added, maintaining the ratio 1:1 in the same buffer. The triple helix was left to rest all night in the dark at room temperature; in order to allow formation of poly(rU)*poly(rA)·poly(rU), the exact final concentration (approximately 3.6 × 10^–4^ M in base triplets) was spectrophotometrically obtained using ε(260 nm) = 14 900 cm^–1^ M^–1^. BSA (bovine serum albumin) was supplied by Merck in lyophilized form. The stock solutions were prepared by dissolving known quantities of solid in the 2.5 mM NaCac, pH 7.0 buffer. The concentrations were checked spectrophotometrically (λ = 278 nm, ε = 44 000 M^–1^ cm^–1^.^90^ Ethidium bromide solid (EB, purity > 99%) was obtained from Merck, and the stock solutions were prepared by dissolving known amounts of solid in the 2.5 mM NaCac, pH 7.0 buffer. The concentrations were checked spectrophotometrically (λ = 480 nm, ε = 5600 M^–1^ cm^–1^).^91^ The aqueous solutions were prepared using ultrapure grade water with an AriumPro system (Sartorius).

#### Methods

A Shimazdu 2450 dual beam UV–vis spectrophotometer was used to record the absorption spectra and follow the spectrophotometric titrations. The temperature was kept constant through a Peltier thermostat (±0.1 °C). This spectrophotometer was also used in melting experiments, by heating the working solutions from 25 to 90 °C with a scan rate of 5 °C/min, each step of 6.5 min being composed of 4.5 min rest, 1 min of UV-spectrum recording, and a 1 min temperature increase. A PerkinElmer LS55 spectrofluorometer was used to follow the spectrofluorimetric microtitrations. The temperature was kept constant through connection with a water thermostat (±0.1 °C). This was used to perform metal complex/DNA (+EB) and metal complex/RNA (+EB) exchange titrations as well as metal complex/BSA titrations. Inner-filter effects were verified under the experimental conditions chosen for metal complex/BSA titrations and found negligible, in particular in the case of **5** and **7**; for **6**, only points with absorbance below a certain threshold were used for the numerical evaluation of BSA binding constants (*A* < 0.05).^76^ In both absorbance and fluorescence titrations, the addition of the titrant was done directly in the cell by using a microsyringe connected to a Mitutoyo micrometric screw: this system was calibrated by weight and found to add 8.2 μL for each turn of the screw (1/50 of a turn being the minimum addition possible). A semimicro Cannon-Ubbelohde capillary viscometer was used for viscosity measurements (2.0 mL of solution needed). The apparatus was placed in a thermostatic system that allowed the measurements to be performed at a constant temperature of 25.0 °C (±0.1 °C). We measured (at least five repetitions each, errors as ±SD) the flow times of the buffer (*t*_buffer_), of DNA alone (*t*_DNA_), and of metal complex/DNA mixtures in different concentration ratios (*t*_mixture_). The relative elongation is calculated from the relative viscosity (η/η°) as (η/η°)^1/3^ = (*t*_mixture_ – *t*_buffer_)/(*t*_DNA_ – *t*_buffer_). Windows-Excel and Microcal-Origin 8.0 programs were used for most of the mathematical calculations and graphical representations. The program Hypspec2014 (http://www.hyperquad.co.uk/) was used to calculate the constants for the formation of the metal complex/biosubstrate adducts. This program allows simultaneous interpolation of all the spectra acquired experimentally, provided that the optical signal is proportional to the species concentrations. The algorithm, once the initial estimate of the unknowns (one or more binding constants) has been provided, interpolates the entire data set and returns the values of the unknowns to convergence. The robustness of the results was verified by inserting different initial estimates. In the case of the DNA/RNA spectrophotometric titrations, the range of wavelengths used to fit the data started at 310 nm to minimize any possible influence of nucleic acid excess on the signal of the (free to bound) metal complex. The binding constant values in the tables refer to mean values over repeated experiments.

## Supporting Information Available

NMR spectra of products, behavior in solution, UV–vis data, spectroscopic titrations for CT-DNA, RNA, and BSA binding (PDF)

## References

[ref1] Zeng L.; Gupta P.; Chen Y.; Wang E.; Ji L.; Chao H.; Chen Z.-S. The Development of Anticancer Ruthenium(II) Complexes: From Single Molecule Compounds to Nanomaterials. Chem. Soc. Rev. 2017, 46 (19), 5771–5804. 10.1039/C7CS00195A. 28654103 PMC5624840

[ref2] Anthony E. J.; Bolitho E. M.; Bridgewater H. E.; Carter O. W. L.; Donnelly J. M.; Imberti C.; Lant E. C.; Lermyte F.; Needham R. J.; Palau M.; Sadler P. J.; Shi H.; Wang F.-X.; Zhang W.-Y.; Zhang Z. Metallodrugs Are Unique: Opportunities and Challenges of Discovery and Development. Chem. Sci. 2020, 11 (48), 12888–12917. 10.1039/D0SC04082G. 34123239 PMC8163330

[ref3] Alessio E.; Messori L. NAMI-A and KP1019/1339, Two Iconic Ruthenium Anticancer Drug Candidates Face-to-Face: A Case Story in Medicinal Inorganic Chemistry. Molecules 2019, 24 (10), 1995 10.3390/molecules24101995. 31137659 PMC6571951

[ref4] Thota S.; Rodrigues D. A.; Crans D. C.; Barreiro E. J. Ru(II) Compounds: Next-Generation Anticancer Metallotherapeutics?. J. Med. Chem. 2018, 61 (14), 5805–5821. 10.1021/acs.jmedchem.7b01689. 29446940

[ref5] Steel T. R.; Walsh F.; Wieczorek-Błauż A.; Hanif M.; Hartinger C. G. Monodentately-Coordinated Bioactive Moieties in Multimodal Half-Sandwich Organoruthenium Anticancer Agents. Coord. Chem. Rev. 2021, 439, 213890 10.1016/j.ccr.2021.213890.

[ref6] Murray B. S.; Dyson P. J. Recent Progress in the Development of Organometallics for the Treatment of Cancer. Curr. Opin. Chem. Biol. 2020, 56, 28–34. 10.1016/j.cbpa.2019.11.001. 31812831

[ref7] Murray B. S.; Babak M. V.; Hartinger C. G.; Dyson P. J. The Development of RAPTA Compounds for the Treatment of Tumors. Coord. Chem. Rev. 2016, 306, 86–114. 10.1016/j.ccr.2015.06.014.

[ref8] Rausch M.; Dyson P. J.; Nowak-Sliwinska P. Recent Considerations in the Application of RAPTA-C for Cancer Treatment and Perspectives for Its Combination with Immunotherapies. Adv. Ther. 2019, 2 (9), 1900042 10.1002/adtp.201900042.

[ref9] Weiss A.; Berndsen R. H.; Dubois M.; Müller C.; Schibli R.; Griffioen A. W.; Dyson P. J.; Nowak-Sliwinska P. *In Vivo* Anti-Tumor Activity of the Organometallic Ruthenium(II)-Arene Complex [Ru(η ^6^ - *p* -Cymene)Cl _2_ (Pta)] (RAPTA-C) in Human Ovarian and Colorectal Carcinomas. Chem. Sci. 2014, 5 (12), 4742–4748. 10.1039/C4SC01255K.

[ref10] Rahman F.-U.; Bhatti M. Z.; Ali A.; Duong H.-Q.; Zhang Y.; Ji X.; Lin Y.; Wang H.; Li Z.-T.; Zhang D.-W. Dimetallic Ru(II) Arene Complexes Appended on Bis-Salicylaldimine Induce Cancer Cell Death and Suppress Invasion via P53-Dependent Signaling. Eur. J. Med. Chem. 2018, 157, 1480–1490. 10.1016/j.ejmech.2018.08.054. 30282320

[ref11] Giannini F.; Geiser L.; Paul L. E. H.; Roder T.; Therrien B.; Süss-Fink G.; Furrer J. Tuning the in Vitro Cell Cytotoxicity of Dinuclear Arene Ruthenium Trithiolato Complexes: Influence of the Arene Ligand. J. Organomet. Chem. 2015, 783, 40–45. 10.1016/j.jorganchem.2015.02.010.

[ref12] Zhao J.; Li S.; Wang X.; Xu G.; Gou S. Dinuclear Organoruthenium Complexes Exhibiting Antiproliferative Activity through DNA Damage and a Reactive-Oxygen-Species-Mediated Endoplasmic Reticulum Stress Pathway. Inorg. Chem. 2019, 58 (3), 2208–2217. 10.1021/acs.inorgchem.8b03447. 30675781

[ref13] Nazarov A. A.; Mendoza-Ferri M.-G.; Hanif M.; Keppler B. K.; Dyson P. J.; Hartinger C. G. Understanding the Interactions of Diruthenium Anticancer Agents with Amino Acids. JBIC J. Biol. Inorg. Chem. 2018, 23 (7), 1159–1164. 10.1007/s00775-018-1597-x. 30046902

[ref14] Stíbal D.; Therrien B.; Süss-Fink G.; Nowak-Sliwinska P.; Dyson P. J.; Čermáková E.; Řezáčová M.; Tomšík P. Chlorambucil Conjugates of Dinuclear P-Cymene Ruthenium Trithiolato Complexes: Synthesis, Characterization and Cytotoxicity Study in Vitro and in Vivo. JBIC J. Biol. Inorg. Chem. 2016, 21 (4), 443–452. 10.1007/s00775-016-1353-z. 27040952

[ref15] Studer V.; Anghel N.; Desiatkina O.; Felder T.; Boubaker G.; Amdouni Y.; Ramseier J.; Hungerbühler M.; Kempf C.; Heverhagen J. T.; Hemphill A.; Ruprecht N.; Furrer J.; Păunescu E. Conjugates Containing Two and Three Trithiolato-Bridged Dinuclear Ruthenium(II)-Arene Units as In Vitro Antiparasitic and Anticancer Agents. Pharmaceuticals 2020, 13 (12), 471 10.3390/ph13120471. 33339451 PMC7767221

[ref16] Orts-Arroyo M.; Gutiérrez F.; Gil-Tebar A.; Ibarrola-Villava M.; Jiménez-Martí E.; Silvestre-Llora A.; Castro I.; Ribas G.; Martínez-Lillo J. A Novel Adenine-Based Diruthenium(III) Complex: Synthesis, Crystal Structure, Electrochemical Properties and Evaluation of the Anticancer Activity. J. Inorg. Biochem. 2022, 232, 111812 10.1016/j.jinorgbio.2022.111812. 35421769

[ref17] Alves S. R.; Santos R. L. S. R.; Fornaciari B.; Colquhoun A.; de Oliveira Silva D. A Novel μ-Oxo-Diruthenium(III,III)-Ibuprofen-(4-Aminopyridine) Chloride Derived from the Diruthenium(II,III)-Ibuprofen Paddlewheel Metallodrug Shows Anticancer Properties. J. Inorg. Biochem. 2021, 225, 111596 10.1016/j.jinorgbio.2021.111596. 34601164

[ref18] Wang J.; Zhang Y.; Li Y.; Li E.; Ye W.; Pan J. Dinuclear Organoruthenium Complex for Mitochondria-Targeted Near-Infrared Imaging and Anticancer Therapy to Overcome Platinum Resistance. Inorg. Chem. 2022, 61 (21), 8267–8282. 10.1021/acs.inorgchem.2c00714. 35584546

[ref19] Nyawade E. A.; Friedrich H. B.; Omondi B.; Chenia H. Y.; Singh M.; Gorle S. Synthesis and Characterization of New α,Α′-Diaminoalkane-Bridged Dicarbonyl(η 5 -Cyclopentadienyl)Ruthenium(II) Complex Salts: Antibacterial Activity Tests of η 5 -Cyclopentadienyl Dicarbonyl Ruthenium(II) Amine Complexes. J. Organomet. Chem. 2015, 799–800, 138–146. 10.1016/j.jorganchem.2015.09.007.

[ref20] Johnpeter J. P.; Plasseraud L.; Schmitt F.; Juillerat-Jeanneret L.; Therrien B. Catalytic and Anticancer Activities of Sawhorse-Type Diruthenium Tetracarbonyl Complexes Derived from Fluorinated Fatty Acids. J. Coord. Chem. 2013, 66 (10), 1753–1762. 10.1080/00958972.2013.790020.

[ref21] Nazarov A. A.; Baquié M.; Nowak-Sliwinska P.; Zava O.; van Beijnum J. R.; Groessl M.; Chisholm D. M.; Ahmadi Z.; McIndoe J. S.; Griffioen A. W.; van den Bergh H.; Dyson P. J. Synthesis and Characterization of a New Class of Anti-Angiogenic Agents Based on Ruthenium Clusters. Sci. Rep. 2013, 3 (1), 1485 10.1038/srep01485. 23508096 PMC6504821

[ref22] King P. J.; Knox S. A. R.; McCormick G. J.; Orpen A. G. Synthesis and Reactivity of Dimetallacyclopentenone Complexes [Ru_2_(CO)(μ-CO){μ-C(O)CR1CR2}(η-C_5_H_5_)_2_] (R1 = Me or Ph; R2 = CO_2_Me). J. Chem. Soc., Dalton Trans. 2000, 17, 2975–2982. 10.1039/b003156i.

[ref23] Dyke A. F.; Knox S. A. R.; Naish P. J.; Taylor G. E. Organic Chemistry of Dinuclear Metal Centres. Part 1. Combination of Alkynes with Carbon Monoxide at Di-Iron and Diruthenium Centres: Crystal Structure of [Ru_2_ (CO)(μ-CO){μ-σ:η^3^-C(O)C_2_Ph_2_}(η-C _5_ H _5_)_2_]. J. Chem. Soc., Dalton Trans. 1982, 7, 1297–1307. 10.1039/DT9820001297.

[ref24] Bresciani G.; Zacchini S.; Pampaloni G.; Bortoluzzi M.; Marchetti F. η^6^-Coordinated Ruthenabenzenes from Three-Component Assembly on a Diruthenium μ-Allenyl Scaffold. Dalton Trans. 2022, 51 (21), 8390–8400. 10.1039/D2DT01071B. 35587270

[ref25] Kawahara B.; Gao L.; Cohn W.; Whitelegge J. P.; Sen S.; Janzen C.; Mascharak P. K. Diminished Viability of Human Ovarian Cancer Cells by Antigen-Specific Delivery of Carbon Monoxide with a Family of Photoactivatable Antibody-PhotoCORM Conjugates. Chem. Sci. 2020, 11 (2), 467–473. 10.1039/C9SC03166A. 32190266 PMC7067254

[ref26] Kawahara B.; Moller T.; Hu-Moore K.; Carrington S.; Faull K. F.; Sen S.; Mascharak P. K. Attenuation of Antioxidant Capacity in Human Breast Cancer Cells by Carbon Monoxide through Inhibition of Cystathionine β-Synthase Activity: Implications in Chemotherapeutic Drug Sensitivity. J. Med. Chem. 2017, 60 (19), 8000–8010. 10.1021/acs.jmedchem.7b00476. 28876927

[ref27] Ling K.; Men F.; Wang W.-C.; Zhou Y.-Q.; Zhang H.-W.; Ye D.-W. Carbon Monoxide and Its Controlled Release: Therapeutic Application, Detection, and Development of Carbon Monoxide Releasing Molecules (CORMs): Miniperspective. J. Med. Chem. 2018, 61 (7), 2611–2635. 10.1021/acs.jmedchem.6b01153. 28876065

[ref28] Biancalana L.; Marchetti F. Aminocarbyne Ligands in Organometallic Chemistry. Coord. Chem. Rev. 2021, 449, 214203 10.1016/j.ccr.2021.214203.

[ref29] Singh A.; Lumb I.; Mehra V.; Kumar V. Ferrocene-Appended Pharmacophores: An Exciting Approach for Modulating the Biological Potential of Organic Scaffolds. Dalton Trans. 2019, 48 (9), 2840–2860. 10.1039/C8DT03440K. 30663743

[ref30] Sansook S.; Hassell-Hart S.; Ocasio C.; Spencer J. Ferrocenes in Medicinal Chemistry; a Personal Perspective. J. Organomet. Chem. 2020, 905, 121017 10.1016/j.jorganchem.2019.121017.

[ref31] Patra M.; Gasser G. The Medicinal Chemistry of Ferrocene and Its Derivatives. Nat. Rev. Chem. 2017, 1 (9), 0066 10.1038/s41570-017-0066.

[ref32] Fiala C.; Pasic M. D. Aspirin: Bitter Pill or Miracle Drug?. Clin. Biochem. 2020, 85, 1–4. 10.1016/j.clinbiochem.2020.07.003. 32721423

[ref33] Cheng Q.; Shi H.; Wang H.; Min Y.; Wang J.; Liu Y. The Ligation of Aspirin to Cisplatin Demonstrates Significant Synergistic Effects on Tumor Cells. Chem. Commun. 2014, 50 (56), 7427–7430. 10.1039/C4CC00419A. 24777030

[ref34] Schoch S.; Hadiji M.; Pereira S. A. P.; Saraiva M. L. M. F. S.; Braccini S.; Chiellini F.; Biver T.; Zacchini S.; Pampaloni G.; Dyson P. J.; Marchetti F. A Strategy to Conjugate Bioactive Fragments to Cytotoxic Diiron Bis(Cyclopentadienyl) Complexes. Organometallics 2021, 40 (15), 2516–2528. 10.1021/acs.organomet.1c00270. 34475610 PMC8397425

[ref35] Kowalski K. Insight into the Biological Activity of Organometallic Acetylsalicylic Acid (Aspirin) Derivatives. ChemPlusChem. 2019, 84 (4), 403–415. 10.1002/cplu.201900086. 31939218

[ref36] Bresciani G.; Zacchini S.; Pampaloni G.; Marchetti F. Carbon–Carbon Bond Coupling of Vinyl Molecules with an Allenyl Ligand at a Diruthenium Complex. Organometallics 2022, 41 (8), 1006–1014. 10.1021/acs.organomet.2c00060.

[ref37] Rubner G.; Bensdorf K.; Wellner A.; Kircher B.; Bergemann S.; Ott I.; Gust R. Synthesis and Biological Activities of Transition Metal Complexes Based on Acetylsalicylic Acid as Neo-Anticancer Agents. J. Med. Chem. 2010, 53 (19), 6889–6898. 10.1021/jm101019j. 20857911

[ref38] Schmidt K.; Jung M.; Keilitz R.; Schnurr B.; Gust R. Acetylenehexacarbonyldicobalt Complexes, a Novel Class of Antitumor Drugs. Inorg. Chim. Acta 2000, 306 (1), 6–16. 10.1016/S0020-1693(00)00139-0.

[ref39] Mohamed A. S.; Jourdain I.; Knorr M.; Elmi A.; Chtita S.; Scheel R.; Strohmann C.; Hussien M. A. Design of Hydroxyl- and Thioether-Functionalized Iron-Platinum Dimetallacyclopentenone Complexes. Crystal and Electronic Structures, Hirshfeld and Docking Analyses and Anticancer Activity Evaluated by in Silico Simulation. J. Mol. Struct. 2022, 1251, 131979 10.1016/j.molstruc.2021.131979.

[ref40] Marchetti F. Constructing Organometallic Architectures from Aminoalkylidyne Diiron Complexes. Eur. J. Inorg. Chem. 2018, 2018 (36), 3987–4003. 10.1002/ejic.201800659.

[ref41] Casey C. P.; Ha Y.; Powell D. R. Synthesis and Reactions of Dirhenium Alkenylidene and Alkylidyne Complexes. J. Am. Chem. Soc. 1994, 116 (8), 3424–3428. 10.1021/ja00087a029.

[ref42] Dennett J. N. L.; Knox S. A. R.; Charmant J. P. H.; Gillon A. L.; Orpen A. G. Synthesis and Reactivity of μ-Butadienyl Diruthenium Cations. Inorg. Chim. Acta 2003, 354, 29–40. 10.1016/S0020-1693(03)00391-8.

[ref43] Busetto L.; Maitlis P. M.; Zanotti V. Bridging Vinylalkylidene Transition Metal Complexes. Coord. Chem. Rev. 2010, 254 (5–6), 470–486. 10.1016/j.ccr.2009.07.022.

[ref44] Marchetti F. Alkylidyne and Alkylidene Complexes of Iron. Comprehensive Organometallic Chemistry IV 2022, 210–257. 10.1016/B978-0-12-820206-7.00105-0.

[ref45] Bresciani G.; Boni S.; Zacchini S.; Pampaloni G.; Bortoluzzi M.; Marchetti F. Alkyne–Alkenyl Coupling at a Diruthenium Complex. Dalton Trans. 2022, 51 (41), 15703–15715. 10.1039/D2DT02866B. 36177843

[ref46] Dyke A. F.; Knox S. A. R.; Morris M. J.; Naish P. J. Organic Chemistry of Dinuclear Metal Centres. Part 3. μ-Carbene Complexes of Iron and Ruthenium from Alkynes Viaμ-Vinyl Cations. J. Chem. Soc., Dalton Trans. 1983, 7, 1417–1426. 10.1039/DT9830001417.

[ref47] Dennett J. N. L.; Knox S. A. R.; Anderson K. M.; Charmant J. P. H.; Orpen A. G. The Synthesis of [FeRu(CO) _2_ (μ-CO) _2_ (η-C _5_ H _5_)(η-C _5_ Me _5_)] and Convenient Entries to Its Organometallic Chemistry. Dalton Trans 2005, 1, 63–73. 10.1039/B414412K. 15605148

[ref48] Agonigi G.; Biancalana L.; Lupo M. G.; Montopoli M.; Ferri N.; Zacchini S.; Binacchi F.; Biver T.; Campanella B.; Pampaloni G.; Zanotti V.; Marchetti F. Exploring the Anticancer Potential of Diiron Bis-Cyclopentadienyl Complexes with Bridging Hydrocarbyl Ligands: Behavior in Aqueous Media and *In Vitro* Cytotoxicity. Organometallics 2020, 39 (5), 645–657. 10.1021/acs.organomet.9b00681.

[ref49] Haribabu J.; Sabapathi G.; Tamizh M. M.; Balachandran C.; Bhuvanesh N. S. P.; Venuvanalingam P.; Karvembu R. Water-Soluble Mono- and Binuclear Ru(η ^6^ - *p* -Cymene) Complexes Containing Indole Thiosemicarbazones: Synthesis, DFT Modeling, Biomolecular Interactions, and *In Vitro* Anticancer Activity through Apoptosis. Organometallics 2018, 37 (8), 1242–1257. 10.1021/acs.organomet.8b00004.

[ref50] Rilak A.; Bratsos I.; Zangrando E.; Kljun J.; Turel I.; Bugarčić Ž. D.; Alessio E. New Water-Soluble Ruthenium(II) Terpyridine Complexes for Anticancer Activity: Synthesis, Characterization, Activation Kinetics, and Interaction with Guanine Derivatives. Inorg. Chem. 2014, 53 (12), 6113–6126. 10.1021/ic5005215. 24884156

[ref51] Lameijer L. N.; Hopkins S. L.; Brevé T. G.; Askes S. H. C.; Bonnet S. d - Versus l -Glucose Conjugation: Mitochondrial Targeting of a Light-Activated Dual-Mode-of-Action Ruthenium-Based Anticancer Prodrug.. Chem. Eur. J. 2016, 22 (51), 18484–18491. 10.1002/chem.201603066. 27859843 PMC5214309

[ref52] Davies D. L.; Howard J. A. K.; Knox S. A. R.; Marsden K.; Mead K. A.; Morris M. J.; Rendle M. C. Unusual Tetra- and Penta-Ruthenium Complexes from Linking of Ethylidyne and Vinylidene Ligands. J. Organomet. Chem. 1985, 279 (3), c38–c41. 10.1016/0022-328X(85)87050-9.

[ref53] Adams P. Q.; Davies D. L.; Dyke A. F.; Knox S. A. R.; Mead K. A.; Woodward P. Stereochemical Control of Alkyne Oligomerisation at a Diruthenium Centre: X-Ray Structures of [Ru _2_ (CO)(μ-CO)(μ-C _4_ H _4_ CMe _2_)(η-C _5_ H _5_) _2_ ] and [Ru _2_ (μ-CO){μ-C _4_ (CO _2_ Me) _4_ CH _2_ }(η-C _5_ H _5_) _2_ ]. J. Chem. Soc. Chem. Commun. 1983, 5, 222–224. 10.1039/C39830000222.

[ref54] Colborn R. E.; Dyke A. F.; Knox S. A. R.; Mead K. A.; Woodward P. Organic Chemistry of Dinuclear Metal Centres. Part 4. μ-Carbene and μ-Vinyl Complexes of Ruthenium from Allenes. J. Chem. Soc., Dalton Trans. 1983, 9, 2099–2108. 10.1039/DT9830002099.

[ref55] Akita M.; Hua R.; Knox S. A. R.; Moro-oka Y.; Nakanishi S.; Yates M. I. Specific C–C Coupling of the Labile Diruthenium Bridging Methylene Complex, Cp_2_Ru_2_(μ-CH_2_)(CO)_2_(MeCN), with Diazoalkanes (R_2_C = N_2_) Leading to Alkenyl Complexes, Cp_2_Ru_2_(μ-CH=CR_2_)(μ-H)(CO)_2_, and Alkenes, CH_2_=CR_2_. J. Organomet. Chem. 1998, 569 (1–2), 71–83. 10.1016/S0022-328X(98)00775-X.

[ref56] Knox S. A. R.; Marchetti F. Additions and Intramolecular Migrations of Nucleophiles in Cationic Diruthenium μ-Allenyl Complexes. J. Organomet. Chem. 2007, 692 (19), 4119–4128. 10.1016/j.jorganchem.2007.06.029.

[ref57] Adams K. J.; Barker J. J.; Charmant J. P. H.; Ganter C.; Klatt G.; Knox S. A. R.; Orpen A. G.; Ruile S. Tri- and Tetra-Nuclear μ-Alkyne Clusters from [Ru _2_ (μ-CO)(μ-C _2_ R _2_)(η-C _5_ H _5_) _2_ ](R = Ph or CF _3_). J. Chem. Soc., Dalton Trans. 1994, 4, 477–484. 10.1039/DT9940000477.

[ref58] Casey C. P.; Vosejpka P. C.; Crocker M. Reactions of Nucleophiles with Cationic Bridging Alkylidyne Complexes. J. Organomet. Chem. 1990, 394 (1–3), 339–347. 10.1016/0022-328X(90)87243-7.

[ref59] Boni A.; Marchetti F.; Pampaloni G.; Zacchini S. Cationic Diiron and Diruthenium μ-Allenyl Complexes: Synthesis, X-Ray Structures and Cyclization Reactions with Ethyldiazoacetate/Amine Affording Unprecedented Butenolide- and Furaniminium-Substituted Bridging Carbene Ligands. Dalton Trans. 2010, 39 (45), 10866 10.1039/c0dt00697a. 20953481

[ref60] Casey C. P.; Marder S. R.; Adams B. R. Interconversion of.Mu.-Alkylidyne and.Mu.-Alkenyl Diiron Complexes. J. Am. Chem. Soc. 1985, 107 (25), 7700–7705. 10.1021/ja00311a079.

[ref61] Albano V. G.; Busetto L.; Marchetti F.; Monari M.; Zacchini S.; Zanotti V. Hydride Addition at μ-Vinyliminium Ligand Obtained from Disubstituted Alkynes. J. Organomet. Chem. 2005, 690 (4), 837–846. 10.1016/j.jorganchem.2004.10.025.

[ref62] Chen K.-H.; Feng Y. J.; Ma H.-W.; Lin Y.-C.; Liu Y.-H.; Kuo T.-S. Cyclization Accompanied with 1,2-Phenyl Migration in the Protonation of Ruthenium Acetylide Complex Containing an Allenyl Group. Organometallics 2010, 29 (24), 6829–6836. 10.1021/om101017p.

[ref63] Colborn R. E.; Davies D. L.; Dyke A. F.; Endesfelder A.; Knox S. A. R.; Orpen A. G.; Plaas D. Organic Chemistry of Binuclear Metal Centres. Part 6. μ-Vinylidene and μ-Ethylidyne Diruthenium Complexes: Crystal Structures of Cis-[Ru _2_ (CO) _2_ (μ-CO)(μ-CCH _2_)(η-C _5_ H _5_) _2_ ] and Cis-[Ru _2_ (CO) _2_ (μ-CO)(μ-CMe)(η-C _5_ H _5_) _2_ ][BF _4_ ]. J. Chem. Soc., Dalton Trans. 1983, 12, 2661–2668. 10.1039/DT9830002661.

[ref64] Orpen A. G. Structural Chemistry of Binuclear Metal Centres. Crystal and Molecular Structures of the μ-Vinyl and μ-Methylcarbene Complexes [Fe _2_ (CO) _2_ (μ-CO)(μ-CHCH _2_)(η-C _5_ H _5_) _2_ ][BF _4_ ] and [Fe _2_ (CO) _2_ (μ-CO)(μ-CHMe)(η-C _5_ H _5_) _2_ ]. J. Chem. Soc., Dalton Trans. 1983, 7, 1427–1431. 10.1039/DT9830001427.

[ref65] Boni A.; Funaioli T.; Marchetti F.; Pampaloni G.; Pinzino C.; Zacchini S. Reversible Reductive Dimerization of Diiron μ-Vinyl Complex via C–C Coupling: Characterization and Reactivity of the Intermediate Radical Species. Organometallics 2011, 30 (15), 4115–4122. 10.1021/om200421a.

[ref66] Biancalana L.; De Franco M.; Ciancaleoni G.; Zacchini S.; Pampaloni G.; Gandin V.; Marchetti F. Easily Available, Amphiphilic Diiron Cyclopentadienyl Complexes Exhibit in Vitro Anticancer Activity in 2D and 3D Human Cancer Cells through Redox Modulation Triggered by CO Release.. Chem. Eur. J. 2021, 27 (39), 10169–10185. 10.1002/chem.202101048. 34106495 PMC8362065

[ref67] Campanella B.; Braccini S.; Bresciani G.; De Franco M.; Gandin V.; Chiellini F.; Pratesi A.; Pampaloni G.; Biancalana L.; Marchetti F. The Choice of μ-Vinyliminium Ligand Substituents Is Key to Optimize the Antiproliferative Activity of Related Diiron Complexes. Metallomics 2023, 15 (1), mfac096 10.1093/mtomcs/mfac096. 36515681

[ref68] Schoch S.; Braccini S.; Biancalana L.; Pratesi A.; Funaioli T.; Zacchini S.; Pampaloni G.; Chiellini F.; Marchetti F. When Ferrocene and Diiron Organometallics Meet: Triiron Vinyliminium Complexes Exhibit Strong Cytotoxicity and Cancer Cell Selectivity. Inorg. Chem. Front. 2022, 9 (20), 5118–5139. 10.1039/D2QI00534D.

[ref69] Zisowsky J.; Koegel S.; Leyers S.; Devarakonda K.; Kassack M. U.; Osmak M.; Jaehde U. Relevance of Drug Uptake and Efflux for Cisplatin Sensitivity of Tumor Cells. Biochem. Pharmacol. 2007, 73 (2), 298–307. 10.1016/j.bcp.2006.10.003. 17097621

[ref70] Sharma B.; Kumar V. Has Ferrocene Really Delivered Its Role in Accentuating the Bioactivity of Organic Scaffolds?. J. Med. Chem. 2021, 64 (23), 16865–16921. 10.1021/acs.jmedchem.1c00390. 34792350

[ref71] Hwang E.; Jung H. S. Metal–Organic Complex-Based Chemodynamic Therapy Agents for Cancer Therapy. Chem. Commun. 2020, 56 (60), 8332–8341. 10.1039/D0CC03012K. 32515445

[ref72] McGhee J. D.; Von Hippel P. H. Theoretical Aspects of DNA-Protein Interactions: Co-Operative and Non-Co-Operative Binding of Large Ligands to a One-Dimensional Homogeneous Lattice. J. Mol. Biol. 1974, 86 (2), 469–489. 10.1016/0022-2836(74)90031-X. 4416620

[ref73] Chaires J. B. A Thermodynamic Signature for Drug–DNA Binding Mode. Arch. Biochem. Biophys. 2006, 453 (1), 26–31. 10.1016/j.abb.2006.03.027. 16730635

[ref74] Bathaie S. Z.; Nikfarjam L.; Rahmanpour R.; Moosavi-Movahedi A. A. Spectroscopic Studies of the Interaction of Aspirin and Its Important Metabolite, Salicylate Ion, with DNA, A·T and G·C Rich Sequences. Spectrochim. Acta. A. Mol. Biomol. Spectrosc. 2010, 77 (5), 1077–1083. 10.1016/j.saa.2010.08.078. 20869297

[ref75] Pilch D. S.; Kirolos M. A.; Liu X.; Plum G. E.; Breslauer K. J. Berenil [1,3-Bis(4’-Amidinophenyl)Triazene] Binding to DNA Duplexes and to a RNA Duplex: Evidence for Both Intercalative and Minor Groove Binding Properties. Biochemistry 1995, 34 (31), 9962–9976. 10.1021/bi00031a019. 7632695

[ref76] Macii F.; Biver T. Spectrofluorimetric Analysis of the Binding of a Target Molecule to Serum Albumin: Tricky Aspects and Tips. J. Inorg. Biochem. 2021, 216, 111305 10.1016/j.jinorgbio.2020.111305. 33261935

[ref77] Pérez-Arnaiz C.; Leal J.; Busto N.; Carrión M. C.; Rubio A. R.; Ortiz I.; Barone G.; Díaz De Greñu B.; Santolaya J.; Leal J. M.; Vaquero M.; Jalón F. A.; Manzano B. R.; García B. Role of Seroalbumin in the Cytotoxicity of *Cis-* Dichloro Pt(II) Complexes with (N^N)-Donor Ligands Bearing Functionalized Tails. Inorg. Chem. 2018, 57 (10), 6124–6134. 10.1021/acs.inorgchem.8b00713. 29722534

[ref78] Tȏpala T.; Pascual-Álvarez A.; Moldes-Tolosa M. Á.; Bodoki A.; Castiñeiras A.; Torres J.; Del Pozo C.; Borrás J.; Alzuet-Piña G. New Sulfonamide Complexes with Essential Metal Ions [Cu (II), Co (II), Ni (II) and Zn (II)]. Effect of the Geometry and the Metal Ion on DNA Binding and Nuclease Activity. BSA Protein Interaction. J. Inorg. Biochem. 2020, 202, 110823 10.1016/j.jinorgbio.2019.110823. 31706181

[ref79] Berndsen R. H.; Weiss A.; Abdul U. K.; Wong T. J.; Meraldi P.; Griffioen A. W.; Dyson P. J.; Nowak-Sliwinska P. Combination of Ruthenium(II)-Arene Complex [Ru(η^6^-p-Cymene)Cl_2_(Pta)] (RAPTA-C) and the Epidermal Growth Factor Receptor Inhibitor Erlotinib Results in Efficient Angiostatic and Antitumor Activity. Sci. Rep. 2017, 7 (1), 43005 10.1038/srep43005. 28223694 PMC5320450

[ref80] Menges F. Spectragryph - Optical Spectroscopy Software, 2016. http://www.effemm2.de/spectragryph.

[ref81] Fulmer G. R.; Miller A. J. M.; Sherden N. H.; Gottlieb H. E.; Nudelman A.; Stoltz B. M.; Bercaw J. E.; Goldberg K. I. NMR Chemical Shifts of Trace Impurities: Common Laboratory Solvents, Organics, and Gases in Deuterated Solvents Relevant to the Organometallic Chemist. Organometallics 2010, 29 (9), 2176–2179. 10.1021/om100106e.

[ref82] Willker W.; Leibfritz D.; Kerssebaum R.; Bermel W. Gradient Selection in Inverse Heteronuclear Correlation Spectroscopy. Magn. Reson. Chem. 1993, 31 (3), 287–292. 10.1002/mrc.1260310315.

[ref83] Sheldrick G. M. Crystal Structure Refinement with *SHELXL*. Acta Crystallogr. Sect. C Struct. Chem. 2015, 71 (1), 3–8. 10.1107/S2053229614024218. 25567568 PMC4294323

[ref84] Biancalana L.; Batchelor L. K.; Funaioli T.; Zacchini S.; Bortoluzzi M.; Pampaloni G.; Dyson P. J.; Marchetti F. α-Diimines as Versatile, Derivatizable Ligands in Ruthenium(II) *p* -Cymene Anticancer Complexes. Inorg. Chem. 2018, 57 (11), 6669–6685. 10.1021/acs.inorgchem.8b00882. 29790340

[ref85] Zanda E.; Busto N.; Biancalana L.; Zacchini S.; Biver T.; Garcia B.; Marchetti F. Anticancer and Antibacterial Potential of Robust Ruthenium(II) Arene Complexes Regulated by Choice of α-Diimine and Halide Ligands. Chem. Biol. Interact. 2021, 344, 109522 10.1016/j.cbi.2021.109522. 34029541

[ref86] Bresciani G.; Busto N.; Ceccherini V.; Bortoluzzi M.; Pampaloni G.; Garcia B.; Marchetti F. Screening the Biological Properties of Transition Metal Carbamates Reveals Gold(I) and Silver(I) Complexes as Potent Cytotoxic and Antimicrobial Agents. J. Inorg. Biochem. 2022, 227, 111667 10.1016/j.jinorgbio.2021.111667. 34826692

[ref87] Biver T.; Lombardi D.; Secco F.; Rosaria Tiné M.; Venturini M.; Bencini A.; Bianchi A.; Valtancoli B. Kinetic and Equilibrium Studies on the Polyazamacrocycle Neotetren: Metal–Complex Formation and DNA Interaction. Dalton Trans 2006, 12, 1524–1533. 10.1039/B512820J. 16538271

[ref88] Felsenfeld G.; Hirschman S. Z. A Neighbor-Interaction Analysis of the Hypochromism and Spectra of DNA. J. Mol. Biol. 1965, 13 (2), 407–427. 10.1016/S0022-2836(65)80106-1. 5867028

[ref89] Biver T.; Secco F.; Venturini M. Relaxation Kinetics of the Interaction between RNA and Metal-Intercalators: The Poly(A)·Poly(U)/Platinum-Proflavine System. Arch. Biochem. Biophys. 2005, 437 (2), 215–223. 10.1016/j.abb.2005.03.009. 15850561

[ref90] Gill S. C.; Von Hippel P. H. Calculation of Protein Extinction Coefficients from Amino Acid Sequence Data. Anal. Biochem. 1989, 182 (2), 319–326. 10.1016/0003-2697(89)90602-7. 2610349

[ref91] Waring M. J. Complex Formation between Ethidium Bromide and Nucleic Acids. J. Mol. Biol. 1965, 13 (1), 269–282. 10.1016/S0022-2836(65)80096-1. 5859041

