# Supplementary material for: Adding Diversity to a Diruthenium Biscyclopentadienyl Scaffold via Alkyne Incorporation: Synthesis and Biological Studies
Source: Inorg Chem. 2023 Jul 21;62(31):12453–67. doi: 10.1021/acs.inorgchem.3c01644 (PMC10410612; doi:10.1021/acs.inorgchem.3c01644)
Supplement: Supplementary file 1 — ic3c01644_si_001.pdf [file ic3c01644_si_001.pdf]

Supporting Information

Adding Diversity to Diruthenium Bis-Cyclopentadienyl Scaffold via Alkyne Incorporation: Synthesis and Biological Studies

Giulio Bresciani,[a,*] Serena Boni,[a] Tiziana Funaioli,[a] Stefano Zacchini,[b] Guido Pampaloni,[a] Natalia Busto,[c,*] Tarita Biver,[a,*] Fabio Marchetti [a]

[a] University of Pisa, Dipartimento di Chimica e Chimica Industriale, Via G. Moruzzi 13, I-56124 Pisa, Italy.

[b] University of Bologna, Dipartimento di Chimica Industriale "Toso Montanari", Viale del Risorgimento 4, I-40136 Bologna, Italy.

[c] University of Burgos, Departamento de Química, Plaza Misael Bañuelos s/n, 09001 Burgos, Spain.

Corresponding Authors

*E-mail addresses: giulio.bresciani@dcci.unipi.it; nbusto@ubu.es; tarita.biver@unipi.it.

<u>**Table of contents**</u> *Pages*

Figures S1-S11: ^{1}H and ^{13}C NMR spectra S2-S7

Figures S12-S14, **Table S1**: behavior in aqueous solution S8-S9

Figures S15-S20: CT-DNA binding S10-S13

Figures S21-S28, **Table S2**: RNA binding S14-S21

Figures S29-S31, **Table S3**: BSA binding S22-S25

Figure S1. ^{1}H NMR spectrum (401 MHz, $CDCl_3$) of **2a**.

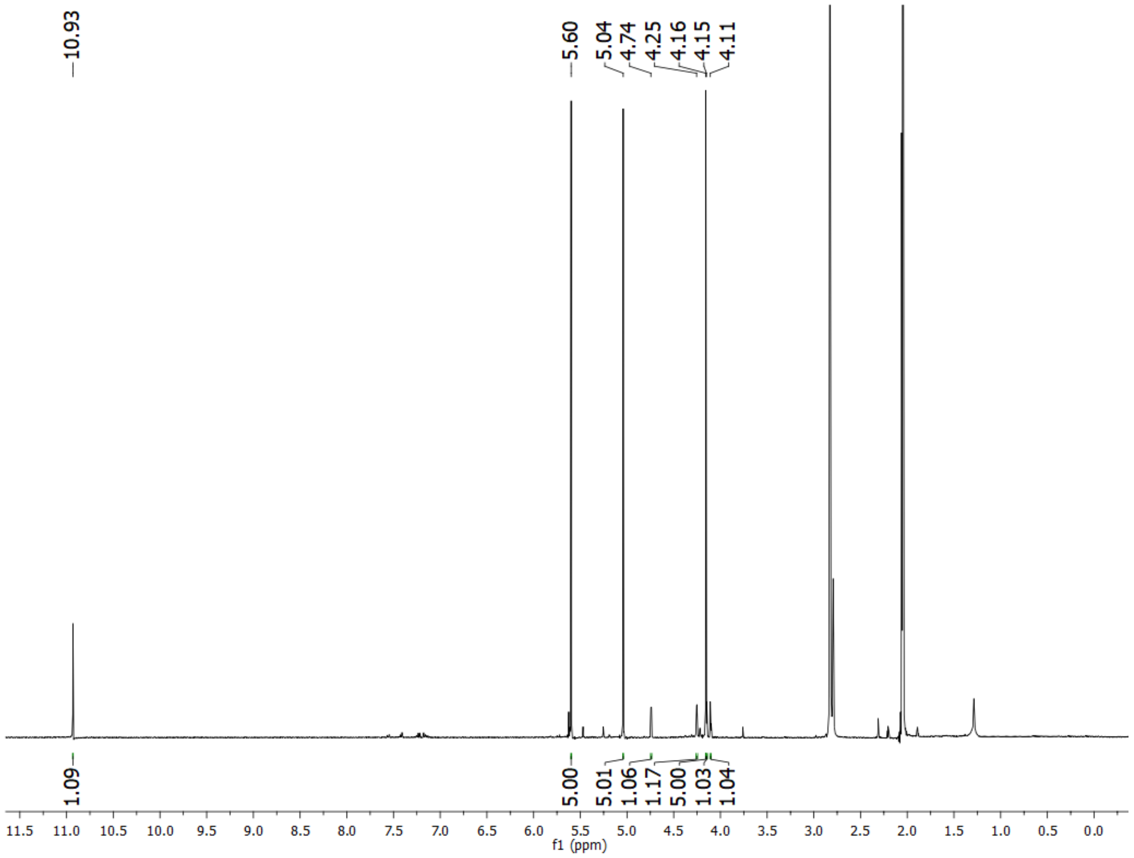

Figure S2. $^{13}C\{^{1}H\}$ NMR spectrum (101 MHz, $CDCl_3$) of **2a**.

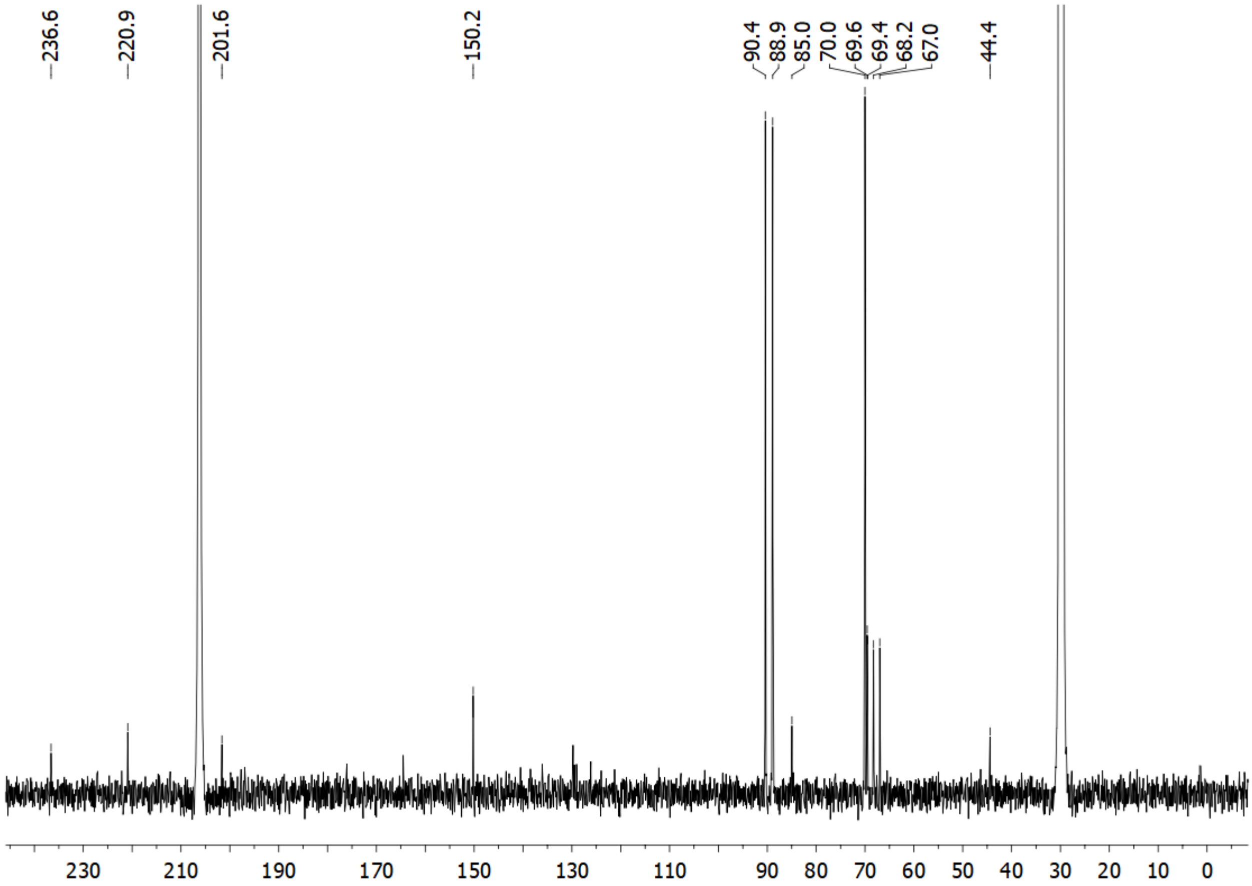

Figure S3. ^{1}H NMR spectrum (401 MHz, $CDCl_3$) of **3a/3b**.

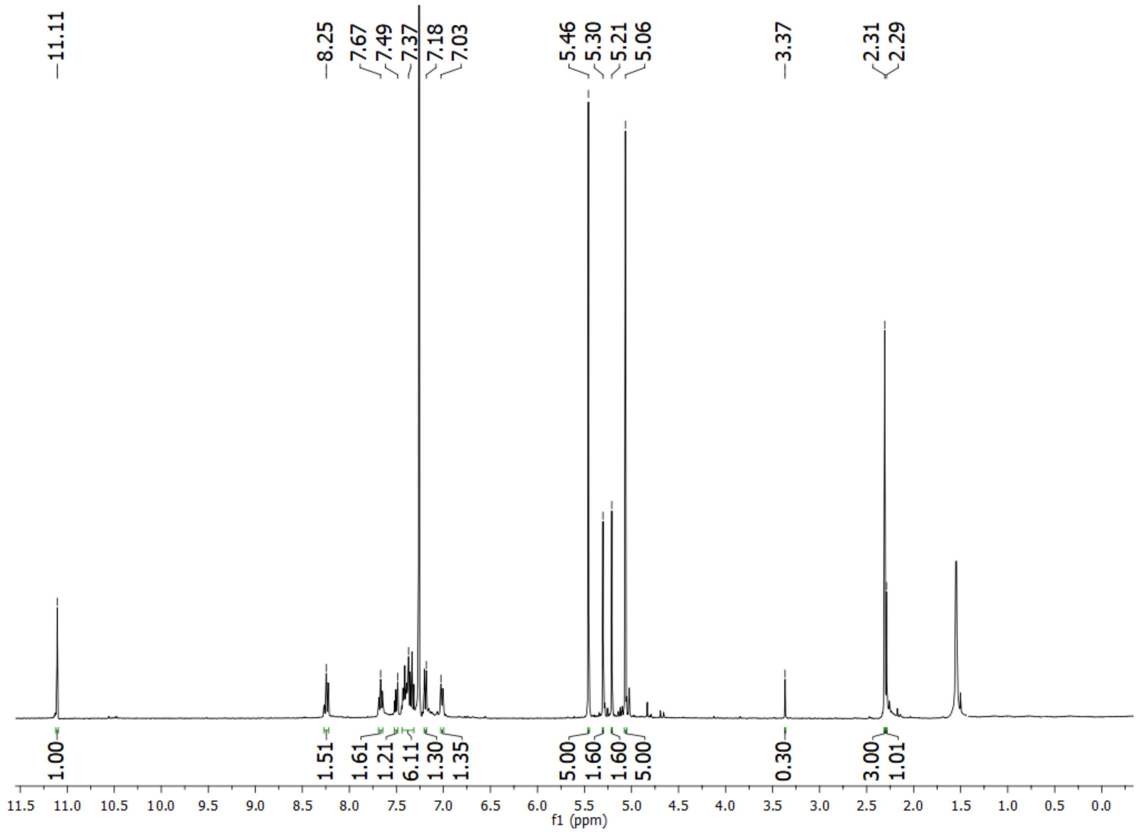

Figure S4. $^{13}C\{^{1}H\}$ NMR spectrum (101 MHz, $CDCl_3$) of **3a/3b.**

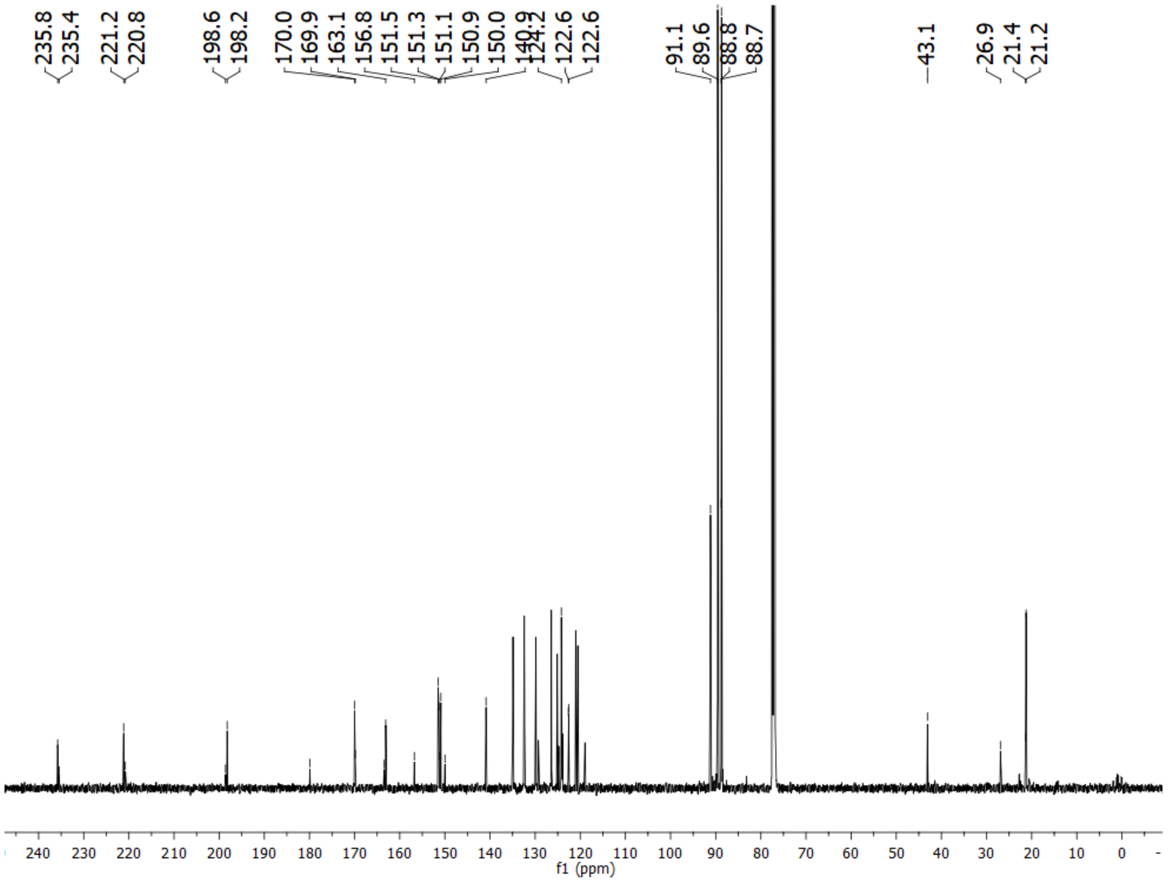

Figure S5. ^{1}H NMR spectrum (401 MHz, $CDCl_3$) of **4a/4b**.

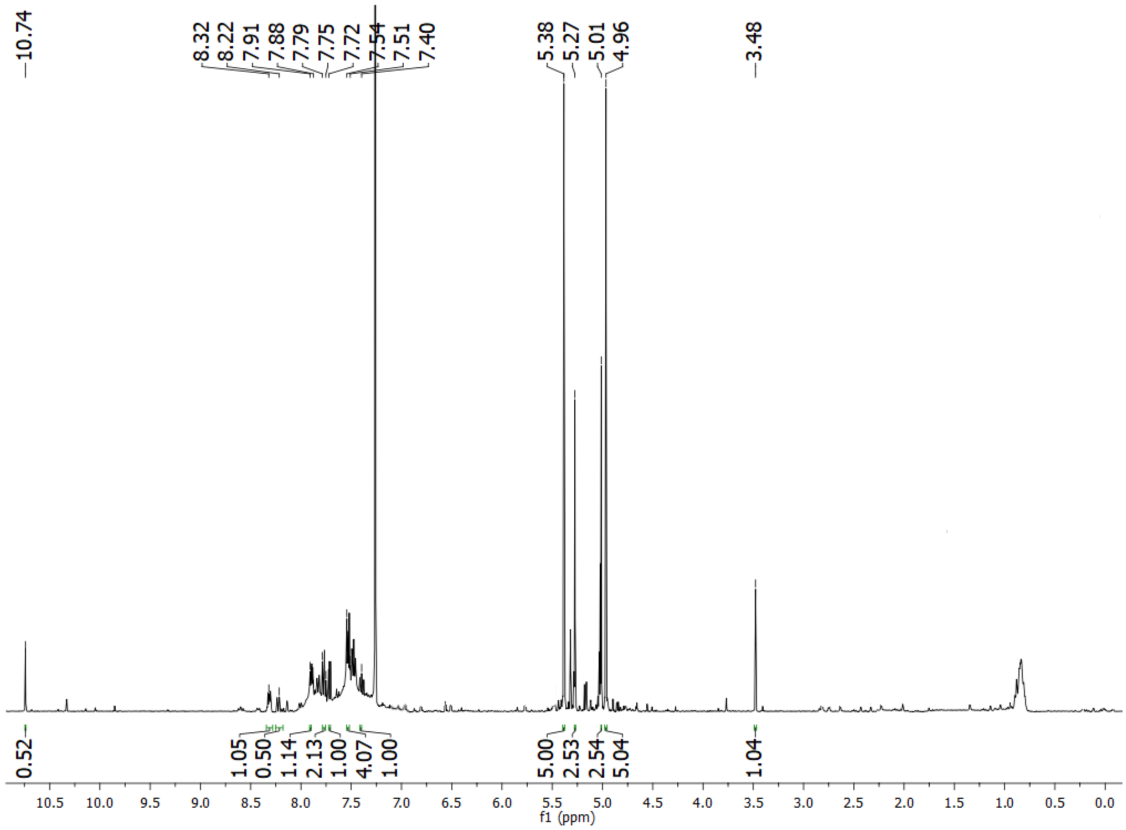

Figure S6. $^{13}C\{^{1}H\}$ NMR spectrum (101 MHz, $CDCl_3$) of **4a/4b**.

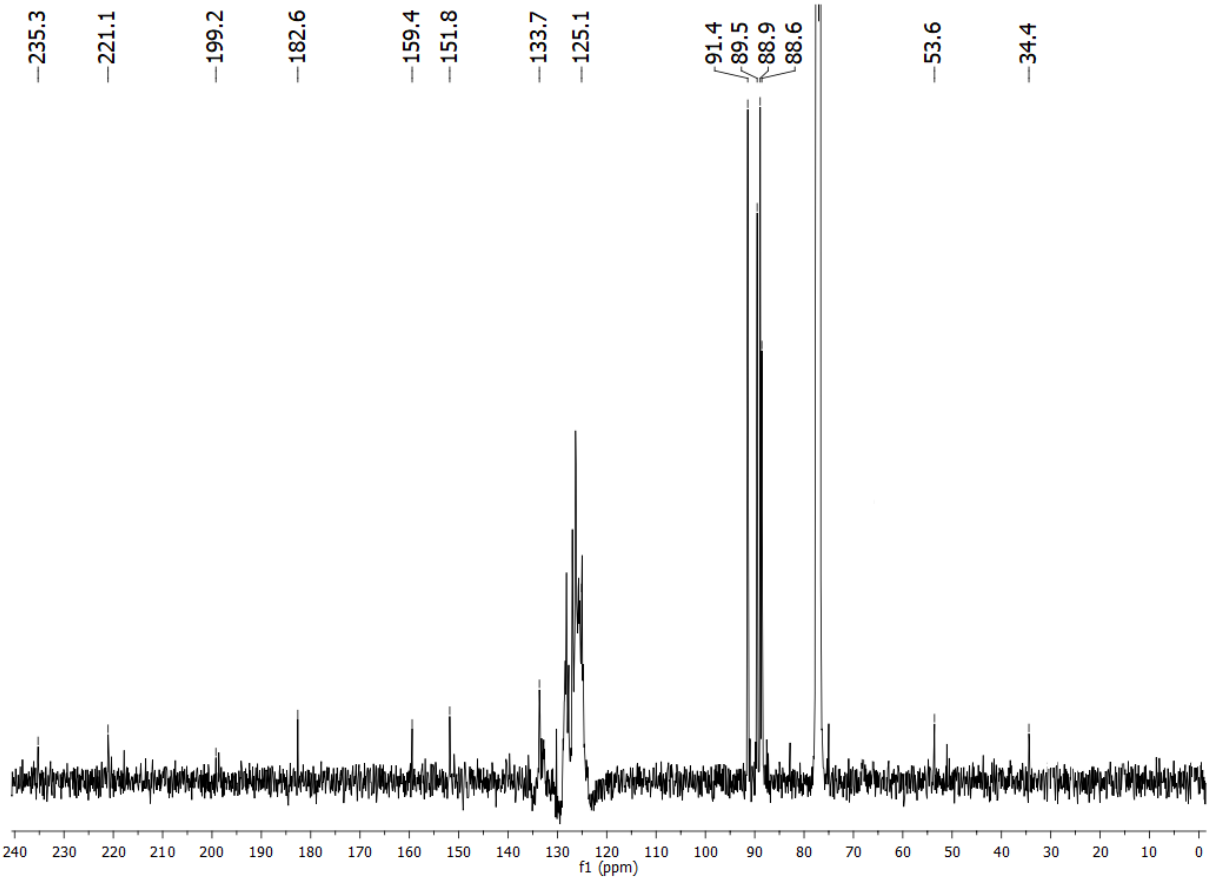

Figure S7. ^{1}H NMR spectrum (401 MHz, 193K, acetone-d_6) of **[5a]BF$_4$ / [5b]BF$_4$**.

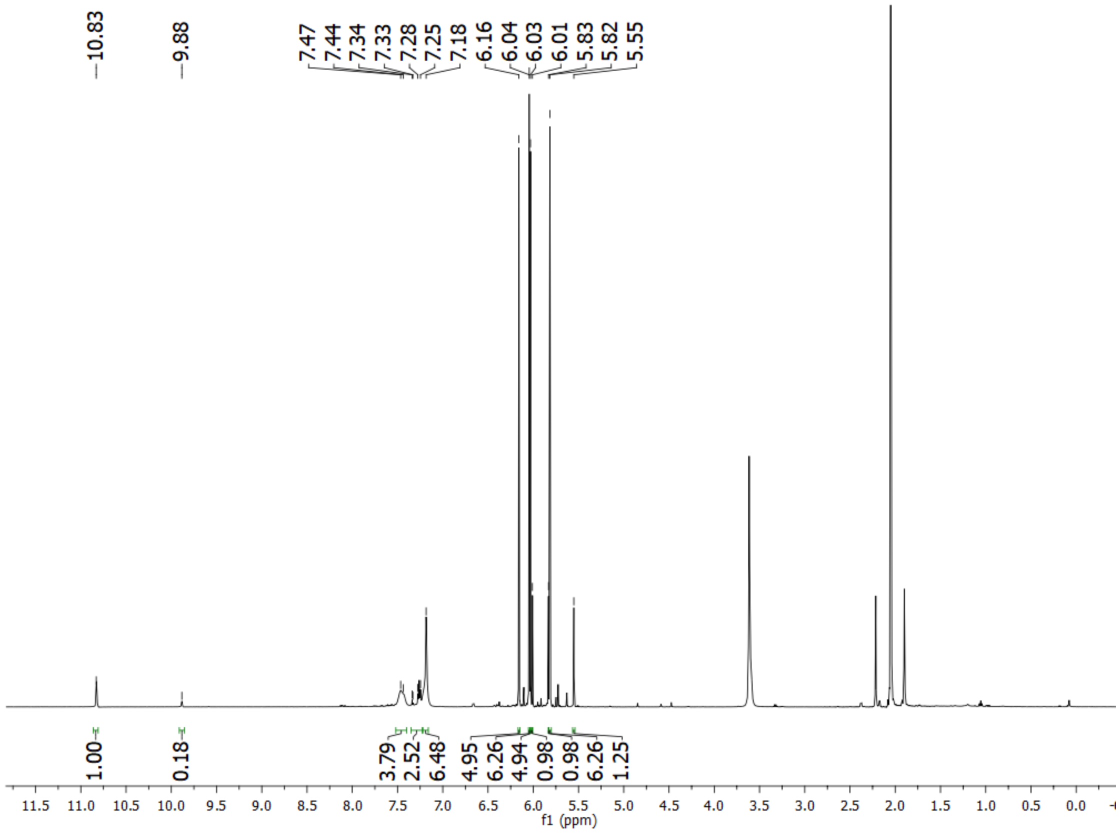

Figure S8. ^{1}H NMR spectrum (401 MHz, 183K, acetone-d_6) of **[6a]BF$_4$ / [6b]BF$_4$**.

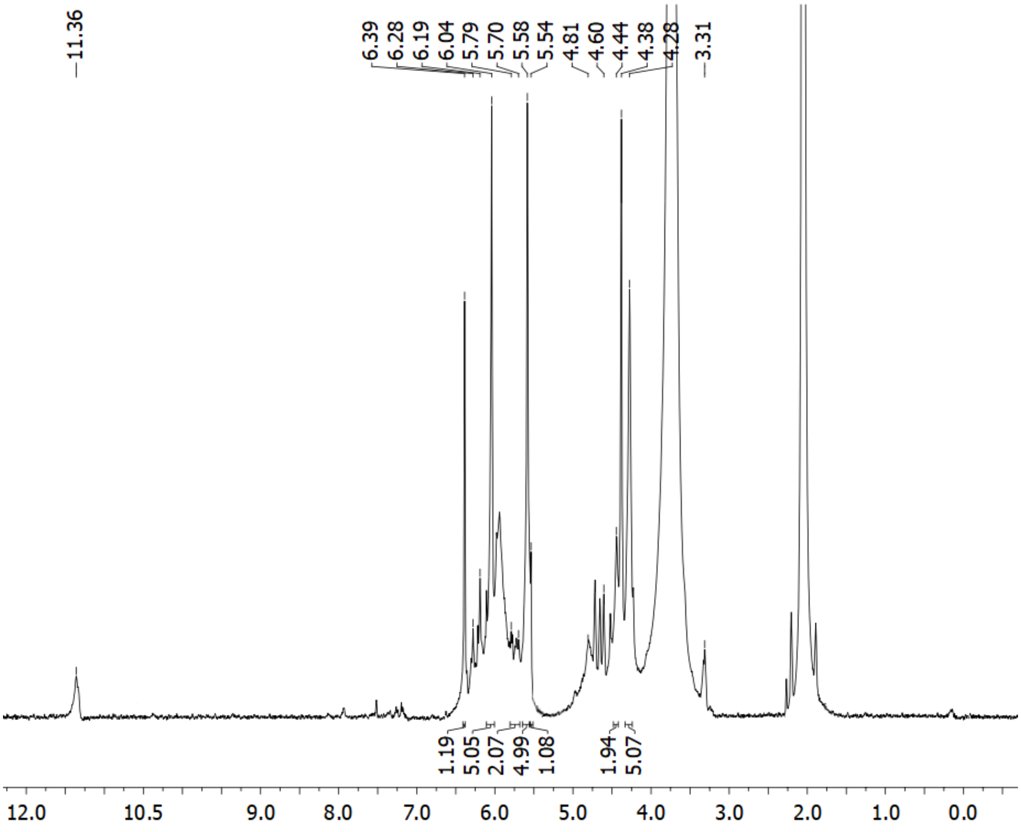

Figure S9. ^{1}H NMR spectrum (401 MHz, 223K, acetone-d_6) of **[7b]BF$_4$**.

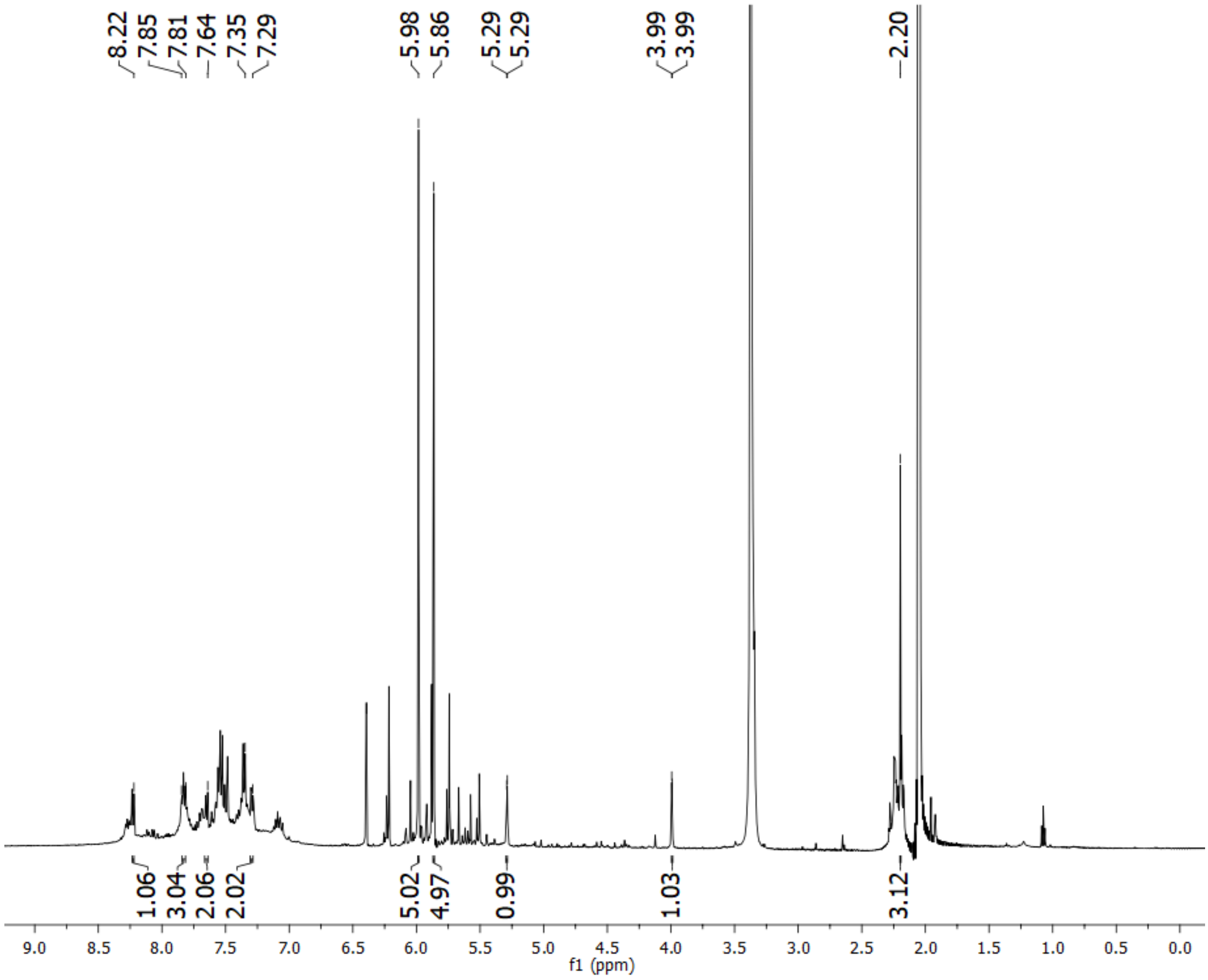

Figure S10. ^{1}H NMR spectrum (401 MHz, toluene-d$_8$) of **2a** (CH and Cp region) at different temperatures.

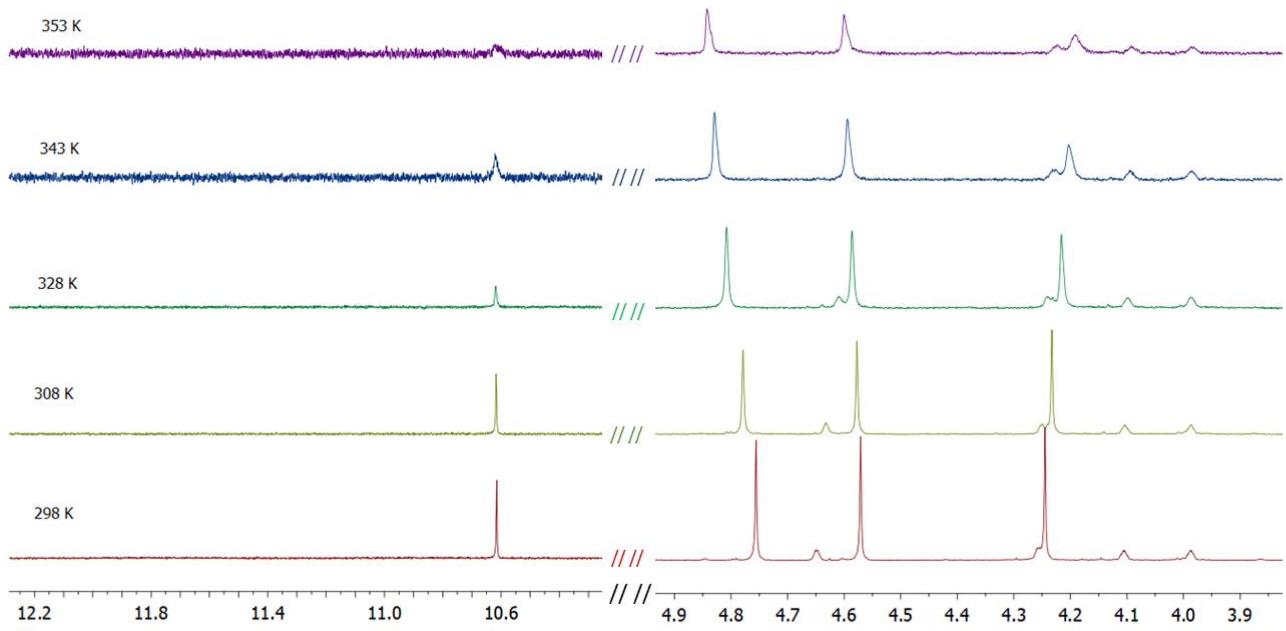

Figure S11. ^{1}H NMR spectrum (401 MHz, toluene-d$_8$) of **3a-b** at different temperatures.

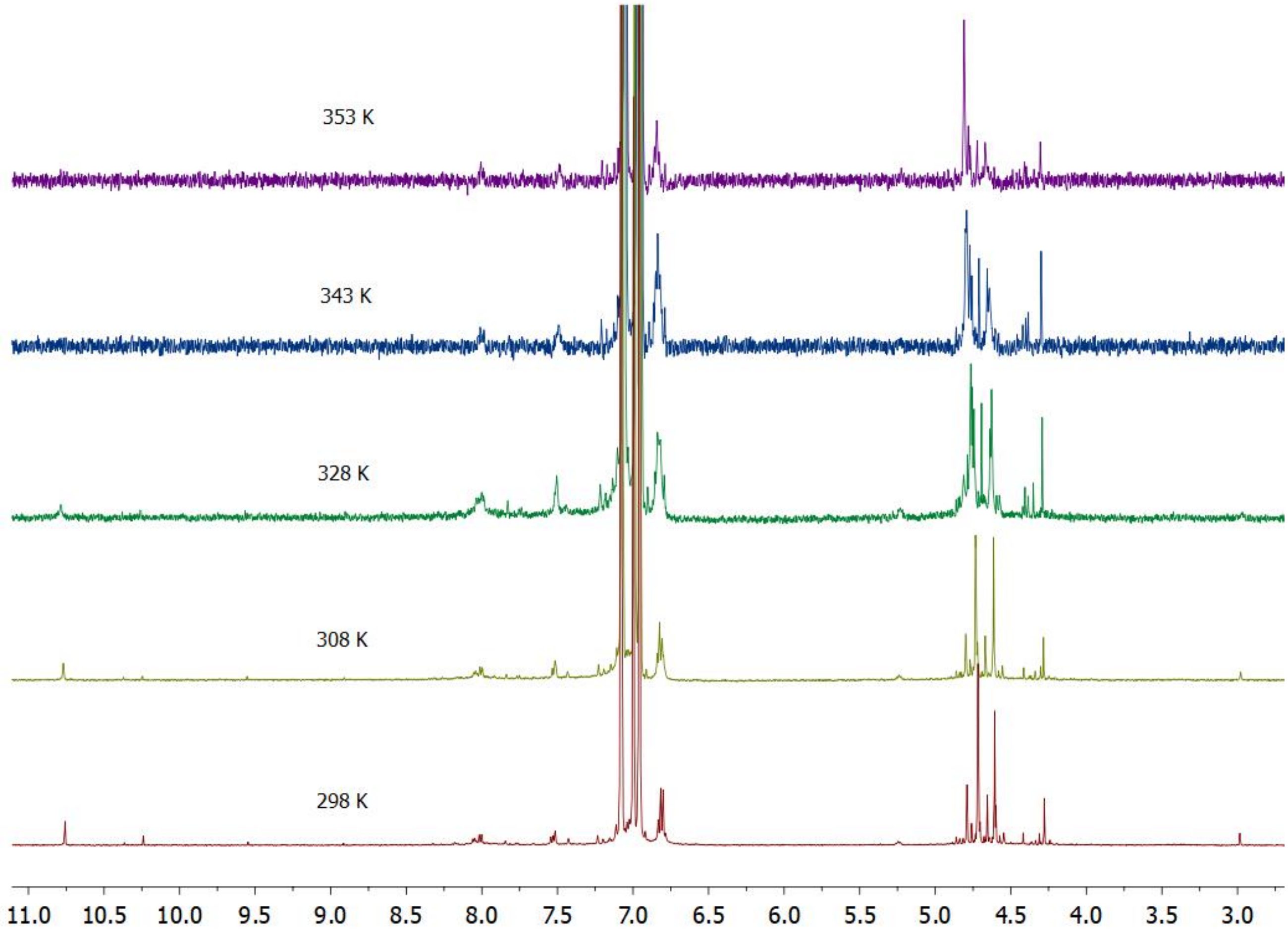

Table S1. Behaviour of diruthenium complexes in aqueous solutions (UV-vis analyses, see Experimental for details). Partition coefficients (Log P_{ow}) at 21±1 °C; relative stability in DMSO-DMEM (ca. 1:4 v/v) solutions after 24 h at 37 °C.

Complex	Log P_{ow}	Residual complex % in DMSO-DMEM
**1**	1.24 ± 0.16	42
**2**	1.37 ± 0.19	65
**5**	0.52 ± 0.06	63
**6**	–0.37 ± 0.04	57
**7**	–0.27 ± 0.03	58

Figure S12. UV-vis absorbance spectra of **5[BF$_4$]** (····), **6[BF$_4$]** (- · - · -), **7[BF$_4$]** (–); NaCac 2.5 mM, pH = 7.0, T = 25.0 °C. Note that blank tests confirmed that none of the compounds is fluorescent.

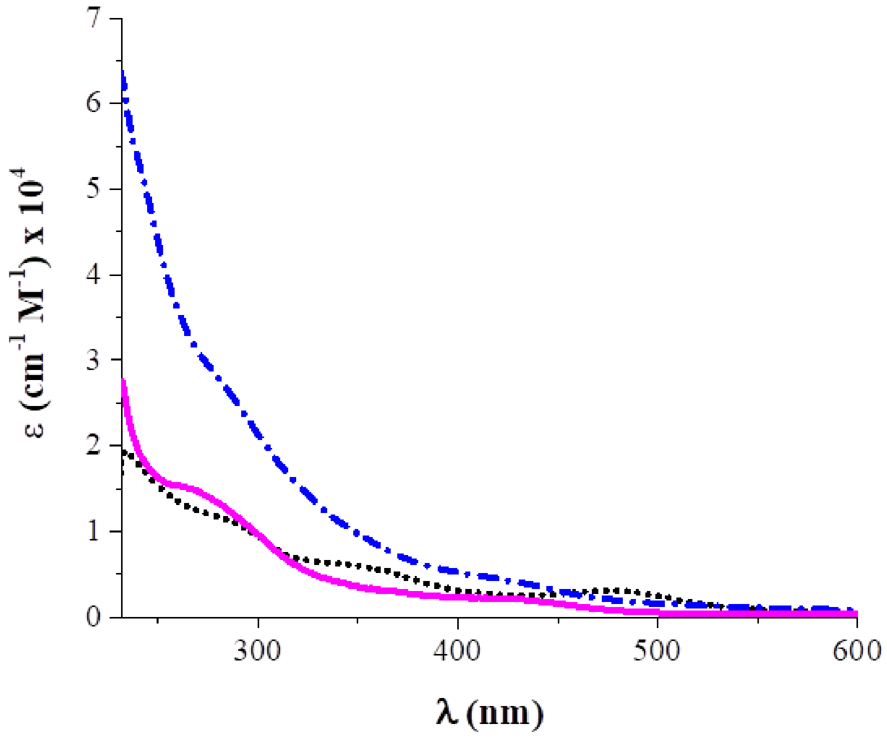

Figure S13. Example of the trends of the spectral profiles of the complexes under study following heating of the solution: (A) spectra of **6** at 25.0 °C (full) and 95.0 °C (dashed); (B) graph of the change in absorbance as a function of temperature (λ = 295 nm) expressed in a similar way to the melting graphs, i.e. as a percentage in absorbance change, A% = (A - A_0) / (A_∞ - A_0); C_6 = 1.39 × 10^{-5} M, NaCac 2.5 mM, pH = 7.0. The UV-vis spectra show only small drifts to a point where the bands tend to zero (A). The inflection point of the sigmoidal plot absorbance vs. temperature (B) yields a breakdown limiting temperature of 66 ± 1 °C. The same occurs for **5** and **7**: the breakdown limiting temperature is 51 ± 1°C for **5** and 73 ± 1 °C for **7**.

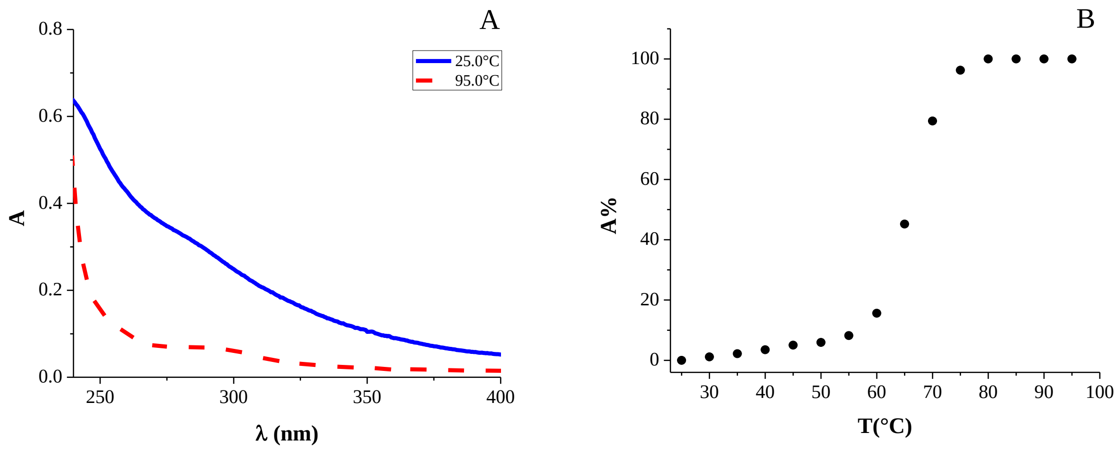

Figure S14. Lambert-Beer plots in NaCac 2.5 mM, pH 7.0 and at 25.0 °C: (A) **5**, λ = 287 nm; (B) **6**, λ = 380 nm; (C) **7**, λ = 290 nm. The linearity of the plots is fully obeyed in the 0 to 10^{-4} M concentration range.

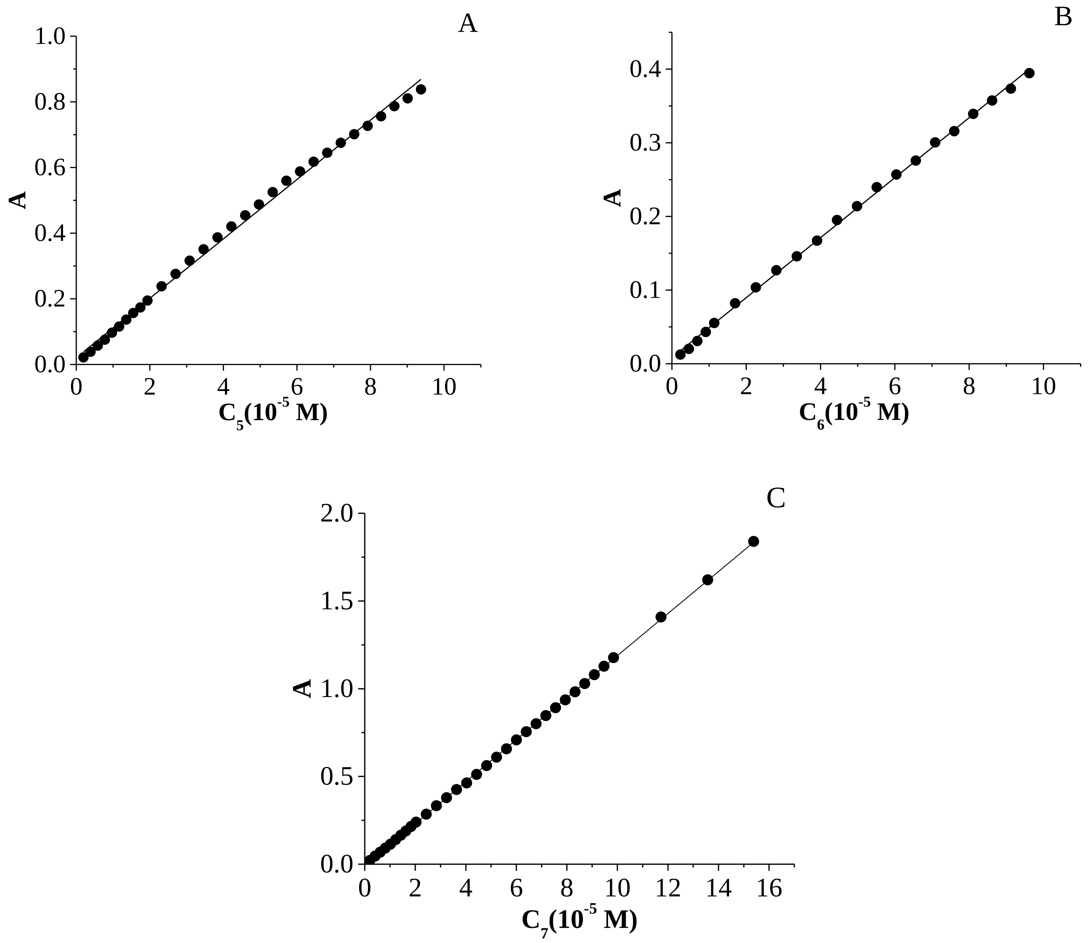

Figure S15. Absorbance spectra (A) and binding isotherm (B) at λ = 320 nm for the **5**/CT-DNA system: C_5 = 3.05×10^{-5} M, C_{DNA} = 0 M (–) to 4.90×10^{-5} M (- -); DMSO 1% v/v, NaCac 2.5 mM, pH = 7.0, T = 25.0°C. (C) HypSpec2014 analysis of the spectrophotometric titration. Left panel: titration curve at 320 nm (open diamond = experimental, cross = calculated) and species distribution (green = free **5**, blue = **5**/CT-DNA adduct). Right: absorbance spectrum ((open diamond = experimental, dashed red line = calculated) and relevant deconvolution (green = free **5**, blue = **5**/CT-DNA adduct). The bottom panels are the residuals.

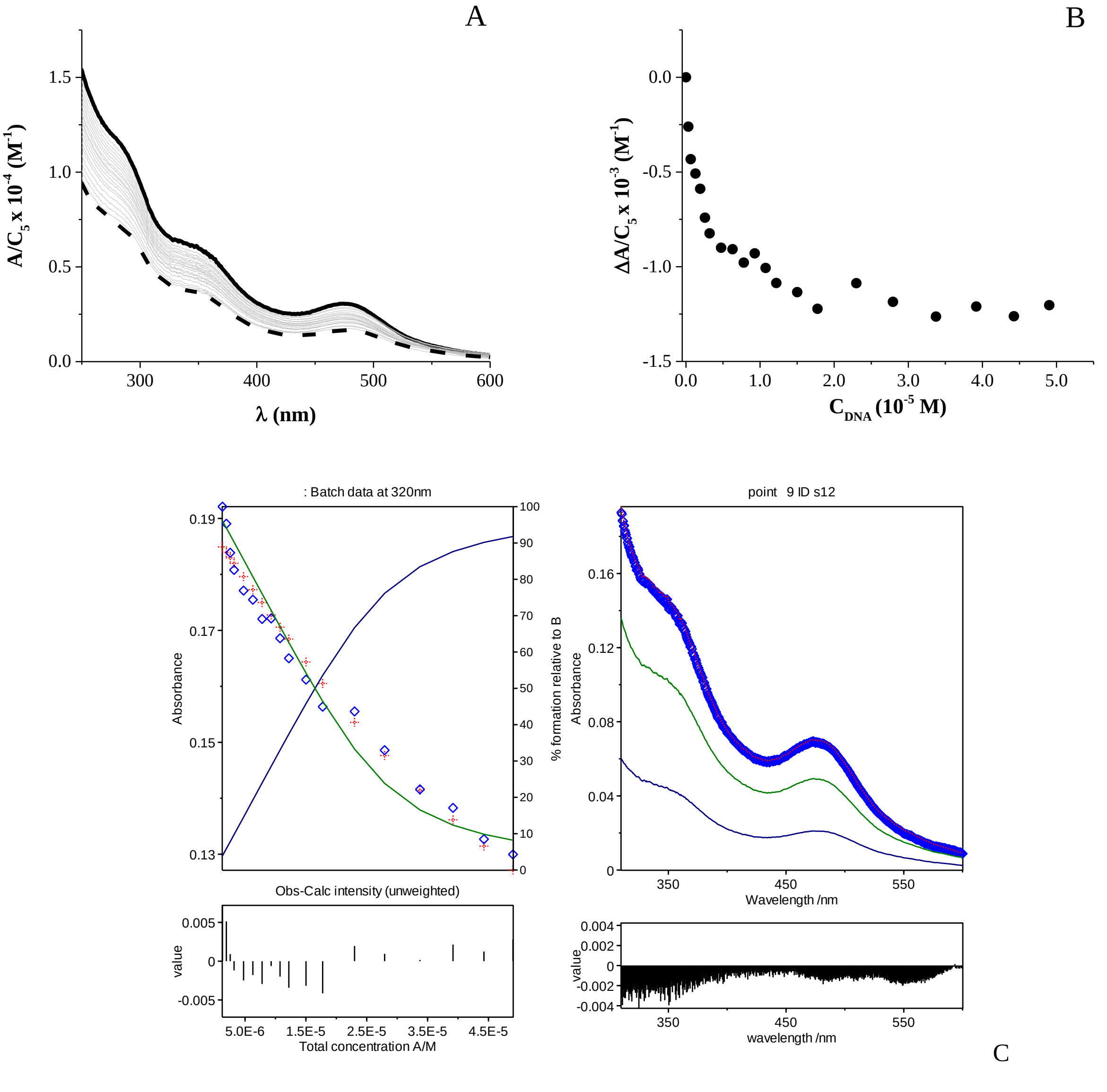

Figure S16. Absorbance spectra (A) and binding isotherm (B) at λ = 330 nm for the **6**/CT-DNA system: $C_6 = 2.28\times10^{-5}$ M, C_{DNA} = 0 M (–) to 4.88×10^{-5} M (- -); DMSO 1% v/v, NaCac 2.5 mM, pH = 7.0, T = 25.0°C. (C) HypSpec2014 analysis of the spectrophotometric titration. Left panel: titration curve at 330 nm (open diamond = experimental, cross = calculated) and species distribution (green = free **6**, blue = **6**/CT-DNA adduct). Right: absorbance spectrum ((open diamond = experimental, dashed red line = calculated) and relevant deconvolution (green = free **6**, blue = **6**/CT-DNA adduct). The bottom panels are the residuals.

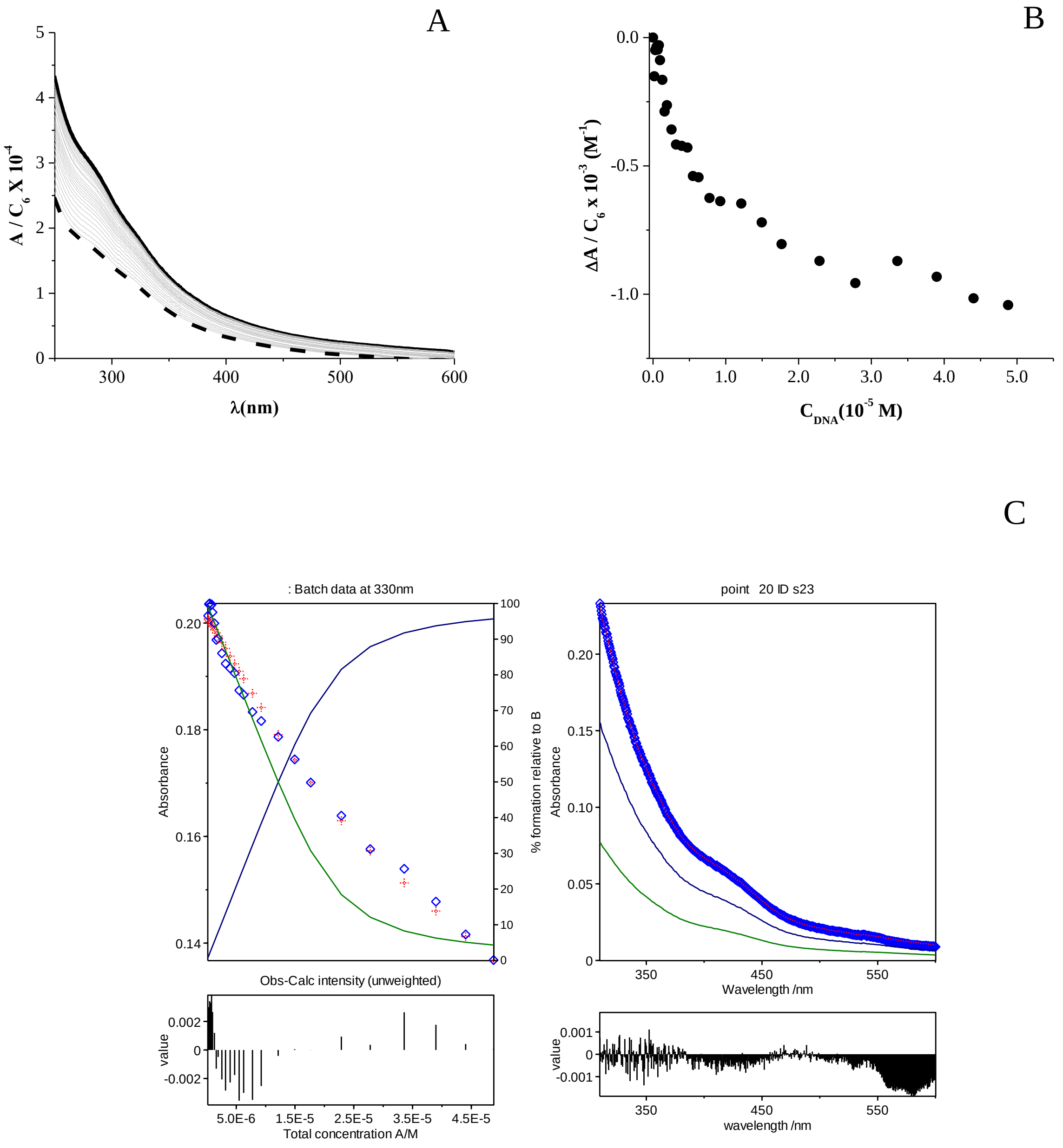

Figure S17. Absorbance spectra (A) and binding isotherm (B) at λ = 270 nm for the **7**/CT-DNA system: C_7 = 1.68×10^{-5} M, C_{DNA} = 0 M (–) to 4.98×10^{-5} M (- -); DMSO 1% v/v, NaCac 2.5 mM, pH = 7.0, T = 25.0°C.

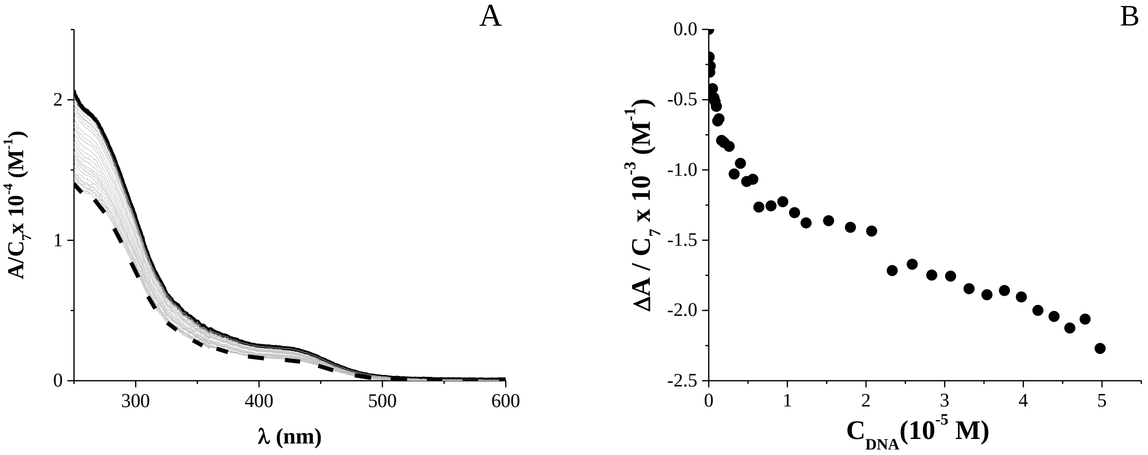

Figure S18. Ln(K) vs. 1/T (K^{-1}) plot for the **5**/CT-DNA and **6**/CT-DNA systems; DMSO 1% v/v, NaCac 2.5 mM, pH = 7.0.

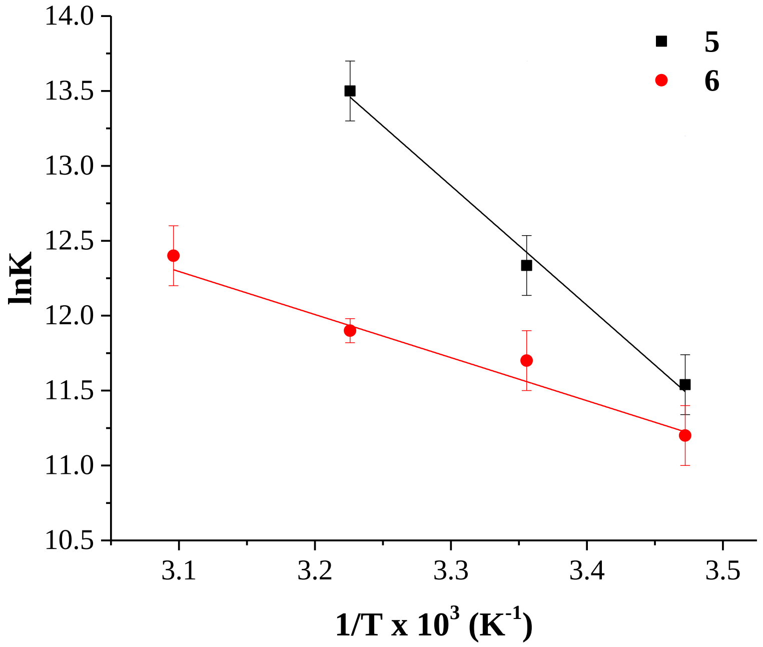

Figure S19. Absorbance decrease (F/F° %) observed upon addition of a metal complex to the EB/CT-DNA mixture; C_{DNA} = 3.81×10^{-5} M, C_{EB} = 1.34 × 10^{-5} M; NaCac 2.5 mM, pH = 7.0, T = 25.0°C, λ_{exc} = 520 nm, λ_{em} = 595 nm. Blank test means addition of buffer only.

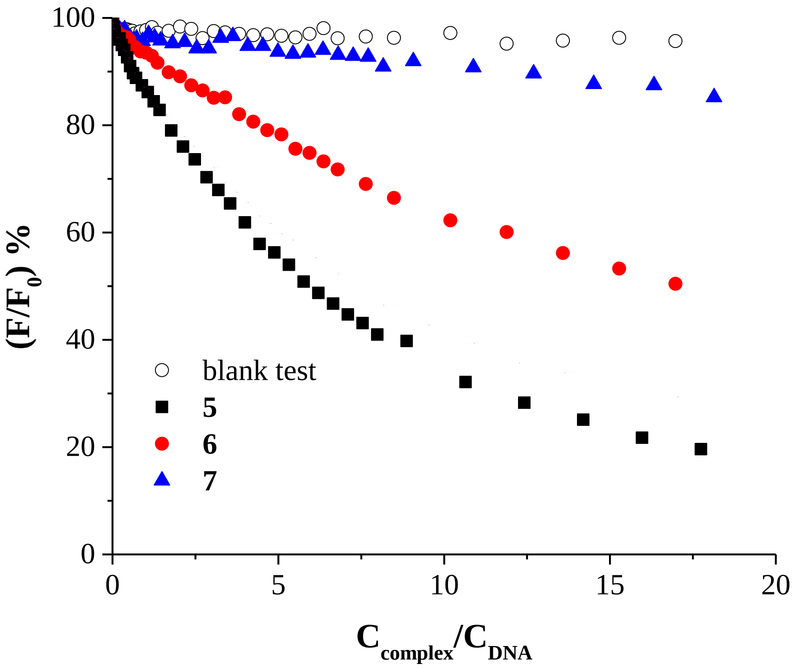

Figure S20. Absorbance changes with temperature at 260 nm for metal complexes/CT-DNA mixtures at 1:1 ratio; C_{DNA} = 4.9×10^{-5} M; DMSO 1% v/v, NaCac 2.5 mM, pH = 7.0. A% = (A - A_0) / (A_∞ - A_0).

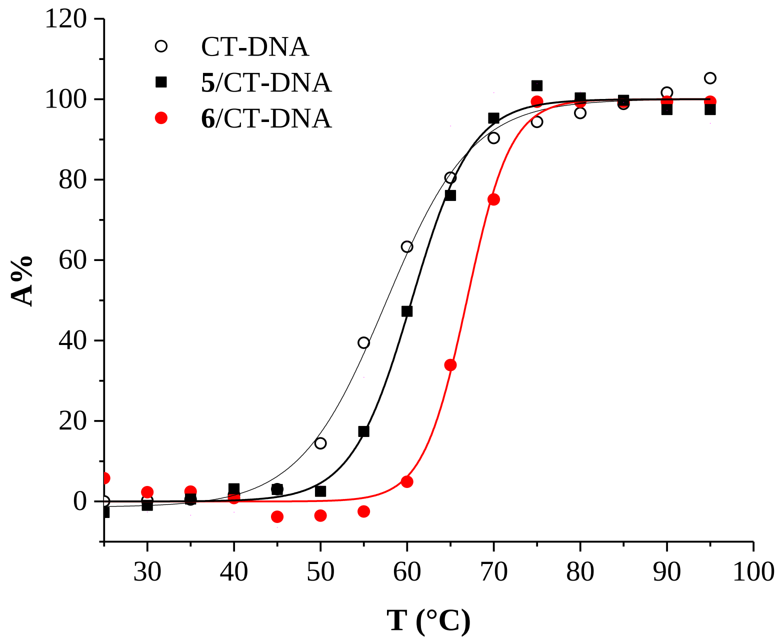

Figure S21. Absorbance spectra (A) and binding isotherm (B) at λ = 320 nm for the **5**/poli(rA)·poli(rU) system: C_5 = 3.05×10^{-5} M, C_{AU} = 0 M (–) to 5.10×10^{-5} M (- -); DMSO 1% v/v, NaCac 2.5 mM, pH = 7.0, T = 25.0°C. (C) HypSpec2014 analysis of the spectrophotometric titration. Left panel: titration curve at 320 nm (open diamond = experimental, cross = calculated) and species distribution (green = free **5**, blue = **5**/AU adduct). Right: absorbance spectrum ((open diamond = experimental, dashed red line = calculated) and relevant deconvolution (green = free **5**, blue = **5**/AU adduct). The bottom panels are the residuals.

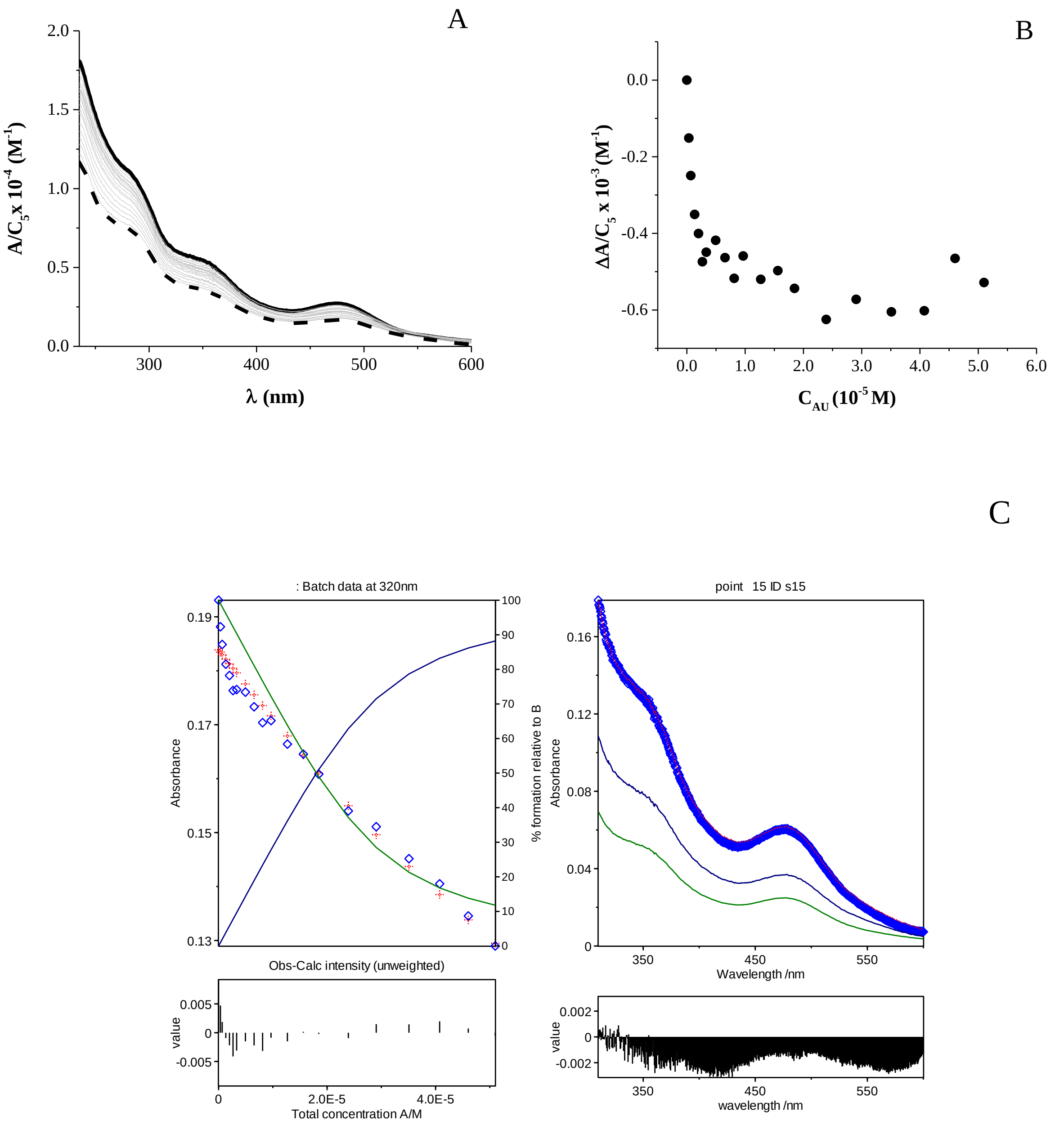

Figure S22. Absorbance spectra (A) and binding isotherm (B) at λ = 330 nm for the **6**/poli(rA)·poli(rU) system: C_6 = 2.77×10^{-5} M, C_{AU} = 0 M (–) to 4.90×10^{-5} M (- -); DMSO 1% v/v, NaCac 2.5 mM, pH = 7.0, T = 25.0°C. (C) HypSpec2014 analysis of the spectrophotometric titration. Left panel: titration curve at 420 nm (open diamond = experimental, cross = calculated) and species distribution (green = free **6**, blue = **6**/AU adduct). Right: absorbance spectrum ((open diamond = experimental, dashed red line = calculated) and relevant deconvolution (green = free **6**, blue = **6**/AU adduct). The bottom panels are the residuals.

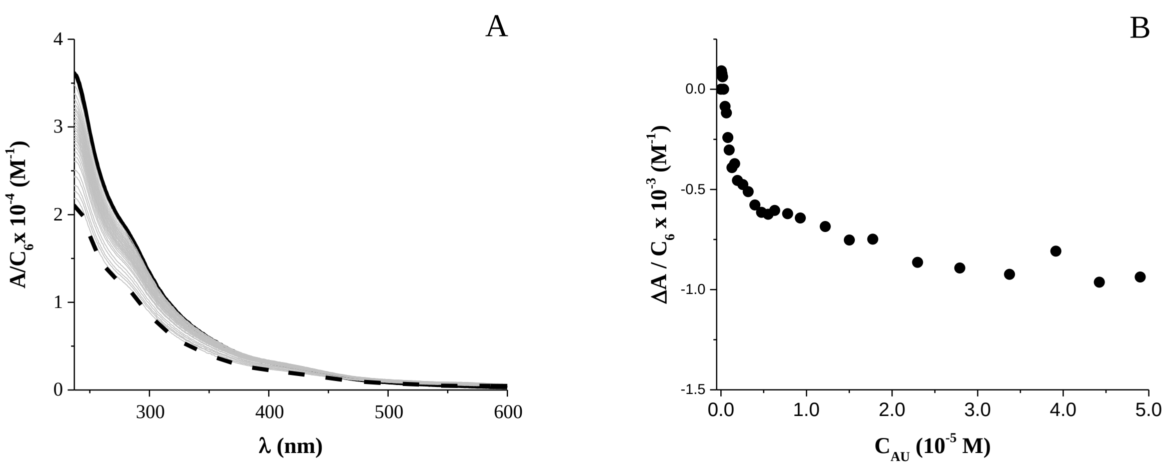

C

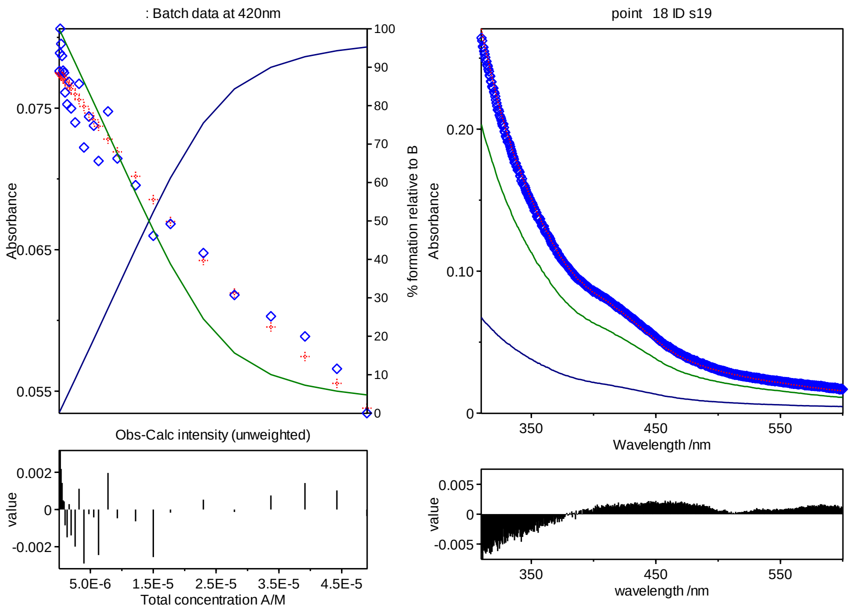

Figure S23. Absorbance spectra (A) and binding isotherm (B) at λ = 320 nm for the **7**/poli(rA)·poli(rU) system: $C_7 = 2.40\times10^{-5}$ M, C_{AU} = 0 M (–) to 4.89×10^{-5} M (- -); DMSO 1% v/v, NaCac 2.5 mM, pH = 7.0, T = 25.0°C. (C) HypSpec2014 analysis of the spectrophotometric titration. Left panel: titration curve at 330 nm (open diamond = experimental, cross = calculated) and species distribution (green = free **7**, blue = **7**/AU adduct). Right: absorbance spectrum ((open diamond = experimental, dashed red line = calculated) and relevant deconvolution (green = free **7**, blue = **7**/AU adduct). The bottom panels are the residuals.

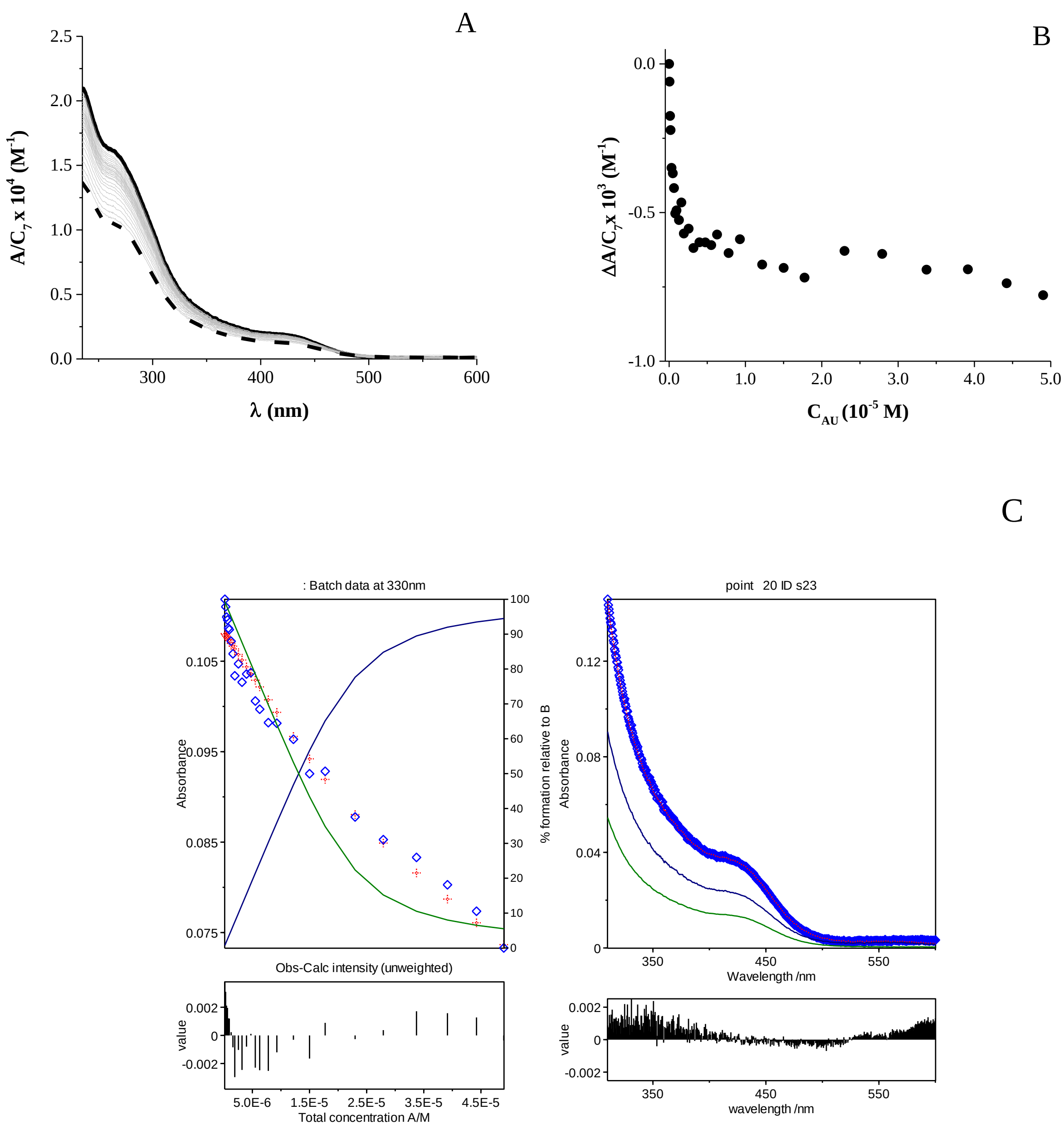

Figure S24. Absorbance spectra (A) and binding isotherm (B) at λ = 350 nm for the **5**/poli(rU)*poli(rA)·poli(rU) system: C_5 = 3.05×10^{-5} M, C_{UAU} = 0 M (–) to 8.80×10^{-5} M (- -); DMSO 1% v/v, NaCac 2.5 mM, pH = 7.0, T = 25.0°C. (C) HypSpec2014 analysis of the spectrophotometric titration. Left panel: titration curve at 350 nm (open diamond = experimental, cross = calculated) and species distribution (green = free **5**, blue = **5**/UAU adduct). Right: absorbance spectrum ((open diamond = experimental, dashed red line = calculated) and relevant deconvolution (green = free **5**, blue = **5**/UAU adduct). The bottom panels are the residuals.

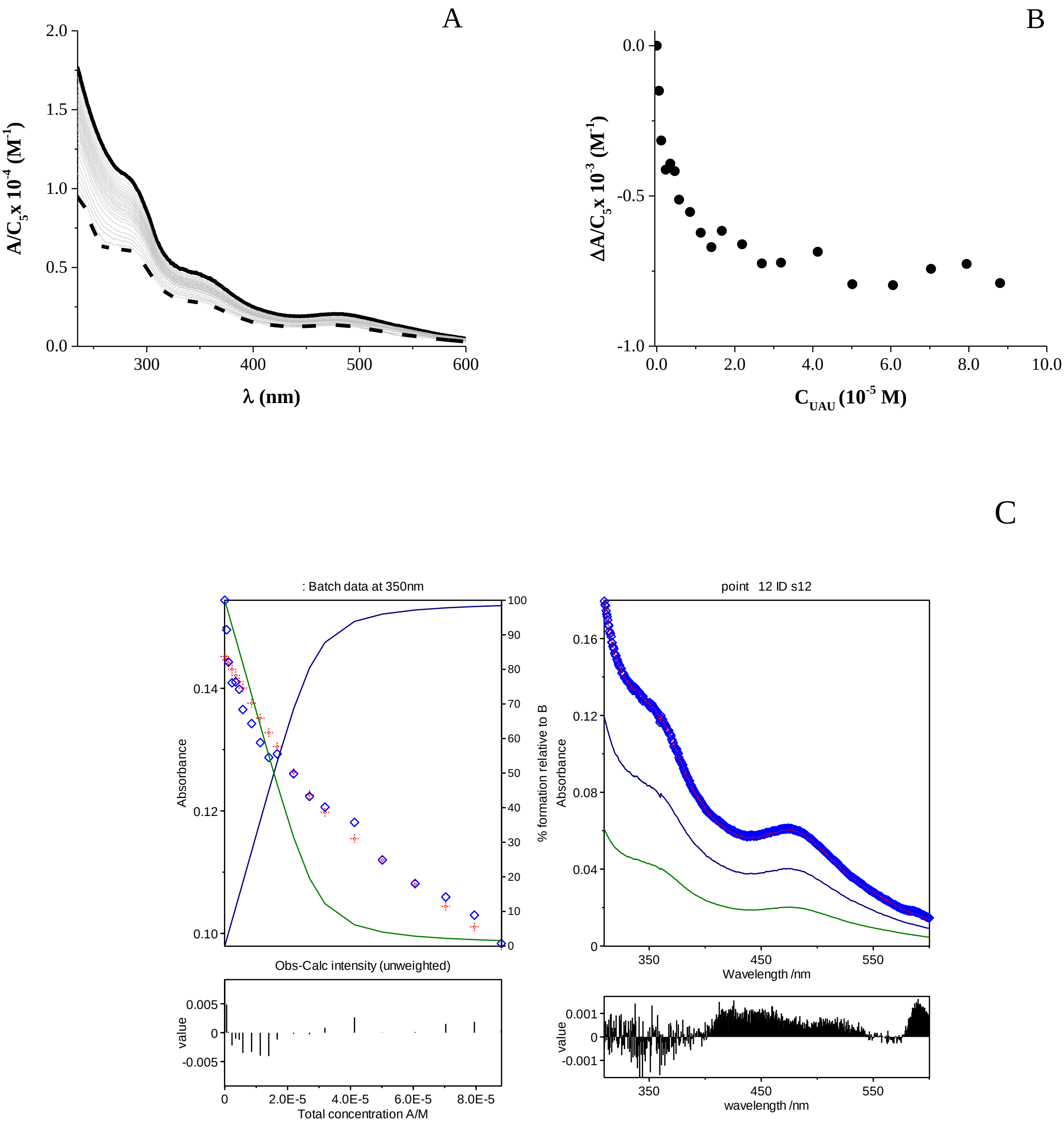

Figure S25. Absorbance spectra (A) and binding isotherm (B) at λ = 330 nm for the **6**/poli(rU)* poli(rA)·poli(rU) system: C_6 = 2.40×10^{-5} M, C_{UAU} = 0 M (–) to 4.90×10^{-5} M (- -); DMSO 1% v/v, NaCac 2.5 mM, pH = 7.0, T = 25.0°C. (C) HypSpec2014 analysis of the spectrophotometric titration. Left panel: titration curve at 350 nm (open diamond = experimental, cross = calculated) and species distribution (green = free **6**, blue = **6**/UAU adduct). Right: absorbance spectrum ((open diamond = experimental, dashed red line = calculated) and relevant deconvolution (green = free **6**, blue = **6**/UAU adduct). The bottom panels are the residuals.

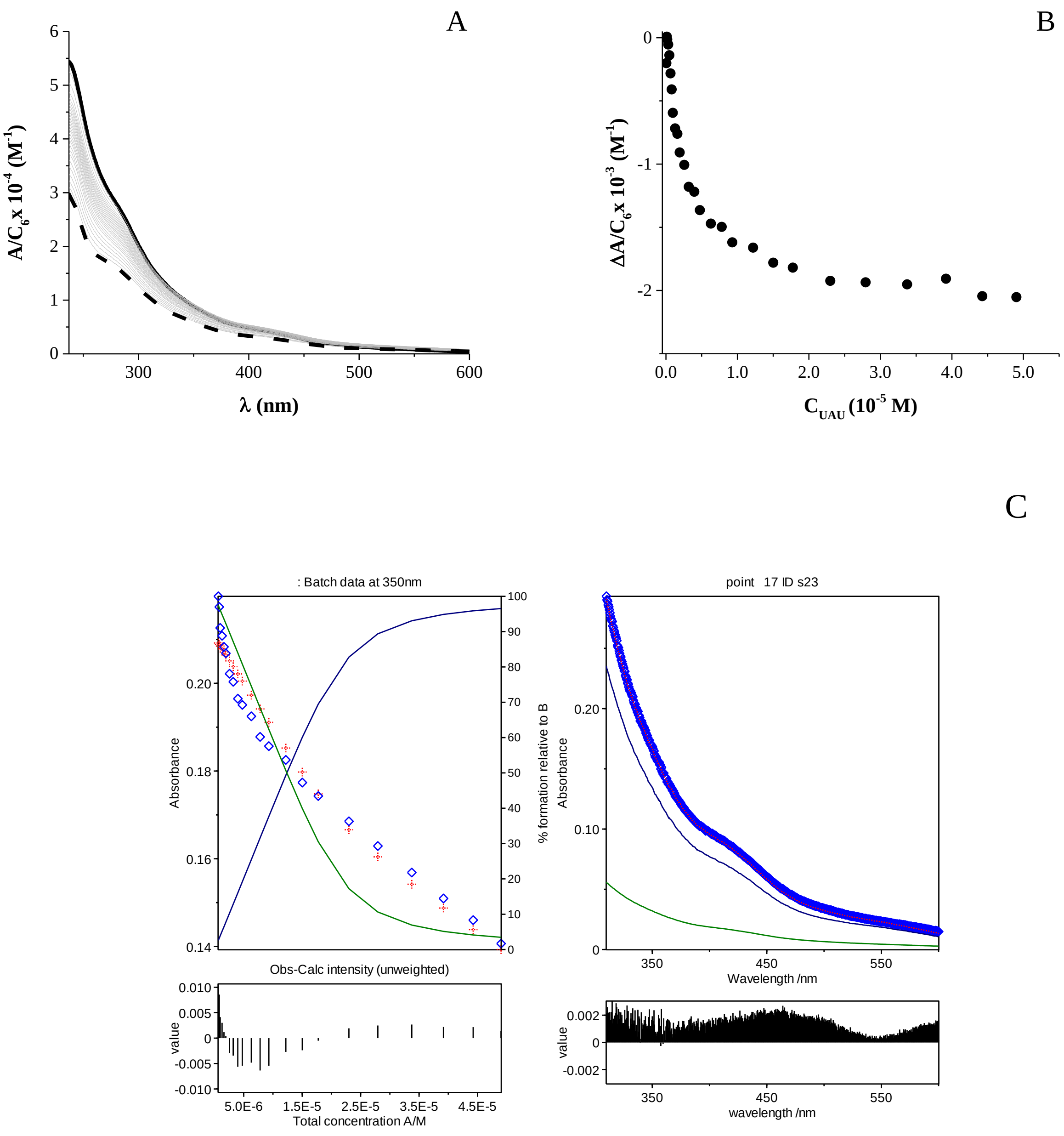

Figure S26. Absorbance spectra (A) and binding isotherm (B) at λ = 330 nm for the **7**/poli(rU)*poli(rA)·poli(rU) system: $C_7 = 2.19\times10^{-5}$ M, $C_{UAU} = 0$ M (–) to 8.80×10^{-5} M (- -); DMSO 1% v/v, NaCac 2.5 mM, pH = 7.0, T = 25.0°C. (C) HypSpec2014 analysis of the spectrophotometric titration. Left panel: titration curve at 340 nm (open diamond = experimental, cross = calculated) and species distribution (green = free **7**, blue = **7**/UAU adduct). Right: absorbance spectrum ((open diamond = experimental, dashed red line = calculated) and relevant deconvolution (green = free **7**, blue = **7**/UAU adduct). The bottom panels are the residuals.

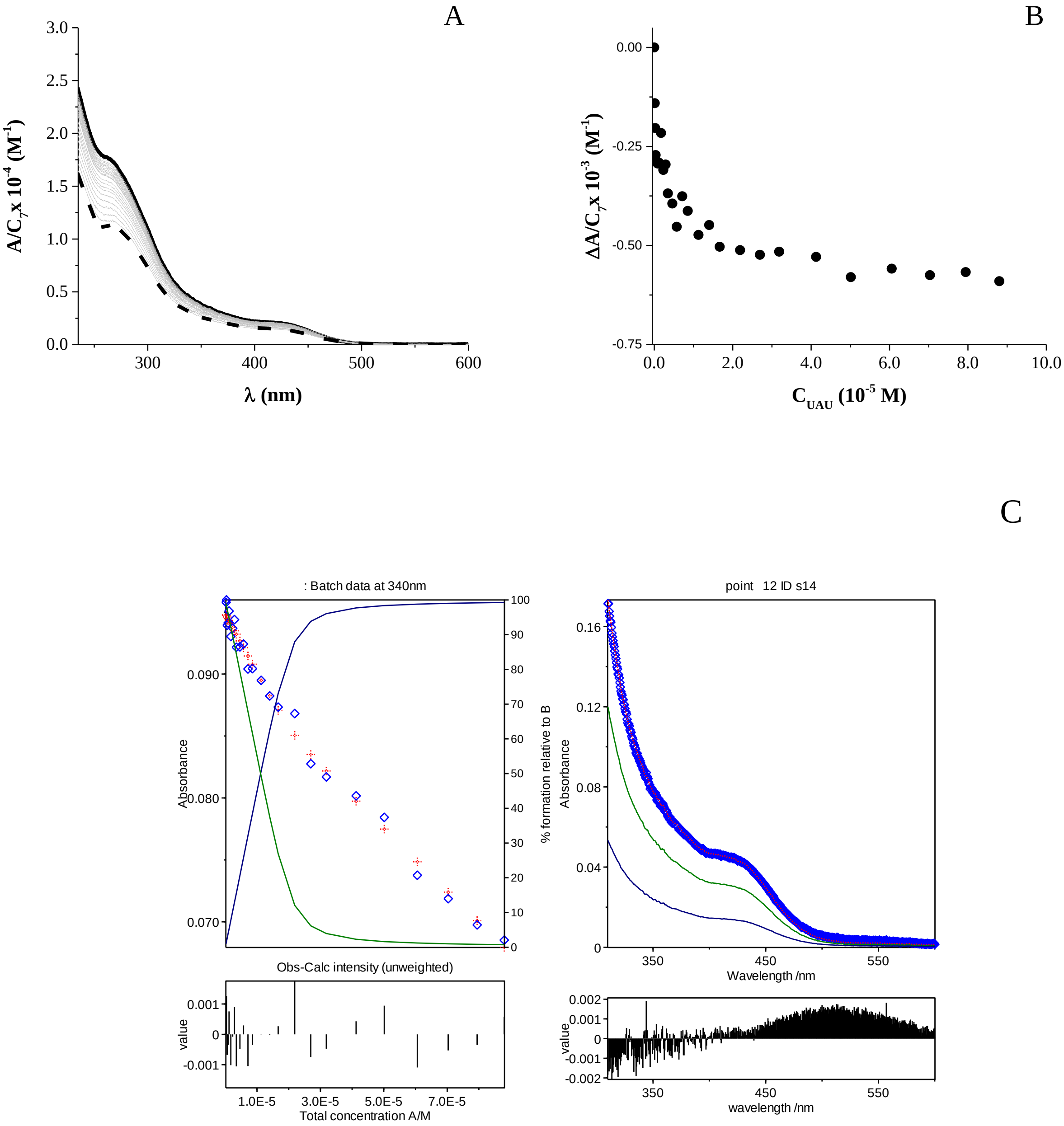

Table S2. Binding constants (K) obtained according to the HypSPec2014 software for the interaction between RNAs and the metal complexes and melting temperature changes at 1:1 ratio $C_{complex}/C_{polynucleotide}$; NaCac 2.5 mM, pH = 7.0. T_m(poli(A)·poli(U) = 47.9 ± 0.8 °C; T_m(poly(rU)*poly(rA)·poly(rU)) = 46.9 ± 0.3 °C.

		K (5)	K (6)	K (7)
poly(rA)·poly(rU)	15.0 °C	-	(5.4 ± 0.5)×10^5	(6.8 ± 0.9)×10^5
	25.0 °C	(3.0 ± 0.7)×10^5	(6.0 ± 0.9)×10^5	(5.4 ± 0.9)×10^5
	**ΔH**	> 0	≈ 0	≈ 0
	**ΔTm (°C)**	-6.2 ± 1.1	-6.5 ± 0.9	-8.7± 1.2
poly(rU)*poly(rA)·poly(rU)	25.0 °C	(1.0 ± 0.2)×10^6	(3.1 ± 0.9)×10^5	(1.0 ± 0.6)×10^7
	**ΔTm (°C)**	-3.7 ± 0.6	1.6 ± 0.5	-6.7± 0.6

Figure S27. Absorbance decrease (F/F° %) observed upon addition of a metal complex to the poli(rA)·poli(rU)/EtBr mixture; C_{AU} = 4.85×10^{-5} M, C_{EB}= 4.00×10^{-6} M; NaCac 2.5 mM, pH = 7.0, T = 25.0 °C, λ_{exc} = 520 nm, λ_{em} = 583 nm. Blank test means addition on buffer only.

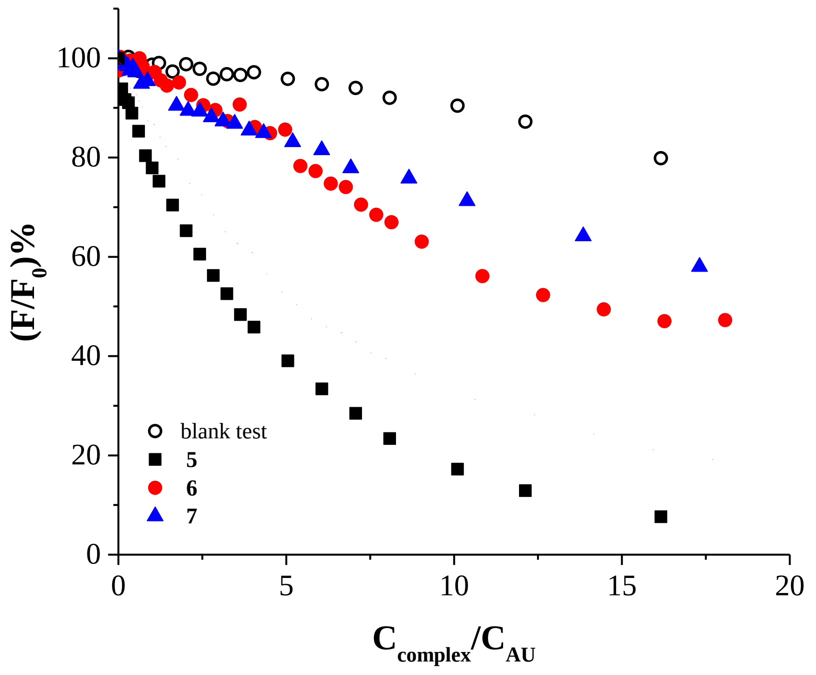

Figure S28. Absorbance changes with temperature at 260 nm for metal complexes/RNA mixtures at 1:1 ratio $C_{complex}/C_{polynucleotide}$; (A) poli(A)·poli(U), C_{AU} = 4.90×10^{-5} M; (B) poly(rU)*poli(A)·poli(U), C_{AU} = 4.90×10^{-5} M; DMSO 1% v/v, NaCac 2.5 mM, pH = 7.0.

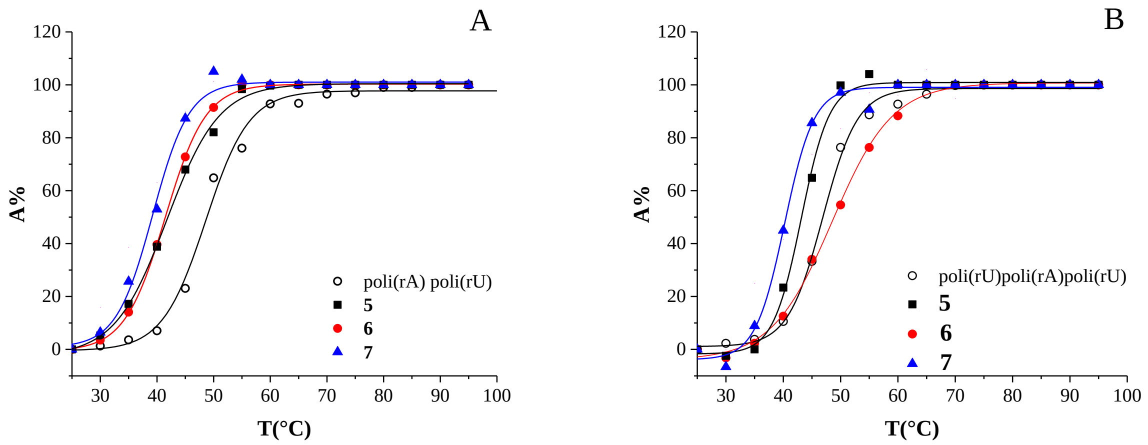

Figure S29. Fluorescence spectra (A) and binding isotherm (B) at λ_{em} = 340 nm for the **5**/BSA system: C_{BSA} = 5.11×10^{-7} M, C_5 = 0 M (–) to 1.23×10^{-6} M (- -); λ_{exc} = 280 nm, NaCac 2.5 mM, pH = 7.0, T = 25.0 °C. (C) HypSpec2014 analysis of the fluorometric titration. Left panel: titration curve at 340 nm (open diamond = experimental, cross = calculated) and species distribution (green = free **5**, blue = **5**/BSA adduct). Right: absorbance spectrum ((open diamond = experimental, dashed red line = calculated) and relevant deconvolution (green = free **5**, blue = **5**/BSA adduct). The bottom panels are the residuals.

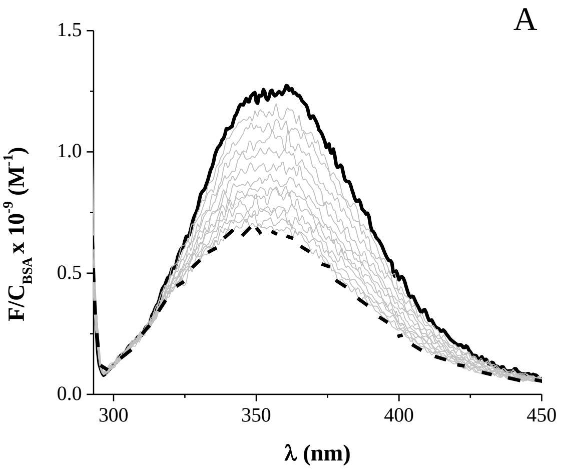

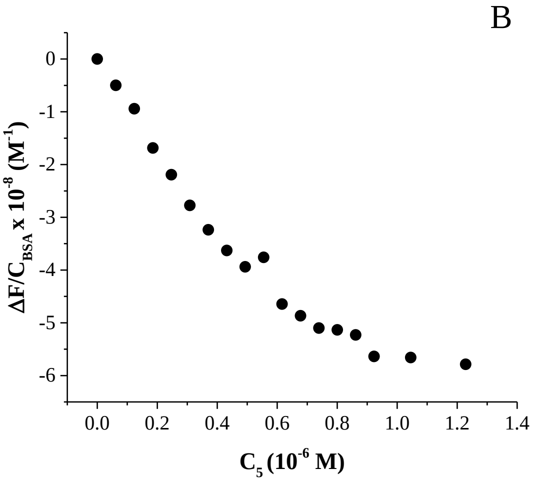

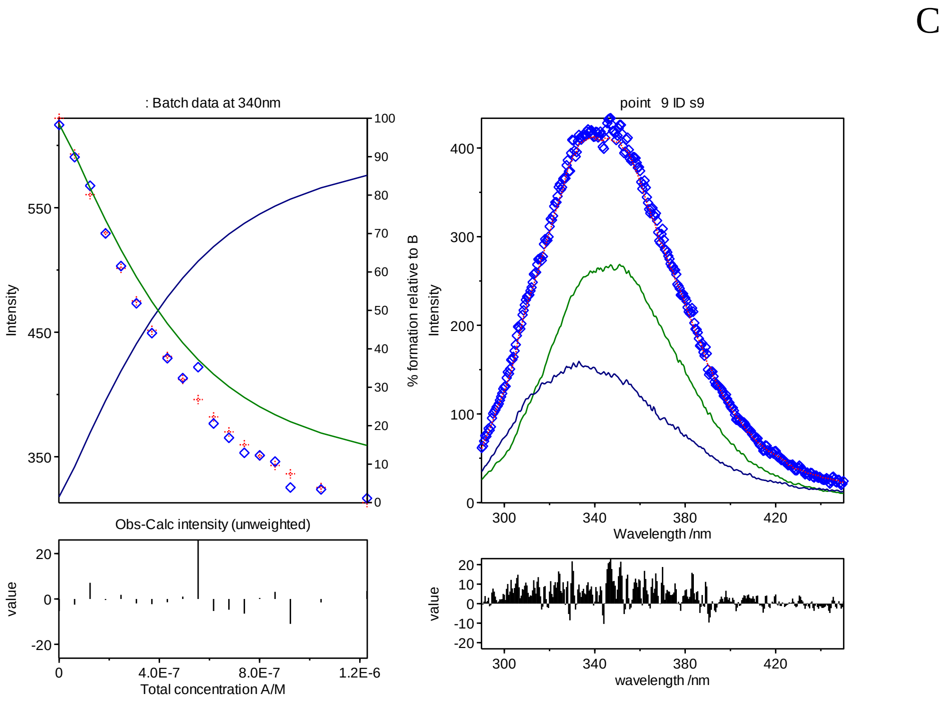

Figure S30. Fluorescence spectra (A) and binding isotherm (B) at λ_{em} = 340 nm for the **7**/BSA system: C_{BSA} = 5.11×10^{-7} M, C_7 = 0 M (–) to 3.54×10^{-6} M (- -); λ_{exc} = 280 nm, NaCac 2.5 mM, pH = 7.0, T = 25.0 °C. (C) HypSpec2014 analysis of the fluorometric titration. Left panel: titration curve at 340 nm (open diamond = experimental, cross = calculated) and species distribution (green = free **7**, blue = **7**/BSA adduct). Right: absorbance spectrum ((open diamond = experimental, dashed red line = calculated) and relevant deconvolution (green = free **7**, blue = **7**/BSA adduct). The bottom panels are the residuals.

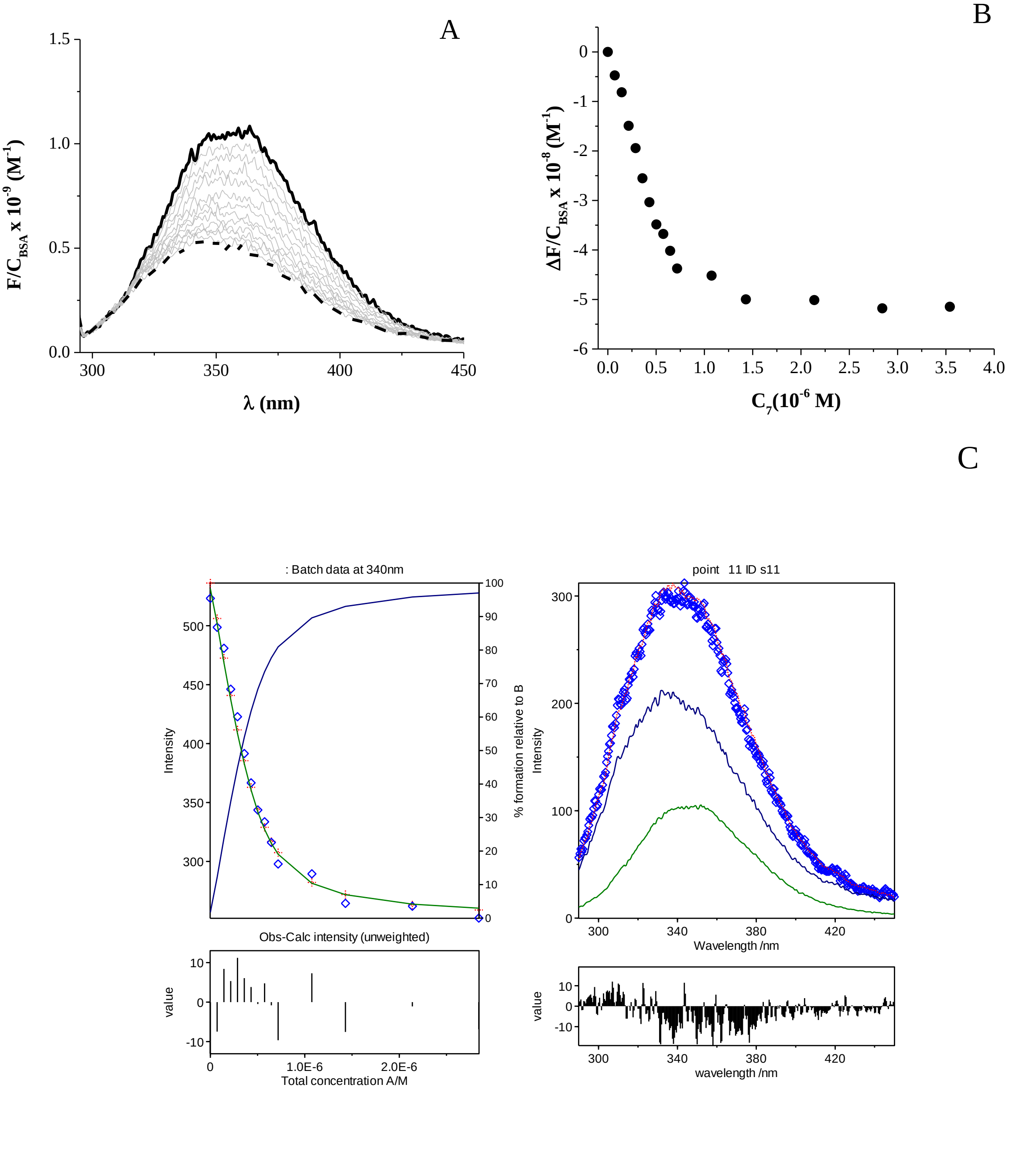

Figure S31. HypSpec2014 analysis of the fluorometric titration for the **6**/BSA system. Left panel: titration curve at 340 nm (open diamond = experimental, cross = calculated) and species distribution (green = free **6**, blue = **6**/BSA adduct). Right panel: absorbance spectrum ((open diamond = experimental, dashed red line = calculated) and relevant deconvolution (green = free **6**, blue = **6**/BSA adduct t). The bottom panels are the residuals. (A) 25.0 °C; (B) 37.0 °C. In this fitting procedure, only the first points of the titration (absorbance of **6** at 280 < 0.05) were used so to avoid inner-filter effects.

A

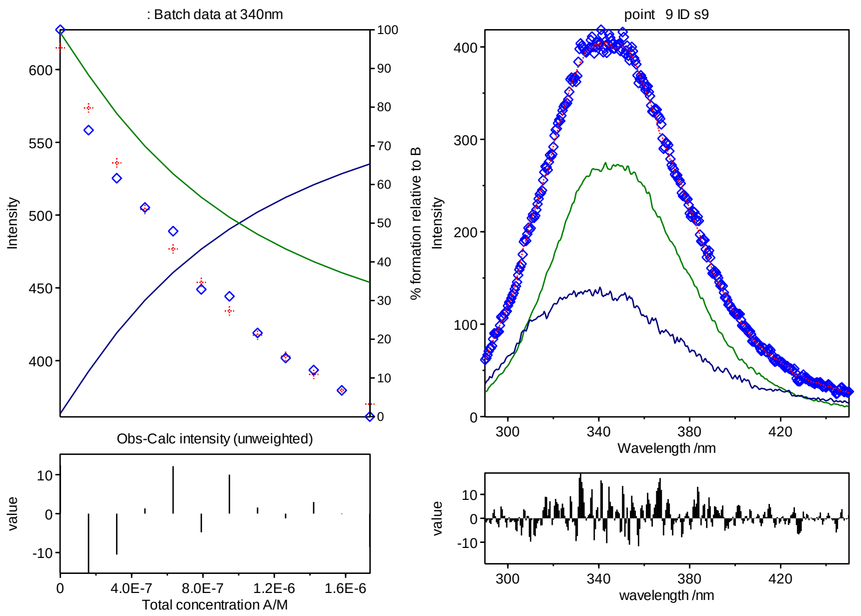

B

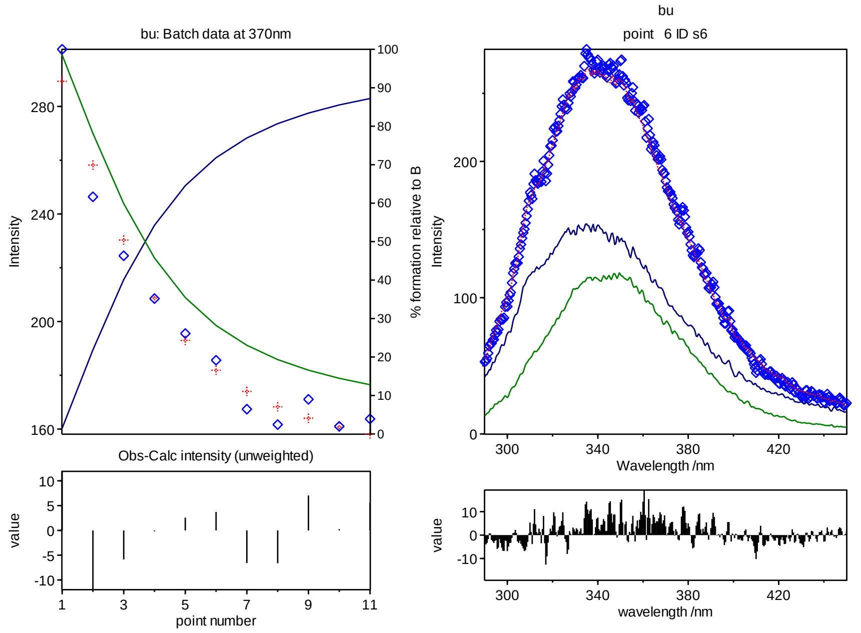

Table S3. Stern Volmer parameters (K_{SV}, fa) obtained by fitting the experimental data according to the modified Stern-Vomer equation (see below) for the metal complexes studied in this work. Binding constants (K_{BSA}) obtained according to the HypSPec2014 software for the interaction between BSA and the metal complexes at 1:1 stoichiometry. NaCac 2.5 mM, pH = 7.0.

	**5**		**6**		**7**	
T (°C)	**K_{SV} (M^{-1})**	**f_a**	**K_{SV} (M^{-1})**	**f_a**	**K_{SV} (M^{-1})**	**f_a**
25.0	$(9.9 \pm 0.2) \times 10^5$	0.9	$(4.0 \pm 0.1) \times 10^5$	0.7	$(2.7 \pm 0.1) \times 10^6$	0.8
37.0	$(6.0 \pm 0.1) \times 10^6$	0.9	$(5.8 \pm 0.1) \times 10^5$	0.7	$(1.7 \pm 0.5) \times 10^6$	0.5
T (°C)	**K_{BSA} (M^{-1})**		**K_{BSA} (M^{-1})**		**K_{BSA} (M^{-1})**	
25.0	$(7.3 \pm 0.1) \times 10^6$		$(1.1 \pm 0.3) \times 10^5$		$(1.4 \pm 0.1) \times 10^7$	
37.0	$(2.2 \pm 0.1) \times 10^7$		$(6.0 \pm 0.2) \times 10^6$		$(2.1 \pm 0.1) \times 10^7$	

The modified Stern-Volmer equation reads

$$\frac{F_0}{\Delta F} = \frac{1}{f_a K_{SV}[Q]} + \frac{1}{f_a}$$

where F_0 is the initial fluorescence of BSA alone, $\Delta F = F_0 - F$ where F is the fluorescence read at each addition of quencher (Q), [Q] is the concentration of free quencher in the system, K_{SV} is the Stern-Volmer constant for the quenching process, and f_a is the fraction of fluorescence accessible to the quencher. Note that, in the absence of quencher excess, $[Q] = C_Q - [Q]_{bound}$ is not known and needs to be calculated iteratively: (a) in a first step the plot is obtained assuming $[Q] = C_Q$ (with C_Q total analytical concentration of the quencher); (b) a first estimate of K_{SV} is obtained from the plot, $K_{SV} = [Q]_{bound}/([Q][P])$ where [P] is the unbound fraction of the protein $[P] = C_{BSA} - [Q]_{bound}$; (c) [Q] can be calculated at each point of the titration from K_{SV}, C_Q and C_{BSA} and a new plot is produced; (d) better K_{SV} (and f_a) estimates are obtained so to go back to step (c). The procedure is iterated until convergence.
